# Systematic review of sexual violence against sex workers: implications for mental and sexual health

**DOI:** 10.1186/s12889-026-28204-4

**Published:** 2026-06-30

**Authors:** Marie Püffel, İsmail Orbay, Ira Salo, Henriette Berg, Lea Hasanagic, Elisa Ruiz Burga, Thérèse Bernier, Nina Heinrichs

**Affiliations:** 1 Department of Psychology, Bielefeld University, Bielefeld, Germany; 2 Department of Social Work, Protestant University of Applied Sciences Berlin, Berlin, Germany; 3 Faculty of Law, University of Turku, Turku, Finland; 4 University College London, Institute of Global Health, London, UK; 5 Faculty of Applied Science, Construction and Engineering Technology, George Brown Polytechnic, Toronto, Canada

**Keywords:** Sex work, Sexual violence, Mental health, Sexual health, Systematic review, Meta-aggregation, Meta-analysis

## Abstract

**Background:**

Sex workers worldwide experience different forms of violence and stigma that increase the risk of adverse health outcomes. It is assumed that a discriminatory, criminalizing context as well as intersectional stigma increases the risk of sexual violence for sex workers. We aim to answer the following questions: (1) What types of sexual violence do sex workers experience and (2) how frequently do they encounter it? (3) To what extent are these types of sexual violence associated with mental and sexual health conditions? (4) Are these associations moderated by legislative models?

**Methods:**

In a preregistered systematic review (PROSPERO: CRD42024503922), scientific databases, references from meta-analyses, and publications from sex work organizations were searched. A total *k* = 207 quantitative and qualitative studies (*N* = 157,991) published since 2013 were summarized meta-analytically and narratively.

**Results:**

Sex workers experience sexual violence across various contexts, with quantitative records identifying rape as the most prevalent form. Qualitative findings document varied forms of sexual violence including non-penetrative acts, sexual harassment, technology-facilitated sexual violence, and sexual neglect. The lifetime prevalence of sexual violence is 26.3% (95% Confidence Interval [*CI*] 22.0–31.0). Mental health conditions (depressive, post-traumatic stress symptoms, and suicidality) were associated with sexual violence: OR [95% *CI*] = 1.85 [1.58–2.16], *p* < .001, as were alcohol and other recreational drugs: OR [95% *CI*] = 1.95 [1.59–2.38], *p* < .001. Both sexually transmitted infections, including HIV (OR [95% *CI*] = 1.36 [1.11–1.66], *p* = .003) and reproductive health related outcomes (OR [95% *CI*] = 1.76 [1.18–2.63], *p* = .013) were significantly associated with sexual violence. Potential moderation by legislative model could not be detected due to insufficient data.

**Conclusion:**

This systematic review suggests sexual violence against sex workers as a critical human rights issue. However, the generalisability of these findings is constrained by substantial statistical heterogeneity and limited representativeness within the current literature, which remains focused predominantly on cisgender women in in-person sex work. This calls for redefining sexual violence with input by sex workers and addressing structural inequities to aid in destigmatizing sex work and reducing social inequities.

**Supplementary Information:**

The online version contains supplementary material available at 10.1186/s12889-026-28204-4.

## Background

“Sex workers include female, male and transgender adults […] who receive money or goods in exchange for sexual services, either regularly or occasionally” (p. 1) [1]. Sex workers are a heterogeneous group regarding multiple social categories and positions (e.g., gender identity, race or ethnicity, socioeconomic background, provided services) and are faced with various contextual factors such as varying legislative models across the world. Since sex workers are marginalised world-wide and experience stigma and discrimination, these structural determinants might increase their vulnerability to sexual violence [2, 3]. The meta-analysis by Deering and colleagues [4], including 41 quantitative studies, indicates a 12-months-prevalence of workplace sexual and/or physical violence of 32 to 55% and experienced sexual violence from any (paying or non-paying) partner of 8 to 19% within the last year.

“Sexual violence is defined as any sexual, attempt to obtain a sexual act, […] or otherwise directed [act] against a person’s sexuality using coercion” (p. 149) [5]. In this review, we extend the definition of sexual violence to include institutional omissions and systematic failures such as the denial of protection, appropriate care, and access to justice, that undermine the sexual autonomy and bodily integrity of sex workers. Termed here as ‘sexual neglect’, this framework builds upon existing research regarding stigma and structural violence against sex workers [3, 6] as well as sexual justice, defined by The World Association for Sexual Health (WAS) as the realisation of sexual rights as human rights through dignity, bodily autonomy, non-discrimination, and equitable access to care and legal protection [7]. By doing so, we emphasize the rights-based dimension of sexual violence that transcends individual behavioural acts to include institutional endangerment of sexual health, well-being and the right to free expression of sexual orientation and gender identity [8]. Sexual violence has been proven to be a fundamental risk factor for multiple negative health outcomes, including related to mental health [9, 10] and sexual health [11, 12]. These adverse effects are exacerbated by experienced barriers in access to health care for sex workers [13, 14] as well as barriers in reporting their experiences of violence by lack of acceptance of such reports and victim blaming [15]. The nature of these barriers is shaped by the contextual circumstances sex workers face, specifically the legislation surrounding sex work. The model of decriminalization is advocated for by sex worker communities, scholars, and human rights institutions alike and is associated with less violence and better health outcomes for sex workers [16–19]. Nevertheless, a majority of the countries world-wide have legislative models which at least partially criminalise sex work [20] and ongoing discussions about sex work legislation (e.g., in the European Union [21]) shift towards the criminalisation of clients. Policy debates are frequently influenced by persistent misconceptions and myths, e.g., conflating consensual adult sex work with trafficking, assuming violence is intrinsic rather than structurally produced, or presuming that punitive approaches necessarily improve safety [6, 22]. In addition, moralising views on sex work potentially intensify stigma and punitive enforcement [23, 24].

Together, these dynamics can distort debate and hinder evidence-informed policy. This meta-analysis and meta-aggregation review aims to shed light and to provide a differentiated overview on sexual violence experienced by sex workers, as well as its association with their health outcomes along different legislative models. While Deering and colleagues [4] already meta-analytically described correlates of violence experienced by sex workers, they neither focused on the broad range of sex work or sexual violence nor on the health outcomes associated with these experiences. Furthermore, their review only analysed three studies for lifetime sexual violence prevalence and one study for past year prevalence. Additionally, by the integrating of both quantitative and qualitative studies, we are able to contextualise prevalences with experiential data, thereby generating more comprehensive insights. While involving sex workers in distinct stages of this research, we focus on the following research questions: (1) What types^1^ of sexual violence are experienced by sex workers? (2) What are the prevalences of sexual violence differentiated by the type^2^ of sex work and/or contexts^3^ of sexual violence? (3) To what extent is sexual violence associated with mental (3a) and sexual (3b) health? (4) Are there differences associated with the legislative model^4^?

## Methods

This systematic review, meta-analysis and meta-aggregation was pre-registered on PROSPERO (CRD42024503922) and followed PRISMA-guidelines.

### Search strategy and selection criteria

Publication sources include following scientific data bases: EMBASE, MEDLINE (Complete), PsycArticles, PsychInfo, PSYNDEXplus, PubMed, PubPsych, Scopus and Web of Science. Additionally, sex workers’ organisations were asked and their websites were searched to add further articles and grey literature. The search was conducted between the 22nd and 24th of January 2024 and updated on the 6th of August 2025. An example search strategy string is provided in the Additional file 1.

For study management and selection process, we used *Covidence* [25]. All studies were assessed independently regarding the inclusion and exclusion criteria through each phase of selection by MP and another author or research assistant (title/abstract and full-text). Discrepancies were discussed to find consensus. Studies were included, when (1) sex workers were participants, defined by above stated definition by the Global Network of Sex Work Projects (NSWP) [1], thereby including a wide range of sexual services; (2) sample mean or median age was 18 or higher; and (3) any kind of sexual violence was assessed. Studies with and without health outcome, any study designs including empirical data and no language restrictions were applied.

Studies were excluded if they focused (1) on survivors of sex trafficking, sexual violence survivors only (the latter for quantitative studies) or (2) underage population; (3) had no measure on sexual violence experienced as an adult (since age of 18); (4) were reviews or publications without empirical data; and (5) if full-texts were not available. A post-hoc selection process excluded studies published before 2013 and those included in Deering and colleagues [4] to keep the amount of included studies feasible and less redundant to the review by Deering and colleagues. Non-English studies were eligible for inclusion to minimise language bias. In total, *n* = 10 studies were translated into English using DeepL for full-text review (Spanish, Portuguese, and Russian). Three non-English full texts (Spanish *n* = 2, Portuguese *n* = 1) were translated for quality assessment and (where relevant) data extraction. One Spanish-language qualitative study was excluded following quality assessment, and two non-English studies were included in the final synthesis (one Portuguese qualitative; one Spanish quantitative).

### Data analysis

Extracting data from studies was done independently using Covidence [25] by MP and trained research assistants. Differences were discussed to find consensus. Multiple publications of same studies were merged. Following, we describe data extraction and analysis for quantitative and qualitative studies separately. For mixed-method studies, both approaches were applied. For all studies, we extracted study characteristics and sample characteristics including various sociodemographic domains, details are going to be published in Püffel et al. (under review). The meta-aggregation was used to answer the first objective. The meta-analysis provided data for objectives two, three and four.

#### Meta-aggregation of qualitative studies (Obj. 1)

For qualitative studies, the data extraction was based on the Joanna Briggs Institute (JBI) methodology for qualitative systematic reviews [26]. Findings from qualitative studies were categorized into three plausibility levels (unequivocal, equivocal, unsupported) based on their supporting illustrations (i.e., direct quotes) and coded based on type of sexual violence and context or perpetrator. Since most of the studies only included terms like “sexual violence” and “sexual abuse” in their finding, divergent from the JBI methodology, we used illustrations to gather a more detailed overview about types and contexts of sexual violence. Additionally, the definition of sexual violence was extended to include ‘sexual neglect’, an inductively derived meta-aggregated category (i.e., developed during synthesis, not pre-specified). All studies were extracted by MP and a second author (LH, HB), consensus was reached by discussion. Themes and subcategories were developed from the unequivocal and equivocal findings (Obj. 1). Thereby, the taxonomy of intimate partner sexual violence by Bagwell-Gray and colleagues [27] was applied and adapted. Qualitative studies were assessed through the checklist for qualitative research by JBI [26], whereby low-quality studies were excluded for meta-aggregation to minimise bias.

#### Meta-analysis of quantitative studies (Obj. 2–4)

We extracted data on sexual violence, mental and sexual health outcomes as well as potential moderators. Study quality and bias of quantitative studies was assessed through the Quality Assessment Tool for Observational Cohort and Cross-Sectional Studies [28]. Based on the categories by Platt and colleagues [16] and using time and country of data collection, legislative models were sorted into following categories: full criminalisation, criminalisation of purchase of sex, partial criminalisation, regulatory models, full decriminalisation, and not specified. In the case of studies that included multiple countries, a decision was based on whether the countries had the same type of regulatory models (marked according to the model) or not (marked as not specified). We classified legislative models based on “law in books” rather than enforcement (“law in action”). Accordingly, our categorisation may not capture variability in implementation, policing practices, or subnational legal differences [29].

All meta-analytical calculations were done using R Studio [30] and the metafor package [31]. Since many studies had multiple estimators for sexual violence and health outcomes, a multi-level-analysis was applied to group each effect size by study. The primary pooled prevalence estimate represents any sexual violence only (broadly defined) as operationalised in the primary studies, with an emphasis on penetration-type sexual assault where definitional detail was available. To obtain a single, interpretable summary estimate and to minimise sparse data across many small definitional subgroups, we pooled prevalence estimates from: (1) studies defining sexual violence as completed unconsented penetration (anal, oral, or vaginal) and/or non-consensual condom-removal (*k* = 86 studies, 41.0%), (2) studies without clear definition on sexual violence (*k* = 81, 38.6%), (3) studies assessing multiple types of sexual violence without using a validated scale (*k* = 16, 7.6%), and (4) studies using a validated scale (*k* = 20, 9.5%) were combined. Sexual harassment and forced entry into sex work were not included in this pooled prevalence and were analysed separately. Prevalence estimates where sexual violence was only reported in combination with other forms of violence were also synthesised separately as mixed types of violence. Meta-analyses were conducted separately for each reference period (lifetime, past year, past 6 months) and for each context (e.g., workplace, police). For the overall prevalence estimate within each reference period (and within each type of violence), prevalence estimates were pooled across all contexts.

Regarding health outcomes, we separated them into four categories: (1) mental health conditions (including e.g., depressive, anxiety, and post-traumatic symptoms as well as suicidal ideation or behaviour), (2) alcohol and other drug use, (3) sexually transmitted infections (STIs) including HIV, as well as (4) reproductive health-related conditions (e.g., unwanted pregnancy, miscarriages, abortions). Associations between sexual violence and health conditions reported as prevalence and risk ratios were transformed into odds ratios using the calculated prevalence of health outcomes of sexual violence-unexposed participants (P0). We calculated sexual violence prevalences and odds ratios using logarithmic transformation and 95-%-confidence intervals [CI]. For health condition associations (odds ratios), sexual violence only and mixed types of violence were combined, and type of violence was examined in subgroup analyses (Additional file 2). A random-effects model using a restricted maximum likelihood (REML) estimator was applied, using the Knapp-Hartung method [32], to adjust the test statistics and confidence intervals, providing more reliable estimates in the presence of high heterogeneity. In the moderation analysis of legislative models, the multi-level-analysis was applied to group each effect size by study and by World Health Organisation’s (WHO) designated region (low- and middle-income countries and areas in African Region, Region of Americas, Eastern Mediterranean Region, European Region, South-East Asia Region, Western Pacific Region; High-income countries and areas; Unspecified region/area) used in the Violence Against Women Prevalence Estimate from 2018 [33]. Further subgroup analyses were conducted to find sources of heterogeneity and included study quality, legislative models, contexts of sexual violence and – in case of odds ratios – health outcomes and type of violence (sexual violence only or mixed types of violence; presented in Additional file 2). Funnel plots and Egger’s tests (Additional file 2) visualised publication bias and asymmetry. I^2^ and Cochrane’s Q were used to analyse heterogeneity.

### Stakeholder involvement

Biases do not only include the underrepresentation of non-significant results, but also the underrepresentation of content produced by people with lived experience [34]. To ensure relevance and sensitivity of this review, we engaged sex workers, advocates, and researchers as stakeholders at two key stages: during the initial conceptualisation of the review and through the interpretation of the findings. In the first stage, two sex workers were recruited via German sex work organizations for individual consultations (1–2 h each). They provided feedback on the research questions and design, while advising on the selection and relevance of candidate subgroup analyses. These consultations directly informed our analytic plan, including a priori attempts to extract and synthesise prevalence estimates by type of sex work. However, this could ultimately not be meta-analysed due to limited reporting and insufficient variability across included studies. Second, following the initial analyses, we conducted two additional discussion rounds for the findings presented in this publication with eight sex workers, researchers, and advocates from six countries, reached via sex work and research organisations across the world. These discussions informed the interpretation of the results, including the subdivision into four health domains. Additionally, we included literature from sex workers’ organisations and analysed whether involvement of sex workers were reported in included studies (details in the accompanying paper, Püffel et al., under review).

## Results

**Fig. 1:**
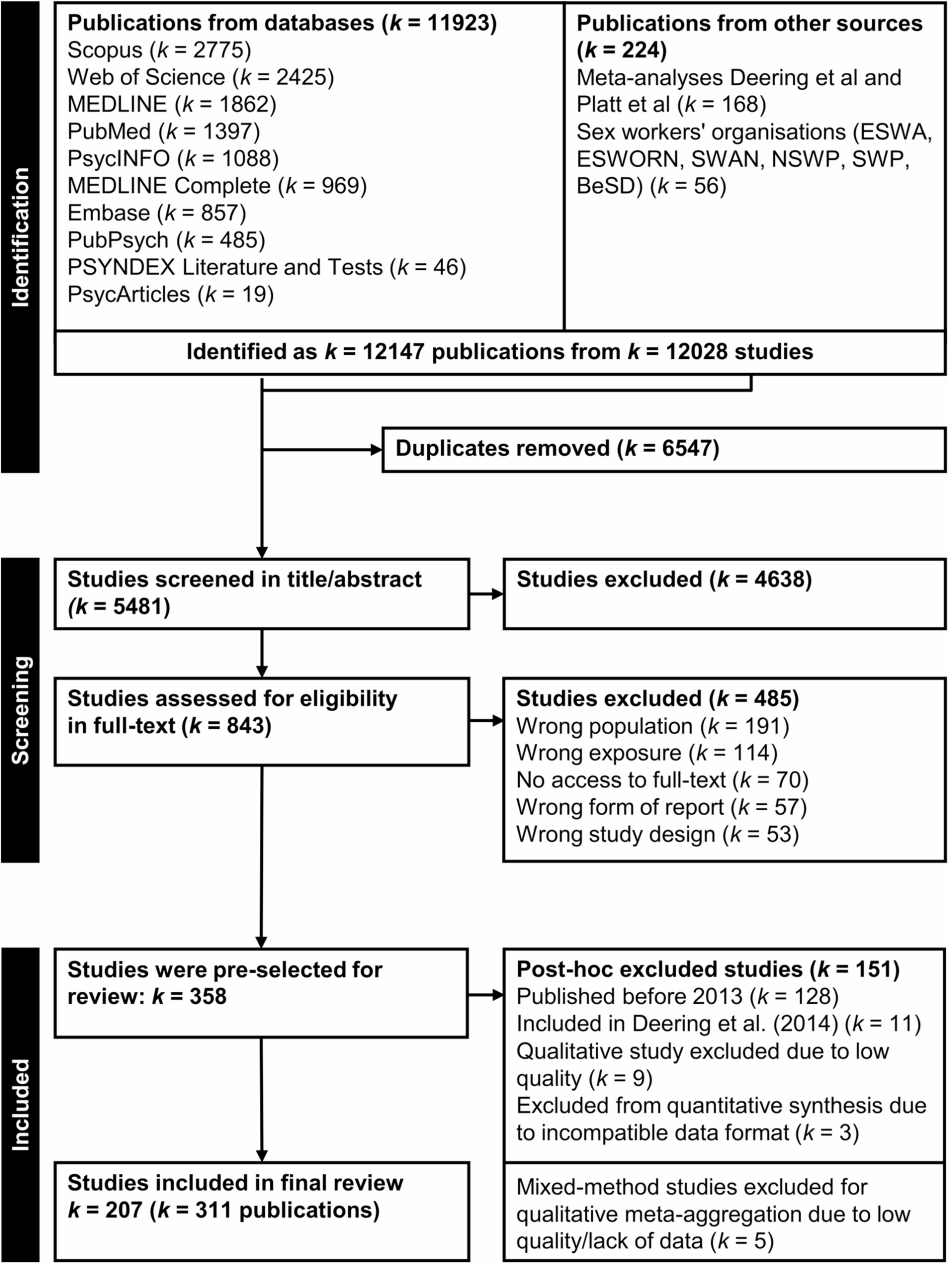
PRISMA Chart. Note. ESWA = European Sex Workers‘ Rights Alliance, ESWORN = European Sex Work Research Network, SWAN = Sex Workers’ Advocacy Network, NSWP = Global Network of Sex Work Projects, SWP = The Sex Workers Project, BesD = Berufsverband für erotische und sexuelle Dienstleitungen [Professional Association for Erotic and Sexual Service Providers]

A total of *K* = 5,482 studies were screened by title/abstract, *k* = 8434 were reviewed in full-text. In final selection, *k* = 311 publications from *k* = 207 studies published since 2013 from 98 different countries, were analysed (see Fig. 1). The total sample encompassed *N* = 157,991 sex workers overall. An overview of included studies is given in Table 1 (more details about each study in Additional file 3). A majority of studies (69.1%, *k* = 143) focused on either cis women or sex workers described as female, while 20.8% of studies (*k* = 43) included multiple genders. Concerning sex work characteristics, 72.9% (*k* = 151) of studies included participants exchanging sex (or sexual services) against money or goods in-person, 24.2% (*k* = 50) did not provide any definition of sex work. If street-based setting was surveyed (*k* = 72), 37.6% of participants (24,456 of 65,067) had worked in street/ outdoor setting. More details about sociodemographic in Püffel et al. (under review).

**Table 1:** Overview of included studies

Categories	Overall *K* = 207 (100%)	Quantitative studies: *k* = 142 (68.6%)	Qualitative studies: *k* = 50 (24.2%)	Mixed-Methods studies: *k* = 15 (7.3%)
WHO assigned region				
Low- and middle-income countries and areas in:				
African Region	86 (41.5%)	63 (44.4%)	22 (44%)	1 (6.7%)
Region of the Americas	26 (12.6%)	21 (14.8%)	3 (6%)	2 (13.3%)
Eastern Mediterranean Region	5 (2.4%)	5 (3.5%)		
European Region	5 (2.4%)	5 (3.5%)		
South-East Asia Region	16 (7.7%)	11 (7.7%)	3 (6%)	2 (13.3%)
Western Pacific Region	12 (5.8%)	9 (6.3%)	2 (4%)	1 (6.7%)
High income countries	54 (26.1%)	28 (19.7%)	19 (38%)	7 (46.7%)
Unspecified^a^	3 (1.4%)		1 (2%)	2 (13.3%)
Legislative model				
Full criminalisation	98 (47.3%)	70 (49.3%)	23 (46%)	5 (33.3%)
Criminalisation of purchase of sex	15 (7.2%)	7 (4.9%)	5 (10%)	3 (20.0%)
Partial criminalisation	65 (31.4%)	51 (35.9%)	13 (26%)	1 (6.7%)
Regulatory models	6 (2.9%)	4 (2.8%)		2 (13.3%)
Full decriminalisation	1 (0.5%)		1 (2%)	
Not specified	22 (10.6%)	10 (7%)	8 (16%)	4 (26.7%)
Quality assessment (only quantitative studies)				
Good	80 (38.6%)	74 (52.1%)		6 (40%)
Fair	60 (29%)	56 (39.4%)		4 (26.7%)
Poor	14 (6.8%)	12 (8.5%)		2 (13.3%)
Study design				
Cohort study	21 (10.1%)	19 (13.4%)		2 (13.3%)
Cross sectional study	112 (54.1%)	105 (73.9%)		7 (46.7%)
Intervention study	3 (1.4%)	3 (2.1%)		
Non-randomised experimental study	3 (1.4%)	2 (1.4%)		1 (6.7%)
Qualitative research	54 (26.1%)		49 (98%)	5 (33.3%)
Randomised controlled trial	11 (5.3%)	11 (7.7%)		
Unknown	3 (1.4%)	2 (1.4%)	1 (2%)	

Note: *k* number of studies, *WHO* World health organisation

^a^ = due to multiple countries in multiple regions

### Results from qualitative studies

#### Obj. 1: Types of sexual violence experienced by sex workers

**Fig. 2:**
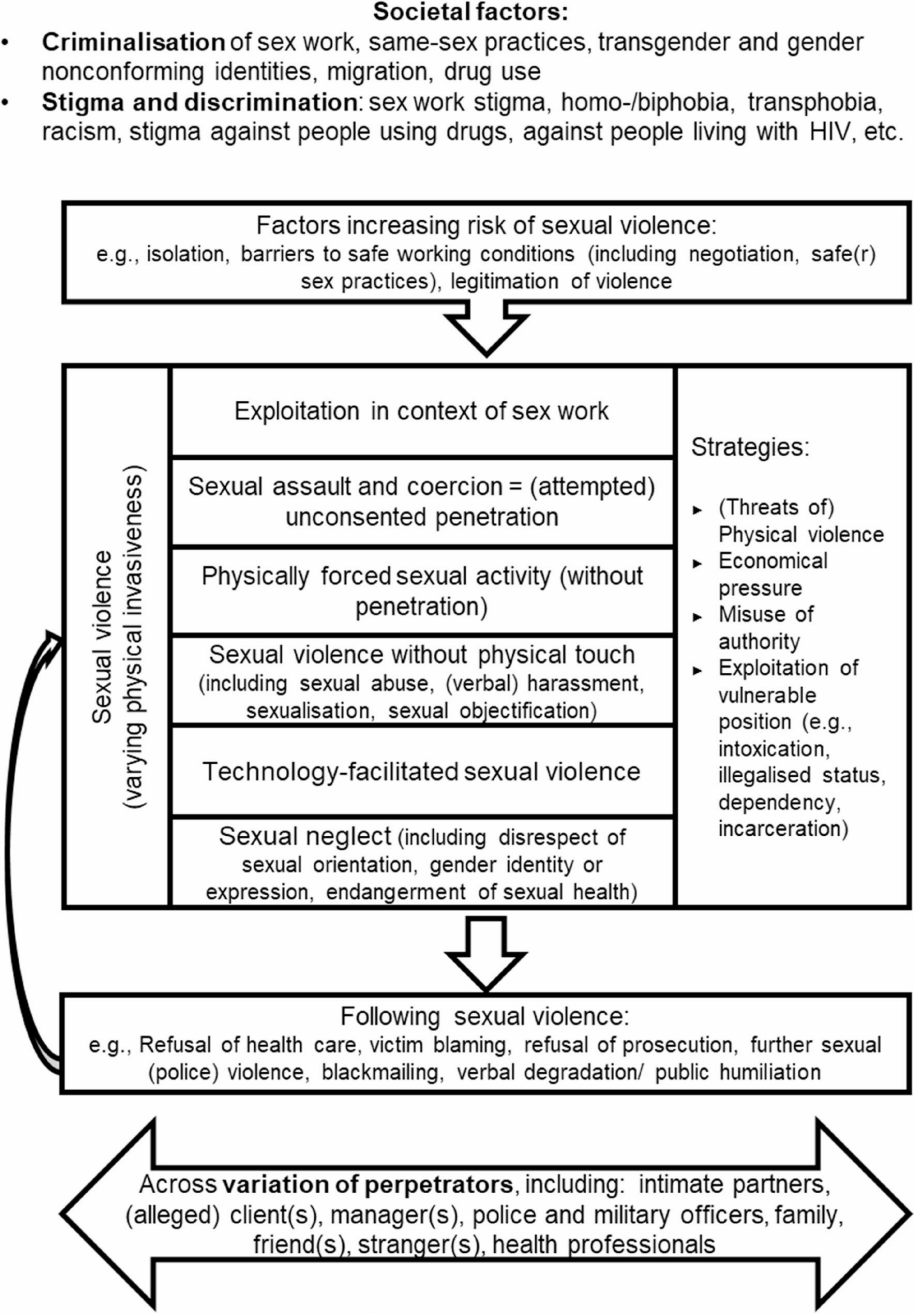
Qualitative summary of types of sexual violence

We identified six types of sexual violence, partially across contexts, as shown in Fig. 2. All included studies for each type including illustrations can be found in Additional file 4. Firstly, this included exploitation in context of sex work, here defined as forced sex exchange or forced entry into sex work. Even when focusing on sex work and not on sex trafficking, exploitation in context of sex work was in some studies reported as past experiences of sexual violence (e.g [35, 36]). Of a total of 592 codes given, we coded this theme 11-times (1.9%).

Secondly, the (attempted) unconsented penetration, by Baywell-Gray and colleagues [27] defined as sexual assault and coercion, included various subtypes of sexual violence such as with or without using physical violence or force (e.g [37–40]), , coerced penetration in exchange of freedom (e.g [36, 41]), – or in one case in exchange of being allowed to go to the bathroom [42] – after police arrest, coercion of condom-less sex using physical violence (e.g [43, 44]), or economical pressure (e.g [43, 45]), condom sabotage or stealthing (e.g [46, 47]), which was defined as “the removal of condoms during […] sex without the sex workers’ knowledge”, (p. 60) [48], longer sexual activity than agreed to (e.g [35, 43]), sex with more people than agreed to or gang rape (e.g [41, 49]), and refusal of payment after sexual service (e.g [50–52]). This category was predominantly described and coded *n* = 248 (41.7%) and referred to in the majority of quantitative studies.

Thirdly, physically forced sexual activity without penetration was reported as the following behaviours: non-consensual clothing removal (e.g [37, 51]), unwanted touching of own (e.g [42, 53]), or other’s body [54, 55], physical aggressiveness or violence during sexual intercourse (e.g [35, 56]), partly leading to severe sexual health conditions [38], unwanted genital examination or invasive body searches [42, 57, 58] as well as forced abortion [36, 59]. This theme was coded *n* = 33-times (5.6%) across all papers with qualitative results.

Fourthly, in-person sexual violence without physical touch (*n* = 80 codes, 13.5%) included sexual verbal harassment off- (e.g [39, 54]), and on-street (e.g [60, 61]), sexualized gestures [42, 58] or other types of invasiveness by e.g., stealing underwear while being searched by police [42, 58], sexual abusive behaviours [61, 62] to “keep partner in submissive position of power” (p. 323) [27], sexual objectification, dehumanization and fetishization (e.g [57, 63]), as well as voyeurism [53].

Fifthly, technology-facilitated sexual violence (*n* = 8 codes, 1.4%), defined as using digital technologies to sexually harm individuals, including intimate image abuse (e.g., non-consented recordings of sex tapes or other intimate images) [42, 58, 64–67], impersonation of police officers as clients [66] as well as unconsented sending of intimate images to sex workers was reported [68].

Sixthly, the last type relates to sexual violence broadly understood as the violation of human rights to sexual autonomy, bodily integrity and well-being as well as the endangerment of sexual health. We summarised those findings, that describe the sexual neglect of sex workers predominantly within the health and justice system. We included this, because of the high number of codes: *n* = 107 (18.1%). Following or not following completed forced sexual intercourse, sex workers, especially in criminalised contexts, are either personally refused (sexual) health care (e.g [38, 63]), or have reduced access to health care due to systematic restrictions (e.g [59, 69]), experience violations of privacy of confidentiality (e.g [38, 63]), live in fear of or experience hate crimes based on sexual orientation and/or gender identity (e.g [61, 70]), are forced to perform gendered behaviour (e.g., dancing in front of police) [54, 63], and further stigma and discrimination, including social exclusion (e.g [68, 71]).

Additional codes were given, that could not be integrated into this structure because of its complexity and/or mixture with other types of violence. This includes one study, describing sex workers being violently used of being a part of a sexualized ritual by a perpetrator pretending to be a client [45]. Sexual violence is often not separately experienced and sometimes combined with other types of violence (e.g., physical violence) (e.g [41, 72]). Furthermore, perpetrators use different strategies for sexual violations, including: (threats) of physical violence (e.g [43, 51, 73]), economical pressure (e.g [45–47]), misuse of authority (e.g [36, 54, 74]), or the exploitation of vulnerable positions because of e.g., intoxication, illegalised status, or being unhoused (e.g [43, 59, 67]).

We additionally identified that certain factors are reported that increase the risk of sexual violence (e.g., isolation, barriers to safe working conditions, legitimation of violence, reduced access to) [37, 52, 60, 67, 75, 76], and are relevant to health conditions that follow sexual violence (e.g., refusal of health care, victim blaming) (e.g [57, 63, 65, 67, 71, 74, 76–78]). Findings surrounding the experience of sexual violence highlight that societal factors including the criminalisation of sex work, same-sex practices, transgender identities, migration and drug use [37–39, 45, 60, 67, 71, 75], as well as stigma and discrimination including sex work stigma, homo- and biphobia, transphobia, racism, stigma against people using drugs as well as people living with HIV [37–39, 60, 63, 71, 77], are essential to understand the occurring of sexual violence against sex workers. While all types can be experienced in a variety of contexts and perpetrators, including intimate partners, (alleged) clients, managers, police and military officers, family, friends, strangers, and health professionals, sexual violence perpetrated at workplace (*n* = 238, 40.2%) and by uniformed (state) officers/ police (*n* = 146, 24.7%) were most frequently coded. Thereby, violence reported at workplace was mostly focused on clients or individuals posing as clients (*n* = 208, 35.1%).

### Results from quantitative studies

#### Obj. 2: Prevalences of sexual violence

An overview of all prevalences divided by time frame (lifetime, last year, last six months), context (overall, workplace, intimate partners, police, another, unspecified), type of sexual violence (sexual violence only or mixed types of violence) is shown in Table 2. Divided by WHO region is displayed in Additional file 5.

Data on sexual violence mixed with other types of violence included in all but three studies (of *n* = 145 effect sizes) physical violence. Most frequent combination was physical and sexual violence combined (*n* = 78), following physical, emotional/psychological, and sexual violence combined (*n* = 26) and physical, verbal, and sexual violence combined (*n* = 13). Other types of violence assessed with sexual violence were discrimination, stigma and economic violence. Subgroup analyses based on types of sex work could not be calculated due to lack of quantitative data on sex work besides of exchange of sex (such as dancing, solo content creation).

**Table 2:** Lifetime, past year and past six months prevalence of sexual violence

Context and type	Lifetime		Past year^b^		Past 6 months^c^	
	Prevalence (95% CI), %	*n*/*k* (I^2^)	Prevalence (95% CI), %	*n*/*k* (I^2^)	Prevalence (95% CI), %	*n*/*k* (I^2^)
Overall						
Sexual violence only	26.3 [22.0–31.0]	138/78 (96.6%)	17.7 [13.1–23.3]	60/33 (98.2%)	14.3 [11.0–18.3]	76/39 (97.3%)
Mixed types of violence^a^	52.1 [40.6–63.4]	51/28 (97.1%)	31.0 [18.2–47.5]	41/18 (98.8%)	30.4 [24.6–36.9]	56/33 (97%)
Sexual harassment	59.9 [49.1–69.8]	2/2 (-)	-	-	-	-
Formerly trafficked	11.4 [3.0–35.1]	7/7 (92.6%)	-	-	-	-
Workplace						
Sexual violence only	19.0 [12.6–27.6]	27/19 (96.3%)	20.7 [6.3–50.1]	9/7 (98.1%)	12.4 [8.3–18.2]	25/19 (97%)
Mixed types of violence^a^	42.2 [22.0–65.4]	15/11 (96.5%)	21.5 [4.1–63.5]	10/6 (99.3%)	30.1 [19.9–42.8]	19/19 (98.1%)
Intimate partner						
Sexual violence only	20.4 [13.1–30.4]	14/14 (93.6%)	20.7 [11.2–35.1]	11/9 (94.1%)	14.3 [8.2–23.7]	16/13 (93.6%)
Mixed types of violence^a^	50.5 [29.4–71.5]	11/11 (97.6%)	39.3 [23.4–57.9]	11/11 (98.5%)	26.3 [17.8–37.0]	15/15 (94.6%)
Police						
Sexual violence only	9.8 [3.8–22.6]	12/10 (94.7%)	5.4 [1.9–14.4]	6/6 (96.8%)	6.5 [3.0–13.6]	9/6 (97.1%)
Mixed types of violence^a^	29.8 [10.5–60.6]	6/6 (96.2%)	13.8 [3.7–40.2]	8/8 (98.8%)	14.0 [5.3–32.2]	5/4 (94.4%)
Another context						
Sexual violence only	14.2 [5.9–30.4]	16/11 (95.8%)	12.7 [0.4–83.3]	5/4 (98.8%)	12.9 [0.3–86.8]	3/3 (97.1%)
Mixed types of violence^a^	47.4 [8.5–89.7]	4/4 (97.3%)	10.2 [2.3–35.4]	5/5 (97.9%)	28.7 [6.4–70.2]	4/4 (96.1%)
Unspecified						
Sexual violence only	33.2 [28.3–38.5]	69/59 (96%)	19.0 [14.5–24.6]	29/24 (96.4%)	18.4 [12.6–26.1]	23/20 (98.2%)
Mixed types of violence^a^	59.2 [40.7–75.4]	15/14 (94%)	53.2 [19.1–84.6]	7/7 (97.8%)	37.4 [29.7–45.8]	13/12 (97%)

Note. *CI* Confidence Interval, *n* number of effect sizes, *k* number of studies, *I*^*2*^ Heterogeneity. “Sexual violence only” estimate = any sexual violence (broadly defined; see Methods). Harassment and mixed-violence outcomes were meta-analysed separately

I^2^ = -, meaning too little studies to measure heterogeneity properly

^a^ Example question: “Have you ever experienced sexual and/or physical violence?”

Prevalence of all “yes” answers

^b^ including timeframes up to 24 months and more than 6 months, predominantly 12 months

^c^ including timeframes less than 12 months, predominantly 6 months

#### Obj. 3: Associations with health

Prevalence of health-related symptoms was high, while I^2^ indicates a high heterogeneity across studies, and are shown in Additional file 6. Associations between sexual violence and mental health conditions, alcohol and other drug use, STI/HIV infection as well as reproductive health conditions are shown in Table 3.

**Table 3:** Associations between sexual violence and health conditions

Outcome	OR [95% CI]	*p*	*n*/*k* (I^2^)	aOR^a^ [95% CI]	*p*	*n*/*k* (I^2^)
Mental health conditions	1.85 [1.58–2.16]	**< **0.001	22/13 (76.2%)	2.03 [1.52–2.70]	**< **0.001	18/8 (81.8%)
Depression	1.80 [1.35–2.40]	0.001	10/9 (83%)	2.03 [1.23–3.35]	0.011	11/6 (88.6%)
PTS symptoms	2.09 [1.84–2.37]	< 0.001	4/2 (-)	1.70 [1.46–1.97]	0.014	2/1 (-)
Suicidality	1.91 [1.38–2.64]	0.003	7/5 (76.9%)	1.93 [1.74–2.14]	< 0.001	5/4 (-)
Alcohol and other drug use	1.95 [1.59–2.38]	**< **0.001	39/24 (90.5%)	1.69 [1.41–2.03]	**< **0.001	37/22 (90.6%)
Alcohol	1.75 [1.23–2.50]	0.004	16/13 (93.3%)	1.40 [0.99–1.99]	0.056	13/11 (89.9%)
Drug	1.94 [1.56–2.40]	< 0.001	23/20 (87.3%)	1.81 [1.48–2.20]	< 0.001	24/19 (86.4%)
STI/HIV infection	1.36 [1.11–1.66]	0.003	43/29 (75%)	1.53 [1.28–1.84]	**< **0.001	33/23 (81.5%)
HIV	1.27 [1.02–1.57]	0.036	28/22 (68.8%)	1.44 [1.18–1.75]	0.001	20/16 (53.1%)
STI	1.50 [1.09–2.07]	0.016	15/13 (73.3%)	1.79 [1.21–2.66]	0.007	13/12 (83.4%)
Reproductive health related conditions	1.76 **[**1.18–2.63]	0.013	8/8 (78.5%)	1.59 [1.32–1.92]	0.001	8/8 (24.5%)
Abortion	1.79 [0.98–3.25]	0.054	6/6 (84.2%)	1.55 [1.23–1.95]	0.004	6/6 (31.4%)

Note. *OR* Odds Ratio, *aOR* adjusted Odds Ratio, *CI* Confidence Interval, *n* number of effect sizes, *k* number of studies, *I*^*2*^ Heterogeneity, *PTS* Posttraumatic stress, *STI* sexually transmitted infections, *HIV* Human Immunodeficiency Virus

I^2^ = -, meaning too little studies to measure heterogeneity properly

^a^ Summary of all adjusted Odds Ratios and converted adjusted Prevalence/Risk Ratios. Controlled variables therefore vary by each study

#### Obj. 4: Legislative model as moderator

The subgroup analyses by legislative model were not significant. These included non-significant moderators for sexual violence lifetime prevalence (*F*(df1 = 4, df2 = 127) = 0.482, *p* = .746), in association between sexual violence and mental health symptoms (*F*(df1 = 3, df2 = 18) = 0.293, *p* = .830), in association between sexual violence and alcohol and other drug use (*F*(df1 = 3, df2 = 35) = 0.082, *p* = .969), in association between sexual violence and STI/HIV infection (*F*(df1 = 4, df2 = 38) = 0.613, *p* = .656), and in association between sexual violence and reproductive health outcomes (*F*(df1 = 2, df2 = 5) = 0.071, *p* = .933). Although the analyses did not indicate statistically significant moderation by legislative model, the distribution of studies across categories was highly uneven (criminalisation of purchase of sex: min. *k* = 0, max. *k* = 5; regulatory models: min. *k* = 0, max. *k* = 3; full decriminalisation: *k* = 0). Consequently, subgroup estimates primarily reflect comparisons between full criminalisation (min. *k* = 6, max. *k* = 81) and partial criminalisation (min. *k* = 1, max. *k* = 35), and residual heterogeneity remained substantial (Tables 2 and 3). Subgroup analyses accounting for further moderators are shown in Additional file 2, but substantial heterogeneity still remained.

## Discussion

Evidence synthesised in this review indicates that, across the included studies, approximately one in four sex workers has experienced sexual violence. However, this pooled estimate should be interpreted as an average across heterogeneous studies and contexts, rather than as a universally generalisable prevalence (see Limitations). Compared to non-sex-work-related prevalence rates, the WHO [33] reports that 6% of women^5^ experience sexual violence during their lifetime from non-intimate partners. However, the meta-analysis by Li and colleagues [79] states that one out of three women experience sexual violence in their lifetime, regardless of perpetrator. Compared to data by Deering and colleagues [4], that indicated experienced sexual violence from any (paying or non-paying) partner of 8 to 19% within the last year, this review presents a slightly higher prevalence of sexual violence between 13 and 23%, but included non-partners (e.g., police officers) as well. Differences between these estimates should be interpreted cautiously, as they may also reflect variation in definitions, sampling frames, and perpetrator/context classifications, in addition to potential true between-setting differences. Since data lacks both representation of diverse sociodemographic characteristics and consistent reporting of corresponding sample data (e.g., focus on cis women, street-based and in-person sex workers, aged 25 to 35, having primary education or less), these sexual violence prevalence estimates have limited external validity and should not be generalised to the broad community of sex workers. Key populations (e.g., transgender, nonbinary, male sex workers) and several types of sex work (e.g., escorting, professional BDSM, stripping, pornography, and online-based sex work) are underrepresented or absent in the included evidence (further details in Püffel et al., under review). The relatively similar 6-month and 12-month pooled estimates could be consistent with recurrent exposure in some settings. However, because these estimates derive from different studies and samples rather than within-person longitudinal follow-up, this pattern should be interpreted cautiously and cannot be taken as evidence of repeated victimisation at the individual level.

Qualitative studies add a narrative description to the violence experienced by sex workers. The meta-aggregation of this review showed that while different types of sexual violence with varying physical invasiveness and by various perpetrators, are experienced by sex workers, quantitative studies primarily focus on rape (unconsented penetration). This mismatch between reports in qualitative studies and selected focuses in quantitative studies indicates a potential bias in what is considered to be violent behaviour experienced by sex workers. Definitions used by community-led research, such as by Kloek and colleagues [51], give first insights into what should be included besides the normative understanding of rape in research practice:

“Sexual violence is any sexual act or attempted sexual act that the other person did not consent to. For any human being, part of sexual violence are sexual assault, rape and being coerced into performing sexual acts. For a sex worker, a particular aspect of sexual violence is being forced to provide unwanted sexual services, for instance incidents where you have agreed to provide specific services but eventually, without your consent, end up in a situation where you perform services you would normally refuse or that you are forced to perform. Examples are unwanted group sex, unwanted semen swallowing, unwanted stripping or wearing clothes or items you do not want to wear. Another special form of sexual violence is extortion for sex. You can be extorted by various means, for example with money, fines or being outed. Another aspect of sexual violence is sexual harassment. This can be physical, for instance when someone moves his [or their] hand through your hair or touches your breast without consent. It can also be verbal when someone makes unwanted sexual comments” (p. 18).

To capture structural, justice- and rights-based aspects linked to sexual violence, we propose sexual neglect as an interpretive, synthesis-derived concept (i.e., not directly measured in the included primary studies). We use sexual neglect to denote omissions and systemic failures that undermine sex workers’ sexual autonomy and bodily integrity (e.g., denial of care, protection, and free expression of sexual orientation and gender identity). Thereby, we refer to sexual justice, which includes the concept of dignity, alongside other principles such as intersectionality, citizenship and belonging, as well as autonomy, consent and diversity. The WAS calls “for respect for each person’s integrity, and the protection of all people from degrading treatment. [Dignity] affirms not only the right to healthcare, but also the right to consent, respect, and confidentiality, among other rights.” (p. 9) [7]. In this framing, qualitative reports of sex work stigma and secondary victimisation [37–39, 60, 63, 71, 77], are interpreted as potential mechanisms through which intersectional stigma may undermine sexual autonomy, integrity as well as sexual health and shape post-assault experiences [3, 6, 15]. Future work should operationalise and validate the construct of sexual neglect (including boundaries to related concepts such as structural violence and stigma) before it is used for prevalence estimation or causal modelling. Additionally, the interplay between sexual violence and sex work stigma should be explored. Further research should investigate how sex workers define sexual violence and analyse differences between sex workers, as well as intrapersonal changes in these definitions across the life course.

### Health consequences within a restricted conceptual lens

Interestingly, though we did find significant associations between sexual violence and mental and sexual health, the odds ratios for mental health were not as high as in general populations [9]. Potential explanations may be rooted in the specific health outcomes prioritised in sex work research. Specifically, the disproportionate focus on substance uses and STI/HIV often reinforces stigmatizing myths of sex workers as vectors of infection or addiction. Researchers should critically reflect on how these narrow research priorities may inadvertently perpetuate social marginalization [22]. Conversely, significant mental health conditions that are more strongly associated with sexual assault, such as post-traumatic stress [9] seem more often neglected. As a result, focusing primarily on substance uses likely attenuates the observed associations, producing smaller-than-expected odds ratios. Regarding sexual health, relevant conditions following sexual violence such as sexual functioning and sexual well-being [80, 81] were completely missing and should be investigated in future research. Additionally, potential resilience factors and coping strategies used by sex workers need to be explored.

The association between sexual violence and alcohol or other drug use may be consistent with self-medication hypotheses [9]. This interpretation was consistent with stakeholder discussions, who provided context regarding substance use as a means of mitigating violence-related distress. Especially considering high barriers to access to health for sex workers [13, 14, 63, 82], substance use may function as a relevant strategy of handling distress following sexual violence for some individuals. However, given the predominance of cross-sectional data, we cannot establish temporality. Alcohol and other drug use alongside other health conditions may precede, follow, or co-occur with sexual violence exposure, and both may be shaped by shared structural determinants (e.g., stigma, criminalisation, financial stress) [3, 9, 16, 83]. Future research should investigate a potential moderating effect of barriers to health care between sexual violence and health as well as strategies to minimise barriers to health care.

### Mixed and missing evidence on legislation as an impacting factor on sexual violence

Finally, the legislative model was not statistically related to sexual violence, nor did it moderate the association between sexual violence and health conditions in quantitative analyses. However, this should not be interpreted as evidence that legislation is unrelated to sexual violence risk or its health correlates. Rather, it seems that the moderation analyses were constrained by substantial imbalance and absence across legislative categories (including no eligible studies under full decriminalisation and only sparse evidence for criminalisation of purchase of sex and regulatory models). Consequently, the available quantitative evidence largely may reflect comparisons between full and partial criminalisation, and therefore, cannot be generalised to other legislative approaches. In addition, our coding operationalised legislative context as “law in books” rather than enforcement (see Limitations) [29]. Thus, qualitative evidence in this review primarily informs hypotheses about mechanisms that require stronger comparative quantitative testing.

Qualitative results suggest that criminalisation-related legal and policing environments may increase vulnerability to sexual violence. As supported by the meta-analysis by Platt and colleagues [16], criminalisation in any form limits sex workers ability for safe negotiations and practices. For example, with criminalisation, sex workers are forced to move their work location to places that are less visible, have less possibilities to network with other sex workers for safety measures, and may increase vulnerability to sexual violence [60, 65, 76]. This is consistent with a scoping review on prevention of violence against sex workers [84]. Especially in context of sexual police violence, exploiting vulnerability caused by criminalisation is used as a strategy to enforce sexual violence by arresting first and then extorting “sex”^6^ in exchange for freedom (e.g [36, 41]). Furthermore, our meta-aggregation suggests that not only the criminalisation of sex work, but also of same-sex practices, trans identities, substance use, and migration can increase the vulnerability to sexual violence [37–39, 45, 59, 60, 67, 71, 75]. As proposed by international organisations such as Amnesty International and the WHO [17, 19], these qualitative results underline the criminalisation of sex work as a human rights violation. This crucial aspect suggests that the legislative model is a central variable for sexual violence and health conditions [6]. It is therefore urgently necessary to include this variable more regularly in quantitative research and specifically collect data on countries with the model of criminalisation of purchase of sex, regulatory models, and full decriminalisation.

### Limitations

There are several limiting factors for this systematic review, meta-aggregation and meta-analysis. First, across meta-analytical analyses, studies displayed a high heterogeneity (mostly *I*^*2*^ > 90% for prevalence estimates) and could not be meaningfully reduced through subgroup analyses. This indicates substantial between-study variability that likely reflects true differences across contexts (e.g., law enforcement, work settings, intersectional stigma) [85, 86] as well as methodological diversity (e.g., sampling, operationalisation of sexual violence, data collection methods). Accordingly, pooled prevalence estimates should be interpreted as averages across heterogeneous studies rather than as a single, generalisable global rate. Relatedly, population representativeness in general is difficult to achieve in sex work research, since both stigma and criminalisation decrease sex workers’ safety, constrain disclosure, and limit study participation. Community-led or -assisted research may mitigate some of these limitations and improve validity of collected data [87]. Additionally, methodological differences, e.g., in the measurement of sexual violence, could account for the high heterogeneity.

Secondly, definitions on both sexual violence and sex work were restricted or missing in a substantial proportion of the included studies. This limits comparability and constrains generalisation of prevalence estimates, as sexual violence can – as displayed above – occur in various types, and, sex work encompasses diverse services, considering that sex workers might provide multiple services (e.g., both in-person and online sex work).

Thirdly, the interpretation of the odds ratios describing the association between sexual violence and health conditions is limited by the restricted diversity of included health conditions, as well as by the high proportion of cross-sectional data. Associations between sexual violence and health conditions do not imply causation, therefore can be due to bidirectional or confounded relationships. Furthermore, the study quality assessment was selected to be appropriate for cross-sectional studies. Consequently, a rating of high study quality refers to methodological quality within the inherent limitations of cross-sectional designs and does not allow conclusions regarding causality.

Fourthly, too few studies from legislative models criminalizing the purchase of sex and regulatory models were included, and no studies were available for full decriminalisation, limiting the extent to which statistical conclusions can be drawn regarding moderation of sexual violence by legislative model. Further, our implementation of the legislative models operationalised “law in book” rather than enforcement (“law in action”) [29]. Trying to fit the regulation of sex work into discrete categories is challenging given the “lack of a coherent system of prostitution policy classification” (p. 1) [88]. Therefore, the applied categorisations may not fully capture cross-national and subnational variation in implementation. We adopted a “law in books” approach [29], as a “law in action” approach would have required systematic assessment of enforcement across 98 countries included. Future research should incorporate indicators of enforcement and policing practices, and account for within-country legal heterogeneity. In addition to the laws that regulate sex work, some other laws might affect the criminalisation of sex workers, such as the criminalisation of same-sex activities, migration or substance use, though these were not considered due to feasibility.

Lastly, while including non-English publications reduced bias, the machine translations of those publications were not verified by independent native speakers due to resource constraints. Some risk of translation-related information bias (e.g., misclassification due to misinterpretation of context-specific terminology) remains.

### Implications

Implications should be interpreted in light of the overrepresentation of cis women and in-person sex work in the included studies. With high prevalences of unconsented penetration in the included samples of sex workers, the prevention of sexual violence is essential. We understand sexual violence as part of a structural phenomenon [89] that requires structural change to prevent. In context of sex work, decriminalisation has been proposed and is supported by prior syntheses as a potentially important structural approach to reducing sex workers’ risk of violence [16]. Further, destigmatisation efforts are essential [3, 22]. Moreover, while preventing sexual violence could plausibly be beneficial for sex workers’ health, additional improvement of access to health is essential [13, 14, 82].

Future research could focus on high quality data collection considering the heterogeneity of sex workers [85, 86] – especially in countries where sex work is decriminalised and therefore sex workers are safer to participate in sensitive research projects. Further, high-quality evaluations of implementations of laws are needed to inform evidence-based policies. Research on destigmatising processes could inform about needed societal intervention, including interventions for institutions (e.g., to prevent sexual police violence). Lastly, community-led research could improve reaching an often hard-to-reach population, improving effectivity of interventions and increase validity of data [87, 90].

## Conclusion

This multi-method systematic review synthesised evidence across divers regions, integrating peer-reviewed publications with community-led grey literature. By incorporating these sources, the review mitigates publication bias and captures critical insights frequently absent from traditional academic databases. We summarised reported types and prevalences of sexual violence against sex workers (predominantly cisgender women in in-person settings) and present pooled estimates as averages across heterogeneous contexts. Given substantial heterogeneity and variability in how sexual violence and sex work were operationalised, these estimates should be interpreted cautiously and not as universally generalisable global rates. We also examined potential associations with mental and sexual health conditions, while considering varying legislative models. Overall, the findings indicate that sex workers experience a wide range of sexual violence. The definition of sexual violence is a delicate topic, especially when done without the consideration of survivors of sexual violence themselves. Recognising sexual violence against sex workers as a human rights issue is crucial. However, as three out of four sex workers do not experience sexual violence in their lifetime, it should not be viewed as an inherent aspect of sex work, contrary to certain political narratives [91], but rather be contextualised as aspects of structural violence and oppression against sex workers [3, 6, 89]. The significant association of sexual violence with various health conditions (mental health, alcohol and other drug use, sexually transmitted infections, reproductive health) support the inclusion of sexual violence in health interventions, while acknowledging that the evidence base is predominantly cross-sectional. While the quantitative evidence on legislation is inconclusive, qualitative data suggests that criminalisation in varied forms may increase vulnerability to sexual violence and comprehensive quantitative research is needed to explore its full impact. This review might contribute to the destigmatisation of both sex work and sexual violence by providing combined narrative and statistic data. Further efforts on destigmatisation are essential to address social inequities and health disparities among sex workers.

## Supplementary Information

Additional file 1. Search string.

Additional file 2. Additional analyses to detect bias and causes of heterogeneity (Subgroup analyses, funnelplots, Eggers’ test).

Additional file 3. Overview of included studies.

Additional file 4. Details on qualitative results.

Additional file 5. Sexual violence prevalences by WHO region.

Additional file 6. Prevalences of health-related symptoms.

## Data Availability

All quantitative data generated or analysed during this study are included in this published article and its supplementary information files. The qualitative datasets used and/or analysed during the current study are available from the corresponding author on reasonable request.

## Footnotes

1 By type of sexual violence, we refer to different perpetrating behaviors such as coerced penetration, unconsented touching, sexualized verbal harassment.

2 By type of sex work, we refer to services provided by sex workers (e.g., penetrative sex, erotic dance/stripping, adult content) and/or location (such as mainly working indoors in brothels, in hotels/bars, or at home, mainly working outdoors, or mainly working in online contexts). Nevertheless, these categories are not mutually exclusive and sex workers might provide multiple types of services in varying locations.

3 By context of sexual violence, we refer to the relationship to the perpetrator and/or location (e.g., sexual violence happened while working, by police, by intimate partners).

4 We define the legislative models as laws in book that are “used to manage, control, or regulate sex work” (p. 4) [16] .

5 Since the majority of included study participants were women or marked as female, this reference data was provided. Beyond the data on sexual violence from non-intimate partners, the WHO only reported prevalence data on sexual and/or physical violence perpetrated by intimate partners.

6 Because consent is absent, the act cannot be characterized as sex and must be understood as sexual violence.

## Abbreviations

BesD: Berufsverband für erotische und sexuelle Dienstleitungen [Professional Association for Erotic and Sexual Service Providers
ESWA: European Sex Workers‘ Rights Alliance
ESWORN: European Sex Work Research Network
HIV: Human Immunodeficiency Virus
JBI: Joanna Briggs Institute
NSWP: Global Network of Sex Work Projects
STI: sexually transmitted infections
SWAN: Sex Workers’ Advocacy Network
SWP: The Sex Workers Project
WAS: The World Association for Sexual Health
WHO: World Health Organization
