# Supplementary material for: Systematic review of sexual violence against sex workers: implications for mental and sexual health
Source: BMC Public Health. 2026 Jun 30;26:2126. doi: 10.1186/s12889-026-28204-4 (PMC13360242; doi:10.1186/s12889-026-28204-4)
Supplement: Supplementary file 1 — Additional file 1. Search string. [file 12889_2026_28204_MOESM1_ESM.docx]

**Systematic review of sexual violence against sex workers: Implications for mental and sexual health**

**Additional file 1**

Marie Püffel1, İsmail Orbay2*, Ira Salo3*, Henriette Berg1*, Lea Hasanagic1*, Elisa Ruiz Burga4, Thérèse Bernier5, Nina Heinrichs1

1Bielefeld University | Department of Psychology | Bielefeld | Germany

2Protestant University of Applied Sciences Berlin | Department of Social Work | Berlin | Germany

3University of Turku | Faculty of Law | Turku | Finland

4University College London | Institute of Global Health | London | United Kingdom

5George Brown Polytechnic | Faculty of Applied Science, Construction and Engineering Technology | Toronto | Canada

* Authors had same amount of contribution to paper

**Table A Example search strategy for MEDLINE, reached via Ovid**

| Concept | Search string |
| --- | --- |
| Sex work | (("sex work$" OR prostitut$ OR "sex industry" OR "sex trade" OR “transactional sex” OR “commercial sex” OR “sell$ sex” OR “sold sex” OR exchang$ sex” OR “sex exchang$” OR escort$ OR "porn act$" OR camming OR camgirl$ OR "cam girl$" OR ("sex work$" adj5 online) OR stripping OR stripper$ OR "exotic danc$" OR “erotic danc$” OR (“massage” adj5 (erotic OR parlor OR salon)) OR dominatrix OR “pro-dom$” OR "sugar babe$" OR "sexual bodywork$" OR “content creator$” OR “phone sex operator$” OR “sexual assist$”).ti,ab,kw.)  AND |
| Sexual violence (general) | ("sexual violence" OR "sexual harassment" OR rape OR "sexual assault" OR "unwanted sexual experience$" OR "sexual trauma" OR ("intimate partner violence" adj5 sexual) OR ("domestic violence" adj5 sexual) OR "sexual abuse" OR ("police violence" adj5 sexual) OR “forced nudity” OR “cavity search” OR “body-search$” OR stealthing OR “covert condom removal” OR “forced condom removal”  OR |
| Sexual violence (technology-facilitated) | (("sexual violence" OR "sexual harassment" OR "sexual assault$" OR "sexual abuse" OR "dating abuse" OR "intimate partner") adj5 ("technology-facilitated" OR online OR digital OR image-based OR cyber$)) OR “revenge-porn$”  OR |
| CM and neglect | maltreatment OR neglect).ti,ab,kw. |
