# Supplementary material for: Systematic review of sexual violence against sex workers: implications for mental and sexual health
Source: BMC Public Health. 2026 Jun 30;26:2126. doi: 10.1186/s12889-026-28204-4 (PMC13360242; doi:10.1186/s12889-026-28204-4)

**Systematic review of sexual violence against sex workers: Implications for mental and sexual health**

**Additional file 2**

Marie Püffel1, İsmail Orbay2*, Ira Salo3*, Henriette Berg1*, Lea Hasanagic1*, Elisa Ruiz Burga4, Thérèse Bernier5, Nina Heinrichs1

1Bielefeld University | Department of Psychology | Bielefeld | Germany

2Protestant University of Applied Sciences Berlin | Department of Social Work | Berlin | Germany

3University of Turku | Faculty of Law | Turku | Finland

4University College London | Institute of Global Health | London | United Kingdom

5George Brown Polytechnic | Faculty of Applied Science, Construction and Engineering Technology | Toronto | Canada

* Authors had same amount of contribution to paper

**Table A Overview of subgroup analyses and potential significant moderators**

| Outcome | Moderator | Estimate | Lower CI | Upper CI | *p* | *k* |
| --- | --- | --- | --- | --- | --- | --- |
| **Sexual violence lifetime prevalence**  Reference categories:  Legislative model: Not specified,  Context: Unspecified,  Quality assessment: Good | Intercept | 0.335 | 0.179 | 0.537 | 0.106 | 132 |
|  | Legislative model: Full criminalisation | 0.546 | 0.352 | 0.727 | 0.646 | 132 |
|  | Legislative model: Criminalisation of purchase of sex | 0.658 | 0.176 | 0.946 | 0.556 | 132 |
|  | Legislative model: Partial criminalisation | 0.480 | 0.285 | 0.680 | 0.848 | 132 |
|  | Legislative model: Regulatory models | 0.421 | 0.144 | 0.759 | 0.669 | 132 |
|  | **Context of sexual violence: Workplace** | 0.302 | 0.206 | 0.419 | **0.002** | 132 |
|  | **Context of sexual violence: Partner** | 0.323 | 0.210 | 0.463 | **0.015** | 132 |
|  | **Context of sexual violence: Police** | 0.149 | 0.080 | 0.260 | **0.000** | 132 |
|  | **Context of sexual violence: Other** | 0.249 | 0.155 | 0.375 | **0.000** | 132 |
|  | Quality assessment: Fair | 0.535 | 0.417 | 0.648 | 0.562 | 132 |
|  | Quality assessment: Poor (if poor, please state why) | 0.506 | 0.329 | 0.682 | 0.946 | 132 |
| **Mental health conditions**  Reference categories:  Legislative model: Not specified,  Context of sexual violence: Unspecified,  Outcome: Depressive symptoms,  Sexual violence assessed only,  Quality assessment: Good | Intercept | 3.270 | 0.373 | 28.677 | 0.244 | 21 |
|  | Legislative model: Full criminalisation | 0.741 | 0.076 | 7.247 | 0.770 | 21 |
|  | Legislative model: Criminalisation of purchase of sex | 0.434 | 0.035 | 5.338 | 0.465 | 21 |
|  | Legislative model: Partial criminalisation | 0.476 | 0.050 | 4.566 | 0.471 | 21 |
|  | Context of sexual violence: Workplace | 0.750 | 0.408 | 1.378 | 0.307 | 21 |
|  | Context of sexual violence: Partner | 0.722 | 0.444 | 1.172 | 0.160 | 21 |
|  | **Context of sexual violence: Police** | **0.409** | **0.222** | **0.753** | **0.010** | **21** |
|  | Context of sexual violence: Other | 0.869 | 0.536 | 1.408 | 0.521 | 21 |
|  | Mixed types of violence | 0.845 | 0.324 | 2.200 | 0.695 | 21 |
|  | Outcome: Other | 0.970 | 0.179 | 5.257 | 0.968 | 21 |
|  | **Outcome: PTSD** | **1.551** | **1.188** | **2.026** | **0.005** | **21** |
|  | **Outcome: Suicidality** | **1.638** | **1.145** | **2.344** | **0.013** | **21** |
|  | Quality assessment: Fair | 1.261 | 0.361 | 4.407 | 0.680 | 21 |
| **Alcohol and other drug use**  Reference categories:  Legislative model: Not specified,  Context of sexual violence: Unspecified,  Outcome: Alcohol use,  Sexual violence assessed only,  Quality assessment: Good | Intercept | 1.536 | 0.369 | 6.393 | 0.541 | 37 |
|  | Legislative model: Full criminalisation | 1.186 | 0.314 | 4.479 | 0.794 | 37 |
|  | Legislative model: Criminalisation of purchase of sex | 0.854 | 0.218 | 3.345 | 0.814 | 37 |
|  | Legislative model: Partial criminalisation | 1.216 | 0.286 | 5.162 | 0.783 | 37 |
|  | Context of sexual violence: Workplace | 0.971 | 0.472 | 1.994 | 0.933 | 37 |
|  | Context of sexual violence: Partner | 0.978 | 0.541 | 1.770 | 0.940 | 37 |
|  | Context of sexual violence: Police | 1.124 | 0.489 | 2.583 | 0.774 | 37 |
|  | Context of sexual violence: Other | 0.718 | 0.398 | 1.296 | 0.260 | 37 |
|  | Mixed types of violence | 1.114 | 0.600 | 2.067 | 0.724 | 37 |
|  | **Outcome: Drug use** | **1.321** | **1.004** | **1.738** | **0.047** | **37** |
|  | Quality assessment: Fair | 0.951 | 0.514 | 1.762 | 0.870 | 37 |
| **STI/HIV infection**  Reference categories:  Legislative model: Not specified,  Context of sexual violence: Unspecified,  Outcome: HIV,  Sexual violence assessed only,  Quality assessment: Good | Intercept | 1.301 | 0.615 | 2.753 | 0.478 | 40 |
|  | Legislative model: Full criminalisation | 0.917 | 0.495 | 1.698 | 0.775 | 40 |
|  | Legislative model: Criminalisation of purchase of sex | 0.698 | 0.137 | 3.561 | 0.655 | 40 |
|  | Legislative model: Partial criminalisation | 0.849 | 0.503 | 1.432 | 0.526 | 40 |
|  | Legislative model: Regulatory models | 0.567 | 0.161 | 2.000 | 0.364 | 40 |
|  | Context of sexual violence: Workplace | 0.853 | 0.567 | 1.281 | 0.430 | 40 |
|  | Context of sexual violence: Partner | 0.697 | 0.410 | 1.185 | 0.175 | 40 |
|  | Context of sexual violence: Police | 1.086 | 0.687 | 1.718 | 0.715 | 40 |
|  | **Context of sexual violence: Other** | **3.174** | **1.397** | **7.212** | **0.007** | **40** |
|  | Mixed types of violence | 0.955 | 0.661 | 1.380 | 0.801 | 40 |
|  | Outcome: Sexually transmitted infection | 0.953 | 0.686 | 1.323 | 0.765 | 40 |
|  | **Quality assessment: Fair** | **1.841** | **1.256** | **2.698** | **0.003** | **40** |
| **Reproductive health related conditions**  Reference categories:  Legislative model: Not specified,  Context of sexual violence: Unspecified,  Sexual violence assessed only | Intercept | 1.900 | 0.144 | 25.034 | 0.396 | 7 |
|  | Legislative model: Full criminalisation | 0.579 | 0.025 | 13.331 | 0.532 | 7 |
|  | Legislative model: Partial criminalisation | 1.004 | 0.027 | 37.358 | 0.996 | 7 |
|  | Context of sexual violence: Partner | 0.847 | 0.070 | 10.230 | 0.801 | 7 |
|  | Mixed types of violence | 4.299 | 0.444 | 41.629 | 0.110 | 7 |

Note: **Bold** = p-value significant (< .05), CI = Confidence interval, k = number of studies, HIV = Human Immunodeficiency Virus

**Table B Egger’s test to detect asymmetry and publication biases**

| Outcome | Bias estimate | *SE* | *t* | *df* | *p* |
| --- | --- | --- | --- | --- | --- |
| Sexual violence proportion - lifetime | -2.15 | 1.14 | -1.89 | 196 | .060 |
| Sexual violence proportion - year | -2.65 | 1.96 | -1.35 | 99 | .180 |
| Sexual violence proportion - months | -5.37 | 1.39 | -3.86 | **130** | **< .001** |
| Mental health conditions | 0.51 | 1.06 | 0.48 | 20 | .635 |
| Alcohol and other drug use | 3.62 | 0.95 | 3.81 | **37** | **< .001** |
| STI/HIV infection | -0.38 | 0.43 | -0.88 | 41 | .384 |
| Reproductive health related conditions | Too little studies (k = 8) | | | | |

Note: These tests were calculated without using a multi-level meta-analysis. Each effect size was used individually without grouping by study.

# Figures

##
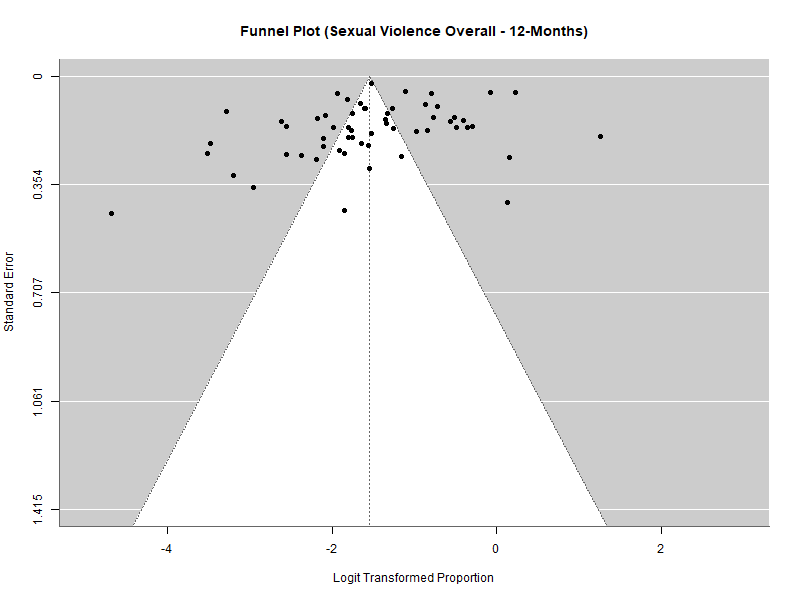

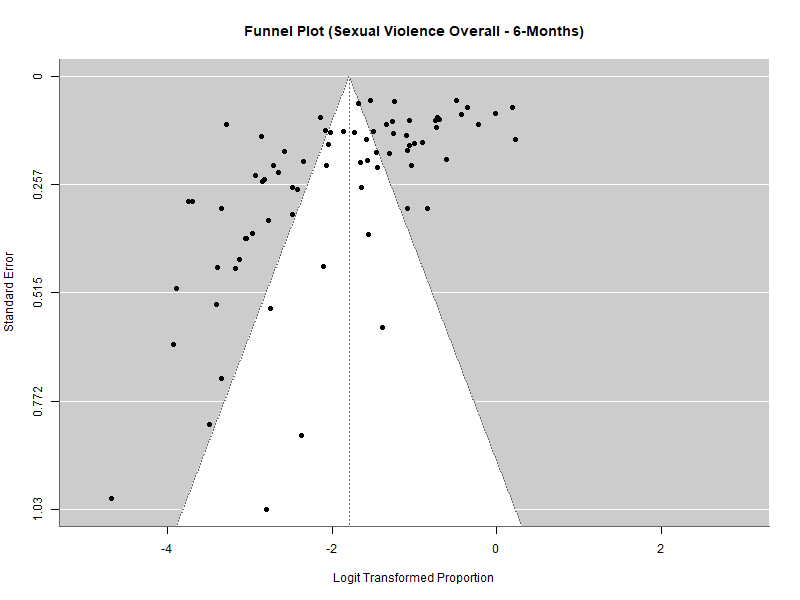
Figure A. Funnel plots sexual violence over all contexts


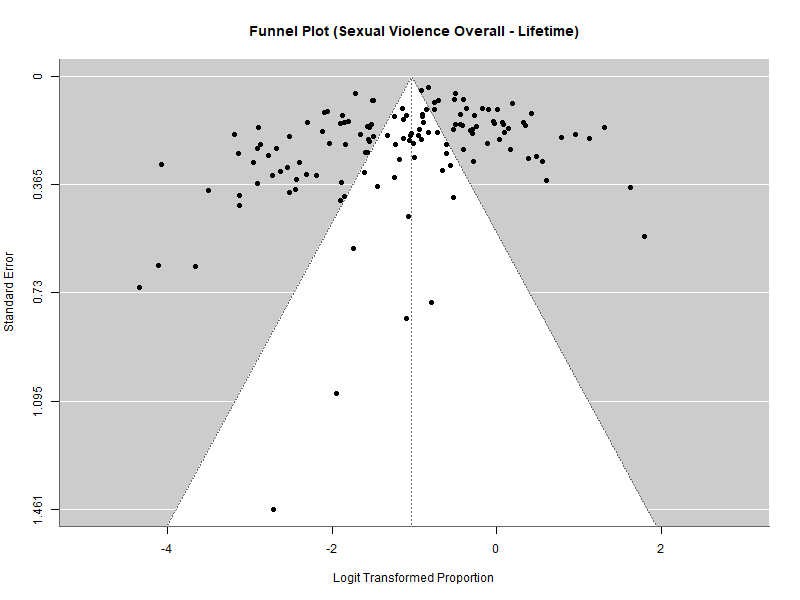


##
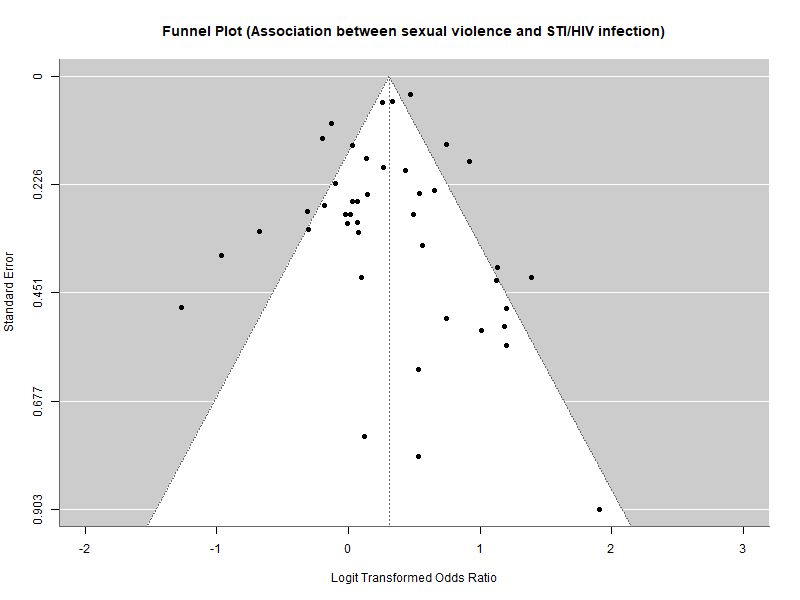

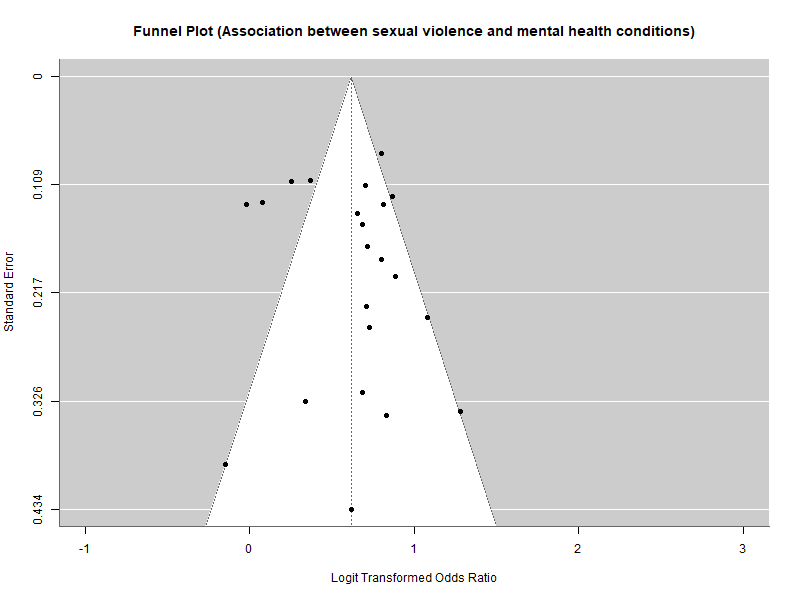
Figure B. Funnel plots health conditions


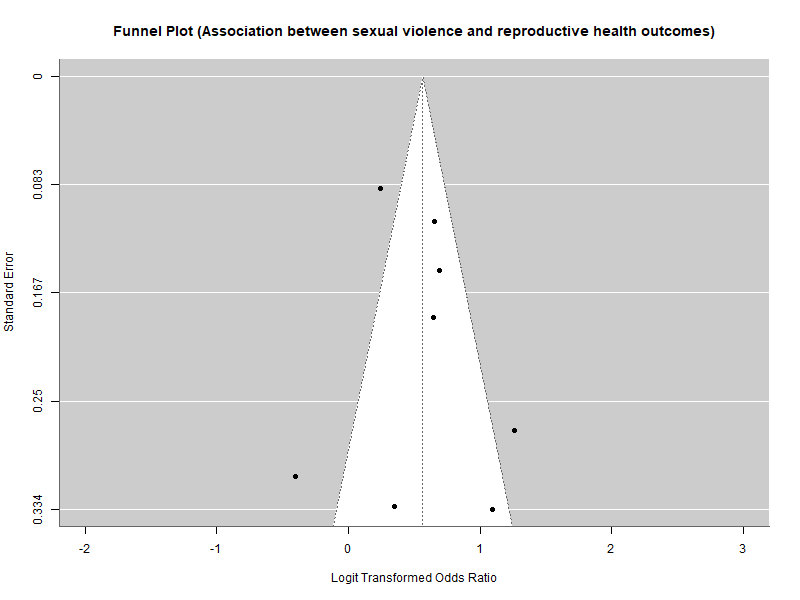

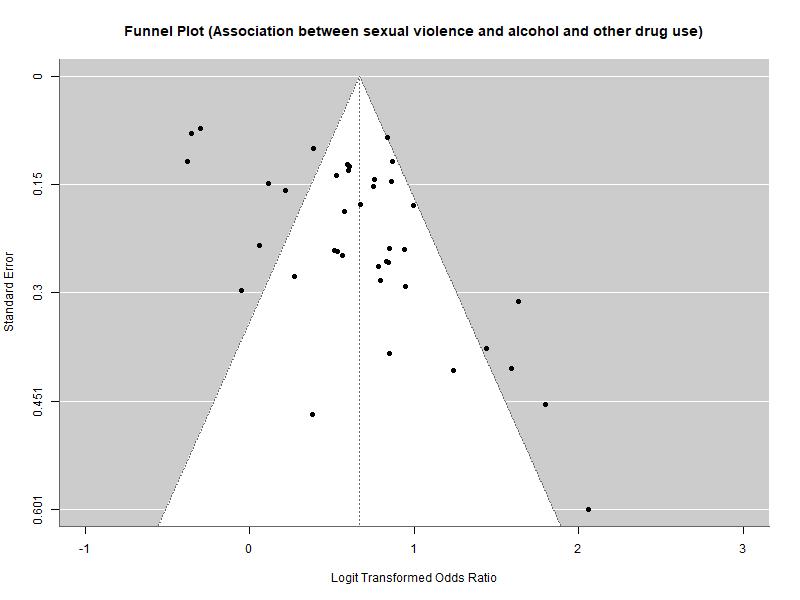

Supplement: Supplementary file 2 — Additional file 2. Additional analyses to detect bias and causes of heterogeneity (Subgroup analyses, funnelplots, Eggers’ test). [file 12889_2026_28204_MOESM2_ESM.docx]
