# Supplementary material for: Systematic review of sexual violence against sex workers: implications for mental and sexual health
Source: BMC Public Health. 2026 Jun 30;26:2126. doi: 10.1186/s12889-026-28204-4 (PMC13360242; doi:10.1186/s12889-026-28204-4)
Supplement: Supplementary file 3 — Additional file 3. Overview of included studies. [file 12889_2026_28204_MOESM3_ESM.docx]

**Systematic review of sexual violence against sex workers: Implications for mental and sexual health**

**Additional file 3**

Marie Püffel1, İsmail Orbay2*, Ira Salo3*, Henriette Berg1*, Lea Hasanagic1*, Elisa Ruiz Burga4, Thérèse Bernier5, Nina Heinrichs1

1Bielefeld University | Department of Psychology | Bielefeld | Germany

2Protestant University of Applied Sciences Berlin | Department of Social Work | Berlin | Germany

3University of Turku | Faculty of Law | Turku | Finland

4University College London | Institute of Global Health | London | United Kingdom

5George Brown Polytechnic | Faculty of Applied Science, Construction and Engineering Technology | Toronto | Canada

* Authors had same amount of contribution to paper

Content

[Studies included in meta-analysis 3](#_Toc226474858)

[Table A1: Included quantitative studies from countries with full criminalisation 3](#_Toc226474859)

[Table A2: Included quantitative studies from countries with criminalisation of purchase of sex 60](#_Toc226474860)

[Table A3: Included quantitative studies from countries with partial criminalisation 65](#_Toc226474861)

[Table A4: Included quantitative studies from countries with regulatory models 92](#_Toc226474862)

[Table A5: Included quantitative studies from countries with not specified legislative models 95](#_Toc226474863)

[Studies included in meta-aggregation 103](#_Toc226474864)

[Table B1: Included qualitative studies from countries with full criminalisation 103](#_Toc226474865)

[Table B2: Included qualitative studies from countries with criminalisation of purchase of sex 111](#_Toc226474866)

[Table B3: Included qualitative studies from countries with partial criminalisation 114](#_Toc226474867)

[Table B4: Included qualitative studies from countries with regulatory models 119](#_Toc226474868)

[Table B5: Included qualitative studies from countries with full decriminalisation 120](#_Toc226474869)

[Table B6: Included qualitative studies from countries with not specified legislative models 121](#_Toc226474870)

[References 125](#_Toc226474871)

Studies included in meta-analysis

## Table A1: Included quantitative studies from countries with full criminalisation

| First Author, year  (Study quality)  [Publications used] | Targeted population | Study type | Region / country | Data collection time period | N | Sexual violence prevalences  % [95 %CI] | Mental health outcomes  OR/PR/RR  [95 % CI] | Sexual health outcomes  OR/PR/RR  [95 % CI] |
| --- | --- | --- | --- | --- | --- | --- | --- | --- |
| Ayamah et al., 2023 (Fair)  [1] | Sex workers addressed only;  Inclusion criteria: Received money in the last 6 months from someone other than an established partner in exchange for sexual services (whether or not self-identified as ‘sex work’),  aged ≥16 years.  Exclusion criteria: None reported. | Cross sectional study | LMICs: African Region: Ghana | 2015 - 2015 | 4279 | Past year prevalence: Sexual violence only: Unspecified: 24.7 [23.5 - 26.0];  Police: 3.6 [3.0 - 4.2];  6-months prevalence: Sexual violence only: Police: 3.6 [3.1 - 4.2];  Unspecified: 17.8 [16.6 - 19.0] | Alcohol use:  OR = 0.7 [0.6 - 0.8]; aOR = 0.7 [0.6 - 0.9];  Drug use: OR = 0.7 [0.6 - 0.8]; aOR = 0.8 [0.7 - 0.9] | - |
| Baral et al., 2014 (Fair)  [2] | Sex workers addressed only;  Inclusion criteria: Women who reported selling sex for money in the previous 12 months,  adults,  located in Swaziland.  Exclusion criteria: None reported. | Cross sectional study | LMICs: African Region: Swaziland | August 2011 - October 2011 | 317 | Lifetime prevalence: Sexual violence only: Unspecified: 39.2 [33.8 - 44.6]; 7.3 [4.4 - 10.2] | - | - |
| Beattie et al., 2024 (Good)  [3–9] | Sex workers addressed only;  Inclusion criteria: Women aged 18–45 years,  not pregnant or breastfeeding,  without chronic illnesses  affecting immunology (e.g., diabetes, rheumatoid arthritis, asthma, TB, recent cervical therapy),  and had attended a SWOP clinic in Nairobi in the past 12 months.  Exclusion criteria: None reported. | Cross sectional study | LMICs: African Region: Kenya | June 2019 - December 2019 | 1003 | 6-months prevalence: Sexual violence only: Unspecified: 49.8 [46.7 - 52.8];  2.3 [1.4 - 3.2];  39.6 [36.6 - 42.6];  Partner: 21.9 [19.4 - 24.5];  Lifetime prevalence: Sexual violence only: Unspecified: 9.1 [7.3 - 10.9]; Police: 13.3 [11.2 - 15.4];  6-months prevalence: Mixed types of violence: Unspecified: 65.6 [62.7 - 68.5]; 54.4 [51.4 - 57.5]  Partner: 33.8 [30.9 - 36.7];  Lifetime prevalence: Mixed types of violence:  Unspecified: 68.6 [65.7 - 71.5];  Partner: 55.8 [52.8 - 58.9]; | Alcohol use: OR = 5.1 [2.8 - 9.4]; OR = 2.1 [1.6 - 2.8]; OR = 2.4 [1.8 - 3.1];  aOR = 3.3 [1.7 - 6.4]; aOR = 1.6 [1.1 - 2.2]; aOR = 1.6 [1.1 - 2.3];  Drug use: OR = 2.7 [1.9 - 3.9]; aOR = 2.0 [1.3 - 3.1];  Depressive symptoms: OR = 2.0 [1.5 - 2.9]; aOR = 1.1 [0.7 - 1.6];  PTS symptoms: OR = 2.2 [1.6 - 3.2]; OR = 2.4 [1.6 - 3.6]; PTS symptoms: aOR = 1.8 [1.0 - 3.4]  Suicidality: OR = 2.9 [1.8 - 4.7];  Other outcome: aOR = 1.7 [1.1 - 2.6]; aOR = 1.7 [1.0 - 2.9] | HIV: aOR = 1.3 [0.8 - 2.0]; aOR = 1.0 [0.6 - 1.5] |
| Becker et al., 2024 (Fair)  [10] | Sex working assessed as "risk factor"  Inclusion criteria: Adolescent girls and young women aged 14–24,  sexually active,  recruited from “hotspots” (bars, nightclubs, hotels, public spaces),  and provided consent.  Exclusion criteria: None reported. | Cross sectional study | LMICs: African Region: Kenya | 2015 - 2015 | 533 | Lifetime prevalence: Sexual violence only: Unspecified: 28.9 [25.0 - 32.7] | - | - |
| Berger et al., 2018 (Good)  [11] | Sex workers addressed only;  Inclusion criteria: Women aged ≥18 years  who reported exchanging sex for money or goods in the last 12 months,  willing to undergo HIV and syphilis testing,  and able to provide informed verbal consent in English or siSwati.  Exclusion criteria: None reported. | Cross sectional study | LMICs: African Region: Swaziland | July 2011 - September 2011 | 325 | Lifetime prevalence: Sexual violence only: Unspecified: 39.7 [34.2 - 45.1];  Police: 1.3 [0.0 - 2.5];  Partner: 5.2 [2.7 - 7.6];  Other: 8.4 [5.3 - 11.5];  Other: 6.1 [3.5 - 8.8];  Harassment: 61.1 [55.8 - 66.4];  Lifetime prevalence: Mixed types of violence: Unspecified: 59.0 [53.6 - 64.3] | Drug use: OR = 2.3 [1.4 - 3.8];  Suicidality: OR = 2.0 [1.3 - 3.2];  PTS symptoms: aOR = 1.6 [1.0 - 2.8] | - |
| Bhardwaj et al., 2023 (Good)  [12–14] | Sex workers addressed only;  Inclusion criteria: Cisgender women  aged ≥18 years,  reporting sex work as their primary source of income,  diagnosed with HIV for ≥6 months,  non-pregnant.  Exclusion criteria: None reported. | Cross sectional study | LMICs: African Region: South Africa | March 2020 | 1384 | Lifetime prevalence: Sexual violence only: Unspecified: 37.6 [35.0 - 40.1];  Lifetime prevalence: Mixed types of violence: Unspecified: 61.3 [58.7 - 63.8] | Depressive symptoms: PR = 1.6 [1.4 - 1.9]; aPR = 1.5 [1.2 - 1.7] | - |
| Bhattacharjee et al., 2015 (Fair)  [15] | Sex workers included as subgroup;  Inclusion criteria: None reported.  Exclusion criteria: None reported. | Cross sectional study | LMICs: African Region: Kenya | 2013 - 2014 | 3448 | 6-months prevalence: Mixed types of violence: Unspecified: 22.4 [21.0 - 23.8] | - | - |
| Bhattacharjee et al., 2020 (Fair)  [16] | Sex workers included as subgroup;  Inclusion criteria: Cisgender females aged 14–24 years,  having engaged in vaginal or anal sex at least once in their lifetime.  Exclusion criteria: None reported. | Cross sectional study | LMICs: African Region: Kenya | 2015 | 585 | Lifetime prevalence: Sexual violence only: Unspecified: 28.9 [25.2 - 32.6];  Past year prevalence: Sexual violence only: Unspecified: 14.2 [11.4 - 17.0] | - | - |
| Bitty-Anderson et al., 2024 (Fair)  [17] | Sex workers addressed only;  Inclusion criteria: Self-identified sex workers (defined as having engaged in transactional sex—sex in exchange for money or goods with clients rather than emotional partners—within the past 6 months),  aged ≥18 years,  in possession of a recruitment coupon.  Exclusion criteria: None reported. | Cross sectional study | LMICs: African Region: Togo | June 2021 - July 2021 | 447 | 6-months prevalence: Sexual violence only: Workplace: 11.2 [8.3 - 14.1];  6-months prevalence: Mixed types of violence: Workplace: 61.5 [57.0 - 66.0] | - | Other STI than HIV: aOR = 1.5 [1.0 - 2.2];  Abortion: aOR = 1.5 [1.0 - 2.3] |
| Blumenthal et al., 2021 (Poor)  [18] | Sex working assessed as "risk factor";  Inclusion criteria: Cisgender women aged ≥18 years,  HIV-uninfected (confirmed by fourth-generation antigen/antibody assay or antibody assay plus HIV nucleic acid test),  creatinine clearance >60 mL/min,  English or Spanish speaking,  residing in Los Angeles or San Diego counties,  and at substantial risk for HIV (defined as having an HIV-infected partner ≥4 weeks, engaging in sex work, prior PEP use in the past year, bacterial STI in the past 6 months, or a partner with unknown HIV status and increased HIV risk due to injection drug use, bisexual behavior, sex for goods, recent incarceration, origin from a high-prevalence region, or intimate partner violence).  Exclusion criteria: Non reported. | clinical trial design | HICs: United States | June 2016 - September 2018 | 21 | Past year prevalence: Mixed types of violence: Partner: 76.2 [58.0 - 94.4] | - | - |
| Bukenya et al., 2019 (Good)  [19] | Sex workers addressed only;  Inclusion criteria: Female sex workers aged 15–49 years  who had been pregnant within the two years preceding the interview.  Exclusion criteria: Female sex workers  unable to provide informed consent due to illness or intoxication with alcohol and/or drugs at the time of screening. | Cross sectional study | LMICs: African Region: Uganda | May 2018 - August 2017 | 819 | Past year prevalence: Sexual violence only: Unspecified: 21.0 [18.2 - 23.8] | - | - |
| Cange et al., 2019, M, (Good)  [20, 21] | Sex workers addressed only;  Inclusion criteria: Individuals aged ≥18 years,  born female,  who sold sex within the past 12 months with the majority of income derived from sex work,  resided in one of the study sites for at least 3 months,  able to provide informed consent in French or a local language (Mòoré or Dioula),  and (for the quantitative phase) in possession of a valid study coupon.  Exclusion criteria: None reported. | Cross sectional study | LMICs: African Region: Burkina Faso | January 2013 - July 2013 | 696 | Lifetime prevalence: Sexual violence only: Unspecified: 40.9 [37.3 - 44.6]; 45.6 [41.9 - 49.3];  Workplace: 13.6 [11.1 - 16.2]; 7.5 [5.5 - 9.4]; 14.2 [11.6 - 16.8];  Police: 5.2 [3.5 - 6.8];  Partner: 13.1 [10.6 - 15.6];  Lifetime prevalence: Harassment: 59.3 [55.7 - 63.0];  Lifetime prevalence: Forced entry into sex work: 15.9 [13.2 - 18.6] | - | - |
| Chersich et al., 2014 (Good)  [22, 23] | Sex workers addressed only;  Inclusion criteria: Women aged ≥16 years  who reported receiving money in exchange for sex as part of their livelihood in the past 6 months,  were sexually active in the past 3 months,  tested HIV-negative at enrollment by rapid HIV test,  and were not pregnant.  Exclusion criteria: Individuals planning to travel or relocate elsewhere,  or participating in another HIV intervention study. | Cohort study | LMICs: African Region: Kenya | May 2006 - August 2006 | 400 | Past year prevalence: Sexual violence only: Partner: 22.2 [18.2 - 26.3]; 17.8 [14.1 - 21.6]; 10.8 [7.8 - 13.9];  Past year prevalence: Mixed types of violence: Partner: 55.0 [50.0 - 60.1] | Alcohol use: OR = 4.2 [2.0 - 8.8]; aOR = 4.1 [1.9 - 8.9] | - |
| Davis & Miles, 2020, (Fair)  [24] | Sex workers addressed only;  Inclusion criteria: Individuals self-identified as “masseurs,”  who were readily visible and willing and/or permitted to be interviewed.  Exclusion criteria: None reported. | Mixed-method research  Excluded from qualitative meta-aggregation due to lack of qualitative data | LMICs: Western Pacific Region: Philippines | - | 51 | Lifetime prevalence: Sexual violence only: Unspecified: 37.3 [24.0 - 50.5] | - | - |
| Decker et al., 2013, M (Good)  [25] | Sex workers addressed only;  Inclusion criteria: Women,  including transwomen,  aged ≥18 years who traded sex for drugs, money, or a place to stay within the past three months.  Exclusion criteria: None reported. | Mixed-Method research | HICs: United States | March 2012 - August 2012 | 35 | 6-months prevalence: Sexual violence only: Workplace: 20.0 [6.7 - 33.3];  Police: 5.7 [-2.0 - 13.4  Partner: 8.6 [-0.7 - 17.8];  6-months prevalence: Mixed types of violence: Unspecified: 42.9 [26.5 - 59.3];  Workplace: 28.6 [13.6 - 43.5];  Partner: 17.1 [4.7 - 29.6] | - | - |
| Decker et al., 2016 (Fair)  [26] | Sex workers addressed only;  Inclusion criteria: Individuals aged ≥18 years,  self-determined as female,  who reported that selling sex in the past 12 months accounted for more than half of their income,  and able to provide informed consent in English or French.  Exclusion criteria: None reported. | Cross sectional study | LMICs: African Region: Cameroon | - | 1817 | Lifetime prevalence: Mixed types of violence: Unspecified: 60.4 [58.2 - 62.7] | - | HIV: aOR = 2.0 [1.2 - 3.3] |
| Decker et al., 2017 (Good)  [27] | Sex workers addressed only;  Inclusion criteria: Women aged ≥18 years  working as dancers in Baltimore City and County,  who had danced on at least three occasions in the past month and had been dancing for six months or less.  Exclusion criteria: None reported. | Cohort study | HICs: United States | May 2014 - October 2014 | 117 | 6-months prevalence: Sexual violence only: Workplace: 6.0 [1.7 - 10.3]; 3.4 [0.1 - 6.7]  Partner: 10.9 [4.8 - 17.0]; 3.0 [-0.3 - 6.3];  6-months prevalence: Mixed types of violence: Workplace: 16.2 [9.6 - 22.9];  Partner: 35.6 [26.3 - 45.0] | Drug use: aOR = 1.3 [0.4 - 3.9]; aOR = 3.0 [0.7 - 12.2] | - |
| Decker et al., 2017 (Good)  [28, 29] | Sex worker addressed only;  Inclusion criteria: Sex workers aged ≥18 years who traded sex for drugs, money, or other resources in the past 3 months and were using Baltimore City Health Department services.  Exclusion criteria: None reported. | Non-randomised experimental study,  Mixed-method research  Excluded from qualitative meta-aggregation due to mixed sample with participants with forced entry into sex work | HICs: United States | March 2015 - July 2015 | 60 | 6-months prevalence: Mixed types of violence: Workplace: 36.7 [24.5 - 48.9];  Partner: 56.5 [36.3 - 76.8] | - | - |
| Deuba et al., 2016 (Good)  [30] | Sex workers addressed only;  Inclusion criteria: Individuals aged ≥16 years  who reported receiving payment in cash or kind for sex with a male in the past 6 months,  residing in one of 22 Terai Highway districts of Nepal (Jhapa, Morang, Sunsari, Saptari, Siraha, Dhanusha, Mahottari, Sarlahi, Dhading, Makwanpur, Rautahat, Bara, Parsa, Chitwan, Nawalparasi, Rupandehi, Kapilvastu, Dang, Banke, Bardiya, Kailali, Kanchanpur).  Exclusion criteria: None reported. | Cross sectional study | LMICs: South-East Asia Region: Nepal | September 2012 - November 2012 | 610 | Past year prevalence: Sexual violence only: Unspecified: 20.5 [17.3 - 23.7];  Workplace: 25.1 [21.6 - 28.5] | - | - |
| Figueroa et al., 2015 (Good)  [31] | Sex working assessed as "risk factor";  Inclusion criteria: Individuals aged ≥16 years  who self-identified as MSM,  completed the interview,  provided blood and urine for HIV/STI testing,  and signed informed consent.  Exclusion criteria: None reported. | Cross sectional study | LMICs: Region of the Americas: Jamaica | 2011 | 95 | Lifetime prevalence: Sexual violence only: Unspecified: 18.9 [11.1 - 26.8] | - | - |
| Gerassi et al., 2016 (Poor)  [32] | Sex working assessed as "risk factor";  Inclusion criteria: None reported.  Exclusion criteria: Individuals who had never had sexual contact with males or females (n = 508);  individuals whose race/ethnicity was classified as other or unknown—outside of White, Black or African American, or multiracial—due to small numbers (n = 176). | Cross sectional study | HICs: United States | 2008 - 2014 | 128 | Lifetime prevalence: Sexual violence only: Unspecified: 35.2 [26.9 - 43.4] | - | - |
| Giorgio et al., 2016 (Good)  [33] | Sex working assessed as "risk factor";  Inclusion criteria: Women aged 16–39 years,  born outside South Africa, residing in Cape Town,  and able to speak one of the following study languages: English, Shona, Swahili, Lingala, Kirundi, Kinyarwanda, French, or Somali.  Exclusion criteria: None reported. | Cross sectional study | LMICs: African Region: South Africa | 2012 | 285 | Lifetime prevalence: Sexual violence only: Unspecified: 17.5 [13.1 - 22.0] | - | - |
| Grosso et al., 2023 (Good)  [34, 35] | Sex workers addressed only;  Inclusion criteria: Individuals aged ≥18 years,  assigned female at birth,  reporting sex work as their primary income source in the past 12 months,  capable of providing verbal informed consent,  spoke and understood English or French,  had not participated in a similar study in the past six months,  presented a valid unduplicated RDS coupon (except seeds), and resided in the city for the past three months.  Exclusion criteria: None reported. | Cross sectional study | LMICs: African Region: Cameroon | December 2015 - October 2016 | 2220 | 6-months prevalence: Sexual violence only: Unspecified: 10.5 [9.2 - 11.7] | - | - |
| Hail-Jares et al., 2015 (Good)  [36] | Sex workers addressed only;  Inclusion criteria: Biologically female individuals aged 18–65 years,  self-identified as current commercial sex workers primarily soliciting clients on the street (not regularly working at indoor venues such as salons, karaoke bars, or massage parlors),  and able to provide verbal or written consent in Mandarin.  Exclusion criteria: None reported. | Cross sectional study | LMICs: Western Pacific Region: China | 2011 - 2012 | 218 | 6-months prevalence: Sexual violence only: Workplace: 3.2 [0.9 - 5.6];  Partner: 0.9 [-0.3 - 2.2];  6-months prevalence: Mixed types of violence: Workplace: 62.8 [56.4 - 69.3];  Partner: 24.3 [18.6 - 30.0] | - | - |
| Herpai et al., 2022 (Fair)  [37] | Sex workers addressed only;  Inclusion criteria: Cisgender female sex workers  aged ≥14 years  who had engaged in sex work for at least 3 months.  Exclusion criteria: None reported. | Cross sectional study | LMICs: European Region: Ukraine | September 2017 - October 2018 | 560 | Lifetime prevalence: Sexual violence only: Police: 6.4 [4.4 - 8.5];  6-months prevalence: Mixed types of violence: Other: 7.0 [4.9 - 9.1] | - | - |
| Hladik et al., 2017 (Good)  [38] | Sex workers addressed only;  Inclusion criteria: Female sex workers aged ≥15 years,  residing in greater Kampala,  who self-reported selling sex to one or more men in the 30 days preceding survey enrollment.  Exclusion criteria: Candidate recruits who received their coupons from strangers,  violating the RDS assumption that recruiter and recruit know each other. | Cross sectional study | LMICs: African Region: Uganda | June 2008 - April 2009 | 942 | 6-months prevalence: Sexual violence only: Unspecified: 32.7 [29.7 - 35.7];  Workplace: 25.8 [23.0 - 28.6];  Lifetime prevalence: Mixed types of violence: Unspecified: 46.0 [42.8 - 49.1] | - | HIV: PR = 1.1 [0.8 - 1.6];  PR = 0.9 [0.6 - 1.3] |
| Hoang et al., 2024 (Good)  [39] | Sex workers addressed only;  Inclusion criteria: Cisgender women aged ≥18 years in Haiphong city,  self-identified as female sex workers,  who reported trading sex for money, drugs, shelter, or other material benefits within the past month.  Exclusion criteria: None reported. | Cross sectional study | LMICs: Western Pacific Region: Vietnam | October 2022 - November 2022 | 310 | Lifetime prevalence: Sexual violence only: Workplace: 51.9 [46.4 - 57.5];  Partner: 17.4 [13.2 - 21.6];  Workplace: 6.8 [4.0 - 9.6]; Other: 4.2 [2.0 - 6.4]; 1.6 [0.2 - 3.0];  Past year prevalence: Sexual violence only: Workplace: 42.6 [37.1 - 48.1];  Partner: 16.1 [12.0 - 20.2];  Lifetime prevalence: Mixed types of violence: Unspecified: 72.6 [67.6 - 77.5];  Workplace: 70.0 [64.9 - 75.1];  Partner: 30.6 [25.5 - 35.8];  Other: 18.1 [13.8 - 22.3];  Past year prevalence: Mixed types of violence: Unspecified: 65.8 [60.5 - 71.1];  Workplace: 61.3 [55.9 - 66.7];  Partner: 28.7 [23.7 - 33.7];  Other: 14.2 [10.3 - 18.1] | - | - |
| Hosseini-Hooshyar et al., 2022 Good)  [40–45] | Sex workers addressed only;  Inclusion criteria: Women aged ≥18 years,  holding Iranian citizenship, living or working in the study city,  who reported having sexual intercourse in exchange for livelihood (money, goods, services, or drugs) with more than one client in the 12 months prior to the interview.  Exclusion criteria: None reported. | Cross sectional study | LMICs: Eastern Mediterranean Region: Iran | January 2015 - August 2015 | 1335 | Lifetime prevalence: Sexual violence only: Unspecified: 40.1 [37.4 - 42.7];  Past year prevalence: Sexual violence only: Unspecified: 16.8 [14.8 - 18.8]; Workplace: 16.9 [14.9 - 19.0] | Drug use: PR = 2.2 [1.5 - 3.3]; aPR = 1.5 [1.0 - 2.2]; aOR = 2.1 [1.2 - 3.4] | Abortion: PR = 1.2 [1.1 - 1.4]; aPR = 1.2 [1.0 - 1.4] |
| Hosseini Divkolaye et al., 2021 (Good)  [46] | Sex workers addressed only;  Inclusion criteria: Individuals aged >18 years  who reported exchanging sex for money during the previous 6 months.  Exclusion criteria: None reported. | Cross sectional study | LMICs: Eastern Mediterranean Region: Iran | 2017 | 263 | Lifetime prevalence: Sexual violence only: Unspecified: 72.2 [66.8 - 77.7];  Workplace: 4.2 [1.8 - 6.6];  Partner: 30.4 [24.9 - 36.0];  Other: 37.3 [31.4 - 43.1] | Drug use: OR = 2.6 [1.4 - 4.6]; aOR = 2.7 [1.0 - 6.9] | - |
| Jewkes et al., 2021 (Good)  [47–51] | Sex workers addressed only;  Inclusion criteria: Individuals aged ≥18 years,  assigned female at birth and currently identifying as female,  working within the site’s district,  who voluntarily sold or transacted in sex for financial gain (not necessarily for cash) in the past six months.  Exclusion criteria: Individuals aged <18 years  or who self-reported being victims of human trafficking. | Cross sectional study | LMICs: African Region: South Africa | January 2019 - July 2019 | 3005 | Past year prevalence: Sexual violence only: Workplace: 48.2 [46.4 - 50.0];  Police: 14.0 [12.8 - 15.3];  Partner: 31.3 [29.6 - 32.9];  Other: 55.8 [54.0 - 57.5];  Past year prevalence: Mixed types of violence: Workplace: 57.2 [55.4 - 59.0];  Police: 22.1 [20.6 - 23.6];  Partner: 45.8 [44.0 - 47.6] | Depressive symptoms: RR = 1.0 [0.9 - 1.1]; RR = 1.2 [1.1 - 1.3];  PTS symptoms: RR = 1.4 [1.2 - 1.6]; RR = 1.4 [1.3 - 1.6] | - |
| Jiwatram-Negrón & El-Bassel, 2019 (Good)  [52] | Sex working assessed as "risk factor";  Inclusion criteria: Individuals aged ≥18 years,  HIV-negative (confirmed via biological assays),  in a main, regular partnership of ≥6 months who intended to remain together for at least one year,  with at least one partner reporting illicit drug use in the prior 90 days (and currently in or seeking drug treatment),  unprotected sex with the other partner in the prior 90 days, and meeting additional HIV risk criteria (past 90-day sex with another person, injection drug use, or self-reported STI).  Exclusion criteria: None reported. | Randomised controlled trial | HICs: United States | 2005 | 97 | Lifetime prevalence: Sexual violence only: Partner: 36.5 [26.8 - 46.1];  6-months prevalence: Sexual violence only: Partner: 30.2 [21.0 - 39.4];  Lifetime prevalence: Mixed types of violence: Partner: 53.1 [43.1 - 63.1];  6-months prevalence: Mixed types of violence: Partner: 46.9 [36.9 - 56.9] | - | - |
| Karamouzian et al., 2016 (Fair)  [53] | Sex workers addressed only;  Inclusion criteria: Individuals aged ≥18 years,  holding Iranian citizenship,  residing in the study city,  with a history of practicing sex work for at least 6 months  and having exchanged sex for money, drugs, or goods at least once in the previous year,  and who consented to participate.  Exclusion criteria: None reported. | Cross sectional study | LMICs: Eastern Mediterranean Region: Iran | April 2010 - July 2010 | 863 | 6-months prevalence: Sexual violence only: Unspecified: 33.3 [30.1 - 36.4] | - | Abortion: OR = 2.0 [1.5 - 2.7]; aOR = 1.8 [1.2 - 2.5] |
| Kibone et al., 2025) (Fair)  [54] | Sex workers addressed only;  Inclusion criteria: Cisgender women aged ≥18 years,  actively engaged in sex work  Exclusion criteria: Transgender women. | Cross sectional study | LMICs: African Region: Uganda | February 2023 - April 2023 | 314 | Past year prevalence: Sexual violence only: Unspecified: 27.4 [22.5 - 32.3];  Past year prevalence: Mixed types of violence: Unspecified: 70.7 [65.7 - 75.7] | - | Abortion: OR = 3.5 [2.1 - 6.0]; aOR = 2.2 [0.9 - 5.6] |
| L'Engle et al., 2014 (Good)  [55–57] | Sex workers addressed only;  Inclusion criteria: Female sex workers aged ≥18 years  who self-reported exchanging oral, anal, or vaginal sex for gifts or money in the past 6 months,  were moderate-risk drinkers (AUDIT score 7–19),  had laboratory-confirmed negative tests for gonorrhea, chlamydia, and trichomoniasis at enrollment,  were registered APHIA program participants,  and lived in Mombasa with plans to remain for the next 12 months.  Exclusion criteria: None reported. | Randomised controlled trial | LMICs: African Region: Kenya | March 2011 - October 2012 | 818 | 6-months prevalence: Sexual violence only: Workplace: 32.4 [29.1 - 35.6];  Partner: 44.5 [40.6 - 48.4] | - | - |
| Logie et al., 2017  (Good)  [58] | Sex working assessed as "risk factor";  Inclusion criteria: Individuals aged ≥18 years,  residing in Jamaica,  who identified as transgender women (assigned male at birth and self-identify as women),  and able to provide informed consent.  Exclusion criteria: None reported. | Cross sectional study | LMICs: Region of the Americas: Jamaica | March 2015 - October 2015 | 71 | Lifetime prevalence: Sexual violence only: Unspecified: 64.8 [53.7 - 75.9] | - | - |
| Logie et al., 2021 (Good)  [59] | Sex workers addressed only;  Inclusion criteria: Individuals aged ≥18 years,  residing in Kingston, Ocho Rios, Montego Bay, or contiguous areas,  who self-identified as cisgender men, cisgender women, or transgender women,  reported sex work in the past 12 months (exchanging sex for money or other goods),  and were able to provide informed consent.  Exclusion criteria: None reported. | Cross sectional study | LMICs: Region of the Americas: Jamaica | - | 340 | Lifetime prevalence: Sexual violence only: Unspecified: 49.1 [43.8 - 54.4];  6-months prevalence: Sexual violence only: Other: 25.3 [20.7 - 29.9];  6-months prevalence: Mixed types of violence: Workplace: 34.1 [29.1 - 39.2] | - | - |
| Menza et al., 2020 (Poor)  [60] | Sex working assessed as "risk factor";  Inclusion criteria: Cisgender women aged 18–60 years,  residing in a census tract of the Portland-Hillsboro, Oregon–Vancouver, Washington metropolitan area,  able to complete the survey in English or Spanish,  reported sex with at least one opposite-sex partner in the prior 12 months,  and had low socioeconomic status (income below the federal poverty level or less than a high school education);  had not previously participated in the current survey cycle.    Exclusion criteria: Individuals who identified as transgender  or had sex only with same-sex partners in the prior 12 months. | Cross sectional study | HICs: United States | 2016 - | 47 | Past year prevalence: Sexual violence only: Partner: 53.2 [38.9 - 67.5] | - | - |
| Mimiaga et al., 2021 (Good)  [61] | Sex workers addressed only;  Inclusion criteria: Individuals aged ≥18 years,  assigned male sex at birth and identifying as male,  who reported having insertive or receptive anal sex with three or more men in exchange for money in the month prior to enrollment.  Exclusion criteria: None reported. | Cohort study | HICs: United States | 2014 - 2016 | 81 | 6-months prevalence: Mixed types of violence: Workplace: 14.8 [7.1 - 22.6] | - | - |
| Mishra et al., 2013 (Good)  [62] | Sex workers addressed only;  Inclusion criteria: Individuals aged ≥15 years  who provided sex to a man in exchange for money or other financial benefits.  Exclusion criteria: None reported. | Cross sectional study | LMICs: Eastern Mediterranean Region: Pakistan | 2006 | 3585 | 6-months prevalence: Sexual violence only: Unspecified: 15.7 [14.5 - 16.9]; | - | - |
|  |  |  |  | 2011 | 3332 | 6-months prevalence: Sexual violence only: Unspecified: 22.4 [21.0 - 23.8] | - | - |
| Mokinu et al., 2024 (Fair)  [63] | Sex workers addressed only;  Inclusion criteria: Female sex workers aged ≥18 years,  residing in Mombasa County,  who reported sexual intercourse with a client in exchange for money or goods in the past month,  were willing to provide informed consent,  and were visibly sober at the time of recruitment.  Exclusion criteria: None reported. | Cross sectional study | LMICs: African Region: Kenya | December 2014 - May 2015 | 159 | Past year prevalence: Sexual violence only: Workplace: 23.9 [17.3 - 30.5] | - | - |
| Montgomery et al., 2015 (Good)  [64] | Sex working assessed as "risk factor";  Inclusion criteria: Women aged 18–44 years  residing in census tracts with high HIV prevalence (top 30th percentile) and ≥25% of residents living below the US federal poverty threshold,  who reported at least one episode of unprotected vaginal or anal sex with a man in the past 6 months,  were willing to undergo HIV rapid testing and receive results,  and reported at least one additional personal or partner HIV risk factor in the past 6 months (e.g., drug use, STI diagnosis, binge drinking, exchanging sex) or incarceration in the past 5 years.  Exclusion criteria: History of a positive HIV test,  current enrollment in an HIV prevention trial,  current or past participation in an HIV vaccine trial,  or anticipated absence from the community for more than two consecutive months during the follow-up period. |  | HICs: United States | May 2009 - July 2010 | 893 | 6-months prevalence: Sexual violence only: Unspecified: 15.0 [12.7 - 17.3];  6-months prevalence: Mixed types of violence: Unspecified: 29.7 [26.7 - 32.7] | - | - |
| Moradi et al., 2022 (Good)  [65] | Sex workers addressed only;  Inclusion criteria: Individuals aged ≥16 years  who reported penetrative vaginal or anal sex for money with more than one male client in the past year,  lived or worked in the selected city for at least 1–2 months after the interview,  possessed a valid RDS coupon (except seeds/headers),  and provided initial informed consent to participate.  Exclusion criteria: None reported. | Cross sectional study | LMICs: Eastern Mediterranean Region: Iran | December 2019 - August 2020 | 1390 | Lifetime prevalence: Sexual violence only: Unspecified: 40.1 [37.6 - 42.7] | - | Abortion: OR = 1.9 [1.5 - 2.4]; aOR = 1.5 [1.2 - 2.0] |
| Muldoon et al., 2017 (Good)  [66–71] | Sex workers addressed only;  Inclusion criteria: Individuals aged ≥14 years  who engaged in some form of commercial sex work in the previous 30 days.  Exclusion criteria: None reported. | Cross sectional study | LMICs: African Region: Uganda | May 2011 - January 2012 | 400 | 6-months prevalence: Sexual violence only: Workplace: 18.8 [14.9 - 22.6]; 7.8 [5.1 - 10.4]; 2.0 [0.6 - 3.4];  6-months prevalence: Mixed types of violence: Workplace: 78.5 [74.5 - 82.5];  Police: 9.2 [6.4 - 12.1];  Partner: 16.5 [12.9 - 20.1] | Drug use: OR = 1.1 [0.7 - 1.7] | HIV: OR = 0.8 [0.5 - 0.8]; OR = 0.8 [0.5 - 1.4]; OR = 1.1 [0.5 - 1.5];  Abortion: OR = 0.7 [0.4 - 1.2] |
| Mutagoma et al., 2019 (Good)  [72] | Sex workers addressed only;  Inclusion criteria: Self-reported female sex workers  aged ≥15 years,  identified in hotspot areas as indicated by a female sex worker key informant.  Exclusion criteria: Individuals who declined to provide consent to participate in the survey. | Cross sectional study | LMICs: African Region: Rwanda | March 2015 | 1978 | Lifetime prevalence: Sexual violence only: Workplace: 18.1 [16.4 - 19.8]; 18.3 [16.5 - 20.0] | - | HIV: OR = 1.4 [1.3 - 1.6]; aOR = 1.2 [1.0 - 1.4];  Other STI than HIV: OR = 1.6 [1.5 - 1.7];  aOR = 1.5 [1.0 - 2.1] |
| Nabayinda et al., 2023 (Good)  [73, 74] | Sex workers addressed only;  Inclusion criteria: Individuals aged ≥18 years  who reported vaginal or anal sexual intercourse in exchange for money, alcohol, or other goods in the preceding 30 days,  and at least one episode of unprotected sexual intercourse in the past 30 days with a paying, casual, or regular partner.  Exclusion criteria: None reported. | Randomised controlled trial | LMICs: African Region: Uganda | June 2019 - March 2020 | 542 | 6-months prevalence: Sexual violence only: Partner: 25.1 [21.4 - 28.7];  6-months prevalence: Mixed types of violence: Partner: 54.2 [50.0 - 58.4] | - | - |
| Ouma et al., 2021 (Fair)  [75] | Sex workers addressed only;  Inclusion criteria: Female sex workers  aged ≥18 years  operating in Gulu.  Exclusion criteria: Individuals aged <18 years. | Cross sectional study | LMICs: African Region: Uganda | March 2020 - June 2020 | 300 | Lifetime prevalence: Sexual violence only: Workplace: 21.0 [16.4 - 25.6]; 58.7 [53.1 - 64.2];  Lifetime prevalence: Mixed types of violence: Workplace: 61.0 [55.5 - 66.5] | Alcohol use: OR = 1.3 [0.8 - 2.3];  Drug use: OR = 2.2 [1.3 - 3.9] | HIV: OR = 1.7 [1.1 - 2.8]; aOR = 1.9 [1.1 - 3.3] |
| Owen et al., 2020 (Good)  [76] | Sex workers addressed only;  Inclusion criteria: Individuals aged ≥16 years  who had exchanged sex for money, favors, or goods in the past year  and presented a valid recruitment coupon.  Exclusion criteria: None reported. | Cross sectional study | LMICs: African Region: Swaziland | July 2011 - September 2011 | 325 | Lifetime prevalence: Sexual violence only: Unspecified: 37.8 [32.6 - 43.1] | - | - |
| Parcesepe et al., 2015 (Fair)  [77] | Sex workers addressed only;  Inclusion criteria: Individuals aged ≥18 years  who reported vaginal or anal sex in exchange for money, alcohol, or other goods in the past 90 days,  met criteria for harmful alcohol use in the past year (AUDIT score ≥8),  and were currently enrolled in the NAF program.  Exclusion criteria: None reported. | Randomised controlled trial | LMICs: Western Pacific Region: Mongolia | 2008 - 2009 | 222 | Lifetime prevalence: Sexual violence only: Workplace: 52.3 [45.7 - 58.8];  Partner: 22.4 [14.2 - 30.7];  6-months prevalence: Sexual violence only: Workplace: 35.1 [28.9 - 41.4];  Partner: 17.3 [9.9 - 24.8] | - | - |
| Pedersen et al., 2022 (Good)  [78] | Sex workers addressed only;  Inclusion criteria: Individuals aged ≥18 years,  identifying as cisgender or transgender women,  who exchanged sex for money, goods, or services in the past 90 days,  were willing to undergo HIV and STI testing,  spoke Malay, Tamil, or English,  and were able to provide informed consent.  Exclusion criteria: None reported. | Cross sectional study | LMICs: Western Pacific Region: Malaysia | February 2016 - December 2016 | 492 | Lifetime prevalence: Sexual violence only: Unspecified: 17.3 [13.9 - 20.6] | Drug use: OR = 1.7 [1.1 - 2.8]; aOR = 1.0 [0.4 - 2.2] | - |
| Pitpitan et al., 2014 (Good)  [79] | Sex working assessed as "risk factor";  Inclusion criteria: Venues with space for patrons to sit and drink,  reporting >50 unique patrons per week,  with >10% female patrons,  and willing to allow periodic research team visits over the course of a year.  Exclusion criteria: None reported. | Cross sectional study | LMICs: African Region: South Africa | October 2009 - May 2012 | 356 | 6-months prevalence: Sexual violence only: Unspecified: 55.6 [50.5 - 60.8] | - | - |
| Pokharel et al., 2024, M (Poor)  [80] | Sex working assessed as "risk factor";  Inclusion criteria: Transgender women  aged ≥18 years,  residing in Kathmandu Valley during data collection.  Exclusion criteria: Transgender women  aged <18 years,  not residing in Kathmandu Valley during data collection,  or unwilling to participate. | Cross sectional study | LMICs: South-East Asia Region: Nepal | July 2022 - December 2022 | 104 | Lifetime prevalence: Sexual violence only: Unspecified: 63.5 [54.2 - 72.7]; 83.7 [76.5 - 90.8] | - | - |
| Poxon et al., 2023 (Fair)  [81] | Sex workers addressed only;  Inclusion criteria: Female sex workers  aged >18 years,  currently exchanging sex for money or goods,  and HIV-negative.  Exclusion criteria: None reported. | Cross sectional study | LMICs: African Region: Kenya | June 2019 - July 2019 | 217 | Lifetime prevalence: Sexual violence only: Workplace: 68.7 [62.5 - 74.8];  Partner: 42.9 [36.3 - 49.4];  Other: 22.6 [17.0 - 28.1];  Lifetime prevalence: Mixed types of violence: Workplace: 92.1 [86.8 - 97.3];  Partner: 85.1 [78.2 - 92.1] | - | - |
| Reilly et al., 2014 (Fair)  [82] | Sex workers addressed only;  Inclusion criteria: Individuals aged ≥16 years  working in brothel-based commercial sex markets,  who provided sexual services in exchange for money in the past 3 months,  and were able to speak and understand Vietnamese.  Exclusion criteria: None reported. | Cross sectional study | LMICs: Western Pacific Region: China | November 2010 - December 2010 | 187 | Lifetime prevalence: Sexual violence only: Unspecified: 8.0 [4.1 - 11.9];  Other: 7.5 [3.7 - 11.3] | - | - |
| Rivera et al., 2021) (Good)  [83] | Sex workers addressed only;  Inclusion criteria: Cisgender women aged 18–60 years,  residents of the NYC metropolitan area,  able to speak English or Spanish,  who reported vaginal or anal sex with a male partner in the past 12 months  and receiving money or drugs in exchange for sex with a man in the past 12 months.  Exclusion criteria: Individuals who identified as male or transgender. | Cross sectional study | HICs: United States | August 2016 - December 2016 | 330 | Past year prevalence: Mixed types of violence: Workplace: 13.9 [10.2 - 17.7] | - | - |
| Roberts et al., 2018 (Good)  [84–87] | Sex workers addressed only;  Inclusion criteria: Women aged ≥16 years,  self-identifying as exchanging sex for payment in cash or in kind,  HIV-seronegative per their last rapid test,  not currently menstruating, pregnant, or <6 weeks postpartum,  residing within a one-day commuting distance to the study clinic,  able to provide informed consent,  and participating in monthly Mombasa Cohort procedures.  Exclusion criteria: None reported. | Cross sectional study | LMICs: African Region: Kenya | March 2014 - May 2015 | 283 | Lifetime prevalence: Sexual violence only: Other: 43.8 [38.0 - 49.6]; 26.1 [21.0 - 31.3];  Past year prevalence: Mixed types of violence: Unspecified: 39.6 [33.9 - 45.3];  Other: 39.9 [34.2 - 45.6]  Lifetime prevalence: Mixed types of violence: Workplace: 25.1 [20.0 - 30.1];  Partner: 59.0 [53.3 - 64.7];  Other: 86.9 [83.0 - 90.9]; | Alcohol use: aRR = 1.6 [1.1 - 2.2] | - |
| Roberts et al., 2020 (Fair)  [88] | Sex workers addressed only;  Inclusion criteria: Cisgender females aged 14–24 years  who answered “yes” to either “Presently, do you consider yourself a sex worker?” or “Were you ever a sex worker?”,  had ever engaged in vaginal or anal sex,  and were able to provide written informed consent.  Exclusion criteria: None reported. | Cross sectional study | LMICs: African Region: Kenya | April 2015 - November 2015 | 408 | Lifetime prevalence: Sexual violence only: Unspecified: 29.2 [24.8 - 33.6];  Past year prevalence: Sexual violence only: Unspecified: 14.2 [10.8 - 17.6] | - | - |
| Schwitters et al., 2015 (Fair)  [89] | Sex workers addressed only;  Inclusion criteria: Women aged ≥15 years  who sold sex to men in the 6 months prior to survey participation  and lived in Kampala.  Exclusion criteria: None reported. | Cross sectional study | LMICs: African Region: Uganda | April 2012 - December 2012 | 1467 | 6-months prevalence: Sexual violence only: Unspecified: 41.3 [38.8 - 43.8];  Workplace: 54.9 [52.4 - 57.5];  6-months prevalence: Mixed types of violence: Workplace: 81.9 [79.9 - 83.8] | Alcohol use: OR = 2.6 [1.6 - 4.1];  aOR = 1.4 [0.8 - 2.2] | - |
| Sherman et al., 2019 (Good)  [90–100] | Sex workers addressed only;  Inclusion criteria: Individuals aged ≥15 years  who sold or traded oral, vaginal, or anal sex for money or goods (e.g., food, drugs, favors),  picked up clients on the street or in public places ≥3 times in the past 3 months,  and were willing to undergo HIV and STI testing.  Exclusion criteria: Individuals identifying as male or a man. | Cohort study | HICs: United States | April 2016 - August 2017 | 312 | Lifetime prevalence: Sexual violence only: Workplace: 49.4 [43.8 - 54.9];  Police: 28.2 [23.2 - 33.2];  Partner: 24.4 [19.1 - 29.7];  6-months prevalence: Sexual violence only: Partner: 4.0 [1.6 - 6.4];  Lifetime prevalence: Forced entry into sex work: 7.4 [4.5 - 10.3];  Lifetime prevalence: Mixed types of violence: Unspecified: 81.2 [76.4 - 86.0];  Workplace: 57.2 [51.1 - 63.3];  Police: 38.4 [32.4 - 44.4];  Partner: 51.6 [45.4 - 57.8];  6-months prevalence: Mixed types of violence: Unspecified: 26.0 [20.6 - 31.4];  Workplace: 19.6 [14.7 - 24.5];  Police: 6.4 [3.4 - 9.4];  Partner: 8.0 [4.6 - 11.4] | - | - |
| Siconolfi et al., 2021 (Good)  [101] | Sex working assessed as "risk factor";  Inclusion criteria: Cisgender Black and/or African American men  who have sex with men (MSM),  aged 18–29 years,  who participated in Waves 5–7 (2013–2015) of the study.  Exclusion criteria: Men with missing data on lifetime experience of sexual violence (n = 22). | Cross sectional study | HICs: United States | 2013 - 2015 | 133 | Lifetime prevalence: Sexual violence only: Unspecified: 54.1 [45.7 - 62.6] | - | - |
| Sileo et al., 2018 (Fair)  [102] | Sex workers included as subgroup;  Inclusion criteria: Individuals aged ≥18 years,  not intoxicated at the time of the study,  who self-identified as fishermen, commercial sex workers (CSW), fisher sellers, or alcohol sellers.  Exclusion criteria: Women with no history of transactional sex (n = 53) and men (n = 132), as the sample sizes were insufficient to assess experiences of IPV. | Cross sectional study | LMICs: African Region: Uganda | - | 115 | Lifetime prevalence: Sexual violence only: Partner: 61.7 [52.9 - 70.6];  Past year prevalence: Sexual violence only: Partner: 53.9 [44.8 - 63.0] | - | HIV: OR = 3.3 [1.1 - 10.0] |
| Simmelink et al., 2022 (Good)  [103] | Sex workers addressed only;  Inclusion criteria: Women aged 16–34 years  who self-reported engaging in paid sex work in the past 6 months,  were not pregnant or planning pregnancy in the next 12 months,  resided within the study area,  and were able to read basic English text messages.  Exclusion criteria: Pregnant women,  those with medical conditions preventing enrollment,  not SMS literate,  already enrolled in the RCT or involved in study development,  did not own a mobile phone,  or had an unsupported phone provider. | Randomised controlled trial | LMICs: African Region: Kenya | September 2016 - July 2018 | 866 | Lifetime prevalence: Mixed types of violence: Partner: 75.1 [72.2 - 77.9];  Past year prevalence: Mixed types of violence: Partner: 60.6 [57.4 - 63.9] | - | Abortion: OR = 3.0 [1.6 - 5.7]; aOR = 2.6 [1.4 - 5.1] |
| Stenersen et al., 2022 (Poor)  [104] | Sex working assessed as "risk factor";  Inclusion criteria: Adults residing in the United States  who identify as transgender, trans, genderqueer, non-binary, or another non-cisgender identity.  Exclusion criteria: Participants with missing data on any of the variables of interest. | Cross sectional study | HICs: United States | December 2015 - | 5174 | Past year prevalence: Sexual violence only: Unspecified: 3.0 [2.1 - 3.9];  Past year prevalence: Sexual violence only: Police: 6.8 [5.5 - 8.1];  Past year prevalence: Mixed types of violence: Police: 33.5 [31.1 - 36.0] | - | - |
| Stoebenau et al., 2023 (Good)  [105] | Sex working assessed as "risk factor";  Inclusion criteria: Women aged 18–30 years,  not in formal education or employment,  and normally residing in selected informal settlements.  Exclusion criteria: None reported. | Randomised controlled trial | LMICs: African Region: South Africa | - | 396 | Past year prevalence: Sexual violence only: Other: 40.2 [35.3 - 45.0];  Past year prevalence: Mixed types of violence: Partner: 70.7 [66.2 - 75.2] | - | - |
| Štulhofer et al., 2017 (Fair)  [106–108] | Sex workers addressed only;  Inclusion criteria: None reported.  Exclusion criteria: None reported. | Cross sectional study | HICs: Croatia | February 2014 - May 2014 | 157 | Past year prevalence: Sexual violence only: Unspecified: 17.5 [11.5 - 23.5];  Lifetime prevalence: Sexual violence only: Other: 47.1 [39.3 - 54.9] | - | - |
| Tomko et al., 2023 (Good)  [109–112] | Sex workers addressed only;  Inclusion criteria: Cisgender women  aged ≥18 years  who sold or traded sex at least three times in the past three months.  Exclusion criteria: None reported. | Cohort study | HICs: United States | September 2017 - February 2019 | 385 | 6-months prevalence: Sexual violence only: Workplace: 25.7 [21.3 - 30.1];  Police: 5.8 [3.2 - 8.5]; 4.9 [2.5 - 7.3]; Police: 3.2 [1.3 - 5.2];  Unspecified: 4.5 [2.2 - 6.9];  6-months prevalence: Mixed types of violence: Other: 40.9 [35.4 - 46.4] | - | - |
| Tsai et al., 2016 (Good)  [113] | Sex workers addressed only;  Inclusion criteria: Women  aged ≥18 years  who reported engaging in sexual intercourse in exchange for money, alcohol, or other goods in the past 90 days,  had at least one episode of unprotected sex with a paying partner in the past 90 days,  and expressed interest in learning about and developing additional income sources.  Exclusion criteria: None reported. | Randomised controlled trial | LMICs: Western Pacific Region: Mongolia | November 2011 - July 2013 | 107 | 6-months prevalence: Sexual violence only: Workplace: 25.2 [17.0 - 33.5];  6-months prevalence: Mixed types of violence: Workplace: 43.9 [34.5 - 53.3] | - | - |
| Urada et al., 2014 (Fair)  [114] | Sex working assessed as "risk factor";  Inclusion criteria: None reported.  Exclusion criteria: None reported. | Cross sectional study | LMICs: Western Pacific Region: Philippines | April 2009 - January 2010 | 167 | Lifetime prevalence: Sexual violence only: Unspecified: 35.3 [28.1 - 42.6] | - | - |
| Wang et al., 2021 (Good)  [115] | Sex workers addressed only;  Inclusion criteria: Individuals aged ≥18 years  who self-reported engaging in sex work in the past 90 days,  identified as cisgender or transgender women,  were able to speak Bahasa Malaysia, Tamil, or English,  and could provide informed consent.  Exclusion criteria: None reported. | Cross sectional study | LMICs: Western Pacific Region: Malaysia | 2014 - 2015 | 469 | Lifetime prevalence: Sexual violence only: Unspecified: 17.5 [14.0 - 20.9] | - | HIV: OR = 3.3 [1.2 - 9.1];  aOR = 4.1 [0.7 - 24.2] |
| Wechsberg et al., 2017 (Fair)  [116] | Sex working assessed as "risk factor";  Inclusion criteria: Black African women  aged ≥15 years (15–17 years with evidence of tacit emancipation),  who used at least one substance weekly in the past 3 months,  had unprotected sex with a male partner in the past 6 months,  spoke English, Sesotho, Zulu/Xhosa, or Setswana,  provided informed consent,  and planned to remain in Pretoria for the next 12 months.  Exclusion criteria: None reported. | Randomised controlled trial | LMICs: African Region: South Africa | - | 262 | Lifetime prevalence: Mixed types of violence: Other: 41.6 [35.6 - 47.6] | - | - |
| Wells et al., 2018 (Good)  [117] | Sex workers addressed only;  Inclusion criteria: Individuals aged ≥18 years,  assigned female at birth,  currently living in Nelson Mandela Bay Municipality (NMBM),  who reported sex work as their principal source of income in the past year,  were HIV-positive and aware of their status,  presented a valid study coupon,  and could communicate in English or Xhosa.  Exclusion criteria: None reported. | Cross sectional study | LMICs: African Region: South Africa | October 2014 - April 2015 | 213 | Lifetime prevalence: Sexual violence only: Workplace: 16.9 [11.9 - 21.9] | - | - |
| White et al., 2016 (Fair)  [118–123] | Sex workers addressed only;  Inclusion criteria: Individuals aged ≥16 years  who exchanged sex for cash, goods, or services,  were laboratory-confirmed HIV-positive,  and anticipated remaining in Mombasa for at least 2 years.  Exclusion criteria: None reported. | Cohort study | LMICs: African Region: Kenya | October 2012 - September 2014 | 405 | Lifetime prevalence: Sexual violence only: Partner: 18.2 [14.0 - 22.4];  Other: 16.0 [12.3 - 19.6];  Past year prevalence: Sexual violence only: Partner: 4.9 [2.6 - 7.3]; Other: 8.5 [5.7 - 11.3];  Lifetime prevalence: Mixed types of violence: Other: 39.4 [34.6 - 44.1];  Past year prevalence: Mixed types of violence: Partner: 18.2 [13.7 - 22.7];  Other: 12.9 [9.6 - 16.1] | Alcohol use: OR = 7.8 [2.4 - 25.4];  aOR = 4.4 [1.2 - 16.6]  Depressive symptoms: OR = 1.8 [0.6 - 3.5]; | Other STI than HIV: RR = 1.0 [0.6 - 1.6]; aRR = 0.9 [0.6 - 1.4] |
| Williams et al., 2019 (Fair)  [124] | Sex working assessed as "risk factor";  Inclusion criteria: Individuals aged ≥18 years  who self-reported injection drug use in the previous 30 days with visual evidence of recent injection  and were able to provide informed consent.  Exclusion criteria: None reported. | Cross sectional study | HICs: United States | April 2011 - April 2013 | 88 | Past year prevalence: Sexual violence only: Unspecified: 13.6 [6.5 - 20.8] | - | - |
| Wirtz et al., 2015 (Good)  [125, 126] | Sex workers addressed only;  Inclusion criteria: Individuals aged ≥18 years,  born female,  who reported earning the majority of their income through sex work in the past 12 months,  possessed a valid study coupon,  had lived in one of the study sites for at least 3 months,  and were able to provide informed consent in French or the local language (Mòoré or Dioula in Burkina Faso; Ewe or Kabiye in Togo).  Exclusion criteria: None reported. | Cross sectional study | LMICs: African Region: Togo, Burkina Faso | - | 1379 | Lifetime prevalence: Sexual violence only: Unspecified: 33.0 [30.5 - 35.5];  Workplace: 11.4 [9.7 - 13.1]; Workplace: 1.7 [1.0 - 2.3];  Police: 4.0 [3.0 - 5.0];  Partner: 11.0 [9.4 - 12.7];  Other: 5.2 [4.0 - 6.4]; 11.0 [9.4 - 12.7] | - | - |
| Xie et al., 2023 (Fair)  [127] | Sex workers addressed only;  Inclusion criteria: Individuals aged ≥18 years,  assigned female at birth,  who self-reported engaging in transactional sex for money or property in the past month,  had not been tested for gonorrhea or chlamydia in the past 12 months or could not confirm the timing of prior testing,  engaged in high-risk sexual activities (condomless sex, multiple partners, transactional sex, alcohol- or drug-influenced sex) since their last test,  were willing to participate in the survey,  and provided informed consent.  Exclusion criteria: None reported. | Randomised controlled trial | LMICs: Western Pacific Region: China | - | 480 | Lifetime prevalence: Sexual violence only: Workplace: 2.9 [1.4 - 4.4];  Lifetime prevalence: Mixed types of violence: Workplace: 13.5 [10.5 - 16.6] | - | Other STI than HIV: OR = 4.0 [1.8 - 9.1]; aOR = 4.3 [1.7 - 10.6] |
| Yeo et al., 2022 (Good)  [128, 129] | Sex workers addressed only;  Inclusion criteria: Cisgender women  aged ≥18 years  who sold sex within the past 6 months in the respective district,  possessed a valid study-specific recruitment coupon,  and provided voluntary informed consent.  Exclusion criteria: None reported. | Cross sectional study | LMICs: African Region: South Africa | February 2016 - 2018 | 664 | Lifetime prevalence: Sexual violence only: Unspecified: 39.2 [34.9 - 43.4];  Workplace: 17.9 [14.6 - 21.2];  Police: 5.9 [3.9 - 8.0];  Partner: 24.4 [20.7 - 28.1];  Past year prevalence: Sexual violence only: Unspecified: 31.7 [27.6 - 35.7]; 4.9 [3.0 - 6.8];  Workplace: 14.6 [11.5 - 17.6];  Police: 3.9 [2.2 - 5.6];  Partner: 20.7 [17.1 - 24.2];  Past year prevalence: Sexual violence only: Unspecified: 10.8 [8.1 - 13.5];  Past year prevalence: Mixed types of violence: Partner: 52.6 [48.8 - 56.4]; Police: 51.8 [48.0 - 55.6] | Alcohol use: RR = 1.1 [0.9 - 1.2]; RR = 0.8 [0.7 - 0.9]; aRR = 0.8 [0.7 - 0.9];  Drug use: RR = 1.6 [1.3 - 1.8]; RR = 1.5 [1.3 - 1.8];  aRR = 1.4 [1.2 - 1.6]; aRR = 1.4 [1.2 - 1.6] | - |

Note. *N* = number of included sex workers in sample, CI = Confidence Interval, OR = Odds Ratio, PR = Prevalence Ratio, RR = Risk Ratio, M = was also quantitatively analysed (mixed-method study)

## Table A2: Included quantitative studies from countries with criminalisation of purchase of sex

| First Author, year  (Study quality)  [Publications used] | Targeted population | Study type | Region / country | Data collection time period | N | Sexual violence prevalences  % [95 %CI] | Mental health outcomes  OR/PR/RR  [95 % CI] | Sexual health outcomes  OR/PR/RR  [95 % CI] |
| --- | --- | --- | --- | --- | --- | --- | --- | --- |
| Bird et al., 2016 (Fair)  [130] | Sex workers addressed only;  Inclusion criteria: Respondents aged 18 years or older  who reported having exchanged sex for money or drugs in the past six months  and were able to provide written informed consent.  Exclusion criteria: None reported. | Cross sectional study | HICs: Canada | September 2009 - April 2010 | 340 | Lifetime prevalence: Sexual violence only: Unspecified: 57.9 [52.7 - 63.2] | - | - |
| Goldenberg et al., 2023  [131–151]  (Good) | Sex workers addressed only;  Inclusion criteria: Individuals aged 14 years or older,  residing in Metro Vancouver,  who reported having exchanged sex for money in the past 30 days,  self-identifying as a woman (including both cisgender and transgender women),  and able to provide informed consent.  Exclusion criteria: None reported. | Cohort study | HICs: Canada | January 2010 - February 2019 | 946 | 6-months prevalence: Sexual violence only: Workplace: 7.1 [5.4 - 8.7];  Partner: 8.1 [5.3 - 10.9];  Unspecified: 20.7 [18.1 - 23.3];  Other: 2.4 [1.4 - 3.4];  6-months prevalence: Mixed types of violence: Unspecified: 35.7 [32.6 - 38.8];  Workplace: 17.8 [15.3 - 20.2];  Police: 31.2 [28.2 - 34.1];  Partner: 14.3 [12.0 - 16.5] | Drug use: OR = 1.8 [1.4 - 2.4];  OR = 2.1 [1.6 - 2.9];  OR = 2.4 [1.9 - 3.0];  aOR = 1.6 [1.2 - 2.2];  aOR = 1.8 [1.4 - 2.4];  Suicidality: OR = 2.2 [1.8 - 2.9];  OR = 2.4 [1.9 - 3.0];  OR = 1.3 [1.0 - 1.6];  PTS symptoms: aOR = 1.8 [1.3 - 2.5];  aOR = 2.1 [1.6 - 2.8] | - |
| Harris et al., 2023 (Fair)  [152] | Sex working assessed as "risk factor";  Inclusion criteria: Individuals aged 18 years or older, living with HIV  and reporting drug use other than or in addition to cannabis (ACCESS),  or HIV-negative individuals at risk who reported injection drug use (VIDUS), who self-identified as women, and who completed a baseline and at least one follow-up interview between 2005 and December 2018.  Exclusion criteria: None reported. | Cohort study | HICs: Canada | 2005 - December 2018 | 327 | 6-months prevalence: Mixed types of violence: Unspecified: 45.9 [40.5 - 51.3] | - | - |
| Kuosmanen & de Cabo, 2021, M ()  [153] | Sex workers addressed only;  Inclusion criteria: Men who reported that at some point in their life they had sold sex  and who self-identified as men.  Exclusion criteria: Transgender respondents. | Cross sectional study | HICs: Sweden | 2011 - 2015 | 156 | Lifetime prevalence: Sexual violence only: Workplace: 27.0 [19.6 - 34.4]; 13.1 [7.5 - 18.8]  40.1 [31.9 - 48.4];  Unspecified: 16.8 [10.5 - 23.0]; | - | - |
| Landsberg et al., 2017 (Fair)  [154]  (same parent study as Prangnell et al., 2018 | Sex workers addressed only;  Inclusion criteria: Individuals engaging in sex work, defined as having exchanged sex for gifts, food, clothing, shelter, money, or drugs during the previous 6 months at each interview,  being 18 years or older,  reporting injection or illicit drug use,  having completed at least one study visit between December 1, 2008, and November 30, 2014, and residing in the Lower Mainland within the Vancouver Police Department jurisdiction.  Exclusion criteria: None reported. | Cohort study | HICs: Canada | December 2008 - November 2014 | 100 | 6-months prevalence: Mixed types of violence: Workplace: 8.0 [2.7 - 13.3] | - | - |
| Logie et al., 2022 (Fair)  [155] | Sex working assessed as "risk factor";  Inclusion criteria: Being 16 years or older  and HIV-positive.  Exclusion criteria: None reported. | Cohort study | HICs: Canada | 2013 - 2018 | 82 | 6-months prevalence: Mixed types of violence: Unspecified: 58.5 [47.9 - 69.2] | - | - |
| Mosnier et al., 2024 (Fair)  [156] | Sex workers addressed only;  Inclusion criteria: Individuals having provided sexual services for money or other compensation in the last 12 months,  being 18 years or older,  identifying as a cisgender or transgender woman,  born abroad,  working, living, or regularly passing through Marseille,  and having signed a free and informed consent to participate in the study.  Exclusion criteria: None reported. | Cohort study,  Mixed-method research  Excluded from qualitative meta-aggregation due to lack of qualitative data | HICs: France | April 2022 - November 2022 | 132 | Past year prevalence: Mixed types of violence: Unspecified: 15.2 [9.0 - 21.3] | - | - |
| Prangnell et al., 2018 (Good)  [157]  (same parent study as Landsberg et al., 2017) | Sex workers addressed only;  Inclusion criteria: Reported sex work (exchanging sex for gifts, food, clothing, shelter, money, or drugs),  18 years or older,  using illicit drugs,  female,  completed study visits between December 1, 2008 and November 30, 2014,  residing in the greater Vancouver region within the VPD jurisdiction in the previous six months at each follow-up,  and provided written informed consent.  Exclusion criteria: None reported. | Cohort study | HICs: Canada | December 2008 - November 2014 | 259 | 6-months prevalence: Sexual violence only: Workplace: 7.7 [4.5 - 11.0];  1.9 [0.3 - 3.6];  6-months prevalence: Mixed types of violence: Workplace: 12.4 [8.3 - 16.4] | Alcohol use: OR = 0.9 [0.5 - 1.7]; Drug use: OR = 2.3 [1.4 - 3.8]; aOR = 2.0 [1.1 - 3.6]  Mental health disorder diagnosis ever: OR = 1.4 [0.7 - 2.7] | HIV: OR = 0.7 [0.4 - 1.4] |
| Richardson et al., 2015 (Fair)  [158] | Sex working assessed as "risk factor";  Inclusion criteria: Adults engaged in sex work  who had recently injected or used illicit drugs (excluding cannabis),  living in Greater Vancouver, Canada,  and able to provide informed consent.  Exclusion criteria: None reported. | Cohort study | HICs: Canada | December 2005 - November 2012 | 234 | 6-months prevalence: Mixed types of violence: Unspecified: 28.6 [22.8 - 34.4] | - | - |
| Swaich et al., 2023 (Poor)  [159] | Sex working assessed as "risk factor";  Inclusion criteria: Individuals aged ≥18 or 14–26;  reported drug use in the past six months;  provided valid responses on gender identity  and experiences of violence in the past six months.  Exclusion criteria: Transgender or other non-binary gender | Cohort study | HICs: Canada | July 2020 - November 2020 | 65 | 6-months prevalence: Mixed types of violence: Unspecified: 30.8 [19.5 - 42.0] | - | - |

Note. *N* = number of included sex workers in sample, CI = Confidence Interval, OR = Odds Ratio, PR = Prevalence Ratio, RR = Risk Ratio, M = was also quantitatively analysed (mixed-method study)

## Table A3: Included quantitative studies from countries with partial criminalisation

| First Author, year  (Study quality)  [Publications used] | Targeted population | Study type | Region / country | Data collection time period | N | Sexual violence prevalences  % [95 %CI] | Mental health outcomes  OR/PR/RR  [95 % CI] | Sexual health outcomes  OR/PR/RR  [95 % CI] |
| --- | --- | --- | --- | --- | --- | --- | --- | --- |
| Alemayehu et al., 2015 (Fair)  [160] | Sex workers addressed only;  Inclusion criteria: Commercial sex workers registered in Mekelle City.  Exclusion criteria: Sex workers who were mentally ill during the data collection period. | Cross sectional study | LMICs: African Region: Ethiopia | April 2014 - - | 250 | Lifetime prevalence: Sexual violence only: Unspecified: 75.6 [70.3 - 80.9];  42.0 [35.9 - 48.1];  Other: 42.8 [36.7 - 48.9]; 32.8 [27.0 - 38.6] | Drug use: OR = 6.0 [2.5 - 14.7];  aOR = 5.4 [1.8 - 16.2] | - |
| Amogne et al., 2019 (Good)  [161] | Sex workers addressed only;  Inclusion criteria: Individuals aged ≥15 years who reported receiving money or other benefits for sex with four or more people in the last 30 days, were recruited through peer referral (coupon-based), and provided consent for interview and blood sampling.  Exclusion criteria: None reported. | Cross sectional study | LMICs: African Region: Ethiopia | 2014 - 2014 | 4900 | Lifetime prevalence: Sexual violence only: Unspecified: 15.2 [14.2 - 16.2] | Alcohol use: OR = 1.7 [1.3 - 2.2];  aOR = 1.5 [1.1 - 2.1];  Drug use: OR = 1.5 [1.2 - 1.8];  aOR = 1.1 [0.8 - 1.4] | HIV: OR = 0.9 [0.7 - 1.1] |
| Aristegui et al., 2022 (Fair)  [162] | Sex workers addressed only;  Inclusion criteria: Sex workers aged ≥18 years who were engaged in sex work at the time of the survey or had engaged in sex work prior to the onset of the COVID-19 health crisis.  Exclusion criteria: None reported. | Cross sectional study | LMICs: Region of the Americas: Argentina | October 2020 - April 2021 | 173 | Past year prevalence: Mixed types of violence: Police: 44.5 [37.1 - 51.9] | Drug use: OR = 2.3 [1.1 - 5.0];  aOR = 2.9 [1.0 - 8.4] | HIV: OR = 3.3 [1.3 - 8.6] |
| Arumugam et al., 2022  (Good)  [163, 164] | Sex workers addressed only;  Inclusion criteria: Self-identified female sex workers aged ≥15 years who engaged in consensual sex in exchange for cash or payment in kind in the previous month.  Exclusion criteria: None reported. | Cross sectional study | LMICs: South-East Asia Region: India | October 2014 - November 2015 | 27007 | Past year prevalence: Sexual violence only: Unspecified: 17.9 [17.5 - 18.4] | Drug use: RR = 1.3 [0.7 - 2.5] | HIV: OR = 0.4 [0.2 - 0.8];  aOR = 0.8 [0.3 - 1.8] |
| Avila et al., 2017 (Fair)  [165] | Sex workers addressed only;  Inclusion criteria: Self-identified male-to-female transgender sex workers aged >18 years who had received money or goods in exchange for sexual services, either regularly or occasionally.  Exclusion criteria: None reported. | Cross sectional study | LMICs: Region of the Americas: Argentina | October 2006 - December 2009 | 273 | Lifetime prevalence: Sexual violence only: Unspecified: 25.7 [20.0 - 31.4] | - | HIV: OR = 1.1 [0.6 - 2.0]; Other STI than HIV: OR = 0.5 [0.3 - 1.0];  aOR = NA [1.0 - 4.7] |
| Ballester-Arnal et al., 2018 (Poor)  [166] | Sex working assessed as "risk factor";  Inclusion criteria: Not specified.  Exclusion criteria: Female-to-male (FTM) transgender individuals. | Cross sectional study | HICs: Spain | - | 16 | Lifetime prevalence: Sexual violence only: Unspecified: 12.5 [-3.7 - 28.7];  31.2 [8.5 - 54.0] | - | - |
| Beksinska et al., 2018 (Good)  [167] | Sex workers addressed only;  Inclusion criteria: Individuals aged 18–49 years who had received money or gifts in exchange for sex at least once in the past month.  Exclusion criteria: None reported. | Cross sectional study | LMICs: South-East Asia Region: India | July 2011 - August 2011 | 1111 | Past year prevalence: Sexual violence only: Unspecified: 21.9 [19.4 - 24.3];  Workplace: 7.2 [5.7 - 8.8]; 0.2 [-0.1 - 0.4];  Police: 0.9 [0.4 - 1.5];  Partner: 14.7 [12.6 - 16.8];  Other: 0.1 [-0.1 - 0.3]; 2.9 [1.9 - 3.9];  Past year prevalence: Mixed types of violence: Unspecified: 34.9 [32.1 - 37.7];  Workplace: 9.2 [7.5 - 10.9]; 0.3 [-0.0 - 0.6];  Police: 0.9 [0.3 - 1.5];  Partner: 27.1 [24.5 - 29.7];  Other: 4.2 [3.0 - 5.4] | - | HIV: OR = 0.7 [0.4 - 1.3];  OR = 0.3 [0.1 - 0.7]; OR = 1.8 [0.9 - 3.5];  aOR = 0.8 [0.4 - 1.5];  aOR = 0.4 [0.1 - 1.1];  aOR = 1.2 [0.6 - 2.4];  Other STI than HIV: OR = 1.7 [0.4 - 8.0];  OR = 6.7 [1.1 - 39.6];  aOR = 2.2 [0.5 - 9.2];  aOR = 25.0 [5.9 - 96.7] |
| Biello et al., 2017 (Fair)  [168] | Sex workers addressed only;  Inclusion criteria: Individuals aged ≥18 years, biologically male at birth and identifying as male, who reported engaging in insertive or receptive anal sex with another man in exchange for money, goods, favors, or gifts in the three months prior to enrollment.  Exclusion criteria: Hijra (transgender women). | pilot intervention study | LMICs: South-East Asia Region: India | July 2013 - June 2014 | 100 | Lifetime prevalence: Sexual violence only: Unspecified: 13.0 [6.4 - 19.6]; 43.0 [33.3 - 52.7] | - | - |
| Boothe et al., 2020 (Fair)  [169] | Sex workers included as subgroup;  Inclusion criteria: Biologically female individuals aged ≥15 years who had received money in exchange for sex from someone other than a steady partner in the six months preceding the survey.  Exclusion criteria: None reported. | Cross sectional study | LMICs: African Region: Mozambique | September 2011 - March 2012 | 1242 | Past year prevalence: Sexual violence only: Unspecified: 11.1 [9.4 - 12.9] | - | Other STI than HIV: OR = 2.5 [1.8 - 3.6];  aOR = 2.0 [1.3 - 2.9] |
| Bossard et al., 2022 (Good)  [170] | Sex workers addressed only;  Inclusion criteria: Women aged ≥13 years who were living and/or working in the district for the previous six months and had exchanged sexual intercourse with someone other than their main partner for money or goods in the last 30 days.  Exclusion criteria: None reported. | Cross sectional study | LMICs: African Region: Malawi | July 2019 - September 2019 | 363 | Lifetime prevalence: Sexual violence only: Unspecified: 47.6 [43.8 - 51.5] | - | - |
| Bradley et al., 2013 (Fair)  [171] | Sex workers addressed only;  Inclusion criteria: Not specified.  Exclusion criteria: Not specified. | Cohort study | LMICs: South-East Asia Region: India | 2011 - | 270 | Past year prevalence: Sexual violence only: Unspecified: 17.4 [12.9 - 21.9] | - | - |
| Bugssa et al., 2015 (Fair)  [172] | Sex workers addressed only;  Inclusion criteria: Female commercial sex workers living in the specified kebelles and who had been working as sex workers for at least 3 months preceding the study.  Exclusion criteria: None reported. | Cross sectional study | LMICs: African Region: Ethiopia | January 2013 - June 2013 | 319 | Lifetime prevalence: Sexual violence only: Unspecified: 13.8 [10.0 - 17.6] | - | HIV: OR = 3.1 [1.4 - 6.7]; aOR = 2.2 [1.4 - 6.3];  Other STI than HIV: OR = 1.7 [0.5 - 5.5];  aOR = 1.6 [0.5 - 5.1] |
| Chabata et al., 2020 (Fair)  [173, 174] | Sex workers addressed only;  Inclusion criteria: Individuals aged 18–24 years who explicitly exchanged sex for money, goods, or services in the past month and were HIV negative.  Exclusion criteria: None reported. | Cohort study | LMICs: African Region: Zimbabwe | April 2017 - July 2017 | 1842 | Past year prevalence: Sexual violence only: Unspecified: 16.0 [14.3 - 17.7];  Past year prevalence: Mixed types of violence: Police: 4.5 [3.5 - 5.4] | - | - |
| Crowell et al., 2017 (Fair)  [175] | Sex working assessed as "risk factor";  Inclusion criteria: Males aged >16 years in Abuja or >18 years in Lagos, presenting with a valid RDS coupon, who reported receptive or insertive anal intercourse with a male partner at least once in the 12 months prior to enrolment.  Exclusion criteria: Participants who did not report the number of male partners with whom they exchanged sex for money, drugs, food, shelter, or transportation in the past 12 months. | Cohort study | LMICs: African Region: Nigeria | March 2013 - March 2016 | 735 | Lifetime prevalence: Sexual violence only: Unspecified: 32.0 [28.6 - 35.3] | - | - |
| Daka et al., 2022 (Fair)  [176, 177] | Sex workers addressed only;  Inclusion criteria: Individuals aged ≥18 years who had been engaged in commercial sex for the last 3 months, living in Hawassa City, and willing to participate in the study.  Exclusion criteria: Individuals with apparent mental or physical illnesses limiting participation in an interview, and those not available during the study period. | Cross sectional study | LMICs: African Region: Ethiopia | 1st July 2019 - 31st November 2019 | 381 | Lifetime prevalence: Sexual violence only: Unspecified: 11.5 [8.3 - 14.8] | - | Other STI than HIV: OR = 3.1 [1.3 - 7.1];  aOR = 2.9 [1.2 - 9.0] |
| Davis et al., 2017 (Good)  [178] | Sex working assessed as "risk factor";  Female individuals aged ≥18 years, self-reporting a positive HIV status, and who were clients at one of the recruitment sites.  Exclusion criteria: None reported. | Cross sectional study | LMICs: European Region: Kazakhstan | September 2013 - December 2013 | 56 | Lifetime prevalence: Mixed types of violence: Unspecified: 36.4 [22.1 - 50.6] | - | - |
| El-Bassel et al., 2020 (Good)  [179] | Sex workers addressed only;  Inclusion criteria: Individuals aged >18 years who provided sex in exchange for money, goods, or services in the past 90 days, were not cognitively impaired, reported illicit drug use within the past 12 months, reported at least one incident of unprotected sex in the past 90 days, could communicate in Russian, and did not intend to move away from the study site in the following 12 months.  Exclusion criteria: None reported. | Cross sectional study | LMICs: European Region: Kazakhstan | February 2015 - May 2017 | 400 | Lifetime prevalence: Sexual violence only: Unspecified: 78.8 [74.7 - 82.8];  6-months prevalence: Sexual violence only: Partner: 28.7 [24.3 - 33.2];  Other: 27.0 [22.6 - 31.4];  Lifetime prevalence: Mixed types of violence: Unspecified: 89.8 [86.8 - 92.7];  6-months prevalence: Mixed types of violence: Partner: 51.5 [46.6 - 56.4];  Other: 40.0 [35.2 - 44.8] | Drug use: OR = 1.2 [0.9 - 1.7];  aOR = 1.3 [0.9 - 1.8] | - |
| Faini et al., 2022 (Fair)  [180] | Sex workers addressed only;  Inclusion criteria: Women aged 18–45 years, self-identifying as street-based, home-based, or brothel-based sex workers, who exchanged sex for money in the past month, resided in Dar es Salaam, considered themselves at increased risk for HIV, provided consent, and were willing to undergo pregnancy testing and HIV pre- and post-test counseling.  Exclusion criteria: None reported. | Cohort study | LMICs: African Region: Tanzania | October 2018 - December 2018 | 773 | 6-months prevalence: Sexual violence only: Unspecified: 18.4 [15.6 - 21.1] | - | HIV: PR = 2.0 [1.2 - 3.6] |
| Grosso et al., 2018 (Good)  [181] | Sex workers addressed only;  Inclusion criteria: Individuals aged ≥18 years, assigned female at birth, who sold sex within the past six months as a principal source of income, provided verbal informed consent in Sesotho or English, had a valid recruitment coupon, and had lived in Lesotho for at least the past three months.  Exclusion criteria: None reported. | Cross sectional study | LMICs: African Region: Lesotho | February 2014 - September 2014 | 744 | Lifetime prevalence: Sexual violence only: Unspecified: 29.9 [26.6 - 33.2];  Police: 4.2 [2.7 - 5.6];  Partner: 5.4 [3.8 - 7.0];  Other: 0.1 [-0.1 - 0.4];  Lifetime prevalence: Forced entry into sex work: 0.3 [-0.1 - 0.6] | - | - |
| Hendrickson et al., 2018 (Good)  [182, 183] | Sex workers addressed only;  Inclusion criteria: Individuals aged ≥18 years who exchanged sex for money within the past month.  Exclusion criteria: None reported. | Cross sectional study | LMICs: African Region: Tanzania | October 2015 - April 2016 | 496 | 6-months prevalence: Mixed types of violence: Unspecified: 39.7 [35.4 - 44.0] | Alcohol use: OR = 2.0 [1.4 - 2.8];  aOR = 1.9 [1.2 - 3.0];  Drug use: OR = 1.8 [1.2 - 2.6];  aOR = 1.4 [1.0 - 1.9] | HIV: OR = 1.1 [0.8 - 1.6];  aOR = 1.4 [0.9 - 2.0] |
| Hentges et al., 2024 (Good)  [184] | Sex working assessed as "risk factor";  Inclusion criteria: Individuals aged ≥18 years, assigned male at birth, identifying as a transvestite, transgender woman, or any other transfeminine gender identity, living, working, or studying in the study cities, and presenting a referral coupon from a known recruiter (RDS sampling).  Exclusion criteria: Being under the influence of alcohol or psychoactive substances to the extent that the interview was impossible. | Cross sectional study | LMICs: Region of the Americas: Brazil | December 2019 - July 2021 | 535 | Lifetime prevalence: Sexual violence only: Unspecified: 60.6 [56.4 - 64.7] | - | - |
| Javalkar et al., 2019 (Good)  [185] | Sex workers addressed only; Inclusion criteria: Individuals aged >18 years reporting an intimate partner in the last 6 months, or who had left such a partner within the past 6 months. An intimate partner was defined as a husband, boyfriend, lover, or live-in partner who is not paid for sex.  Exclusion criteria: None reported. | Cross sectional study | LMICs: South-East Asia Region: India | June 2014 - | 620 | 6-months prevalence: Sexual violence only: Workplace: 8.7 [6.5 - 10.9];  Partner: 6.6 [4.7 - 8.6]; 5.6 [3.8 - 7.5];  Unspecified: 3.4 [2.0 - 4.8];  6-months prevalence: Mixed types of violence:  Workplace: 16.3 [13.4 - 19.2];  Partner: 24.4 [21.0 - 27.7] | - | - |
| Katz et al., 2015, M (Good)  [186] | Sex workers addressed only;  Inclusion criteria: Females aged 18–35 who reported receiving money or goods in exchange for sexual services as a source of income in the last six months; for the quantitative component, participants were also required to be non-pregnant.  Exclusion criteria: None reported. | Cross sectional study | LMICs: South-East Asia Region: Bangladesh | May 2011 - July 2011 | 677 | Past year prevalence: Sexual violence only: Unspecified: 12.1 [9.7 - 14.6] | - | - |
| Lima et al., 2017 (Poor)  [187] | Sex workers addressed only;  Inclusion criteria: None reported.  Exclusion criteria: None reported. | Cross sectional study | LMICs: Region of the Americas: Brazil | August 2008 - July 2009 | 2523 | Lifetime prevalence: Sexual violence only: Unspecified: 37.8 [35.9 - 39.7] | - | - |
| de Lima et al., 2022 (Good)  [188] | Sex workers addressed only;  Inclusion criteria: Individuals aged ≥18 years who reported at least one sexual transaction in exchange for money in the past four months, identified as sex workers in one of the study cities, and presented a valid RDS coupon to participate.  Exclusion criteria: None reported. | Cross sectional study | LMICs: Region of the Americas: Brazil | 2016 - 2016 | 4188 | Lifetime prevalence: Sexual violence only: Unspecified: 28.6 [27.3 - 30.0] | - | - |
| Longo et al., 2023 (Fair)  [189] | Sex workers addressed only;  Inclusion criteria: Sexually active individuals with more than two sexual partners (other than their regular partner) in the last 3 months, who reported receiving money or gifts in exchange for sexual relationships, volunteered for the study, and provided oral consent.  Exclusion criteria: Individuals who had sexual relationships to obtain a job, were unwilling to participate in the study, or had good classroom results. | Cross sectional study | LMICs: African Region: Central African Republic | October 2021 - October 2021 | 480 | Past year prevalence: Sexual violence only: Unspecified: 37.5 [33.2 - 41.8] | - | - |
| Luiz et al., 2025 (Fair)  [190–192] | Sex workers addressed only;  Inclusion criteria: Female sex workers aged ≥15 years who, in the 6 months prior to the survey, received money for sexual services from someone other than their main partner, resided, worked, or socialized in the survey areas, were able to provide written informed consent, and possessed a valid referral coupon (per sampling methodology).  Exclusion criteria: None reported. | Cross sectional study | LMICs: African Region: Mozambique | September 2019 - 2020 | 2567 | 6-months prevalence: Sexual violence only: Unspecified: 37.9 [36.0 - 39.8] | Alcohol use: aOR = 1.4 [1.2 - 1.5];  Drug use: aOR = 2.6 [2.3 - 3.0] | HIV: aOR = 1.1 [1.0 - 1.2];  Other STI than HIV: aOR = 2.1 [1.9 - 2.3] |
| Lyons et al., 2017;  (Good)  [193, 194] | Sex workers addressed only;  Inclusion criteria: Women aged ≥18 years, assigned female at birth, engaging in sex work as their primary source of income within the past year, living primarily in Abidjan for at least the past 3 months, able to provide informed consent, able to speak French or English, and possessing a valid RDS coupon.  Exclusion criteria: None reported. | Cross sectional study | LMICs: African Region: Cote d'Ivoire | - | 466 | Lifetime prevalence: Sexual violence only: Unspecified: 43.2 [38.7 - 47.7] | - | HIV: OR = 1.0 [0.5 - 1.8] |
| MacLin et al., 2023 (Fair)  [195–197] | Sex workers addressed only;  Inclusion criteria: None reported.  Exclusion criteria: None reported. | Cross sectional study | LMICs: Region of the Americas: Dominican Republic | December 2018 - November 2019 | 311 | 6-months prevalence: Sexual violence only: Workplace: 4.5 [2.2 - 6.8];  6-months prevalence: Mixed types of violence:  Workplace: 10.9 [7.5 - 14.4];  Police: 8.0 [5.0 - 11.1]; 25.4 [20.6 - 30.2]  Partner: 6.4 [3.7 - 9.2] | - | - |
| Marquez et al., 2024 (Fair)  [198] | Sex workers addressed only;  Inclusion criteria: Women aged ≥16 years, assigned female at birth, currently practicing sex work, living in the study region for at least three months, and able to provide informed consent.  Exclusion criteria: Individuals with health conditions impeding understanding of the study or consent process, under the influence of substances, or who had previously participated in the study. | Cross sectional study | LMICs: African Region: Cote d'Ivoire | November 2019 - May 2020 | 1177 | Lifetime prevalence: Sexual violence only: Unspecified: 31.9 [29.3 - 34.6];  Past year prevalence: Sexual violence only: Unspecified: 10.2 [8.5 - 11.9] | Alcohol use: OR = 1.8 [1.1 - 2.9];  aOR = 1.6 [1.0 - 2.7];  Drug use: OR = 2.3 [1.5 - 3.7];  aOR = 2.4 [1.5 - 3.9];  Depressive symptoms: OR = 1.1 [0.8 - 1.4];  aOR = 1.1 [0.8 - 1.4];  Suicidality: OR = 1.9 [1.5 - 2.5];  PTS symptoms: aOR = 1.9 [1.5 - 2.5] | - |
| Milner et al., 2019 (Poor)  [199] | Sex working assessed as "risk factor";  Inclusion criteria: Transgender women who were assigned male at birth and identified and presented as women.  Exclusion criteria: None reported. |  | LMICs: Region of the Americas: Dominican Republic | 2015 - | 140 | Lifetime prevalence: Sexual violence only: Unspecified: 23.6 [16.5 - 30.6] | - | - |
| Mizinduko et al., 2020 (Good)  [200] | Sex workers addressed only;  Inclusion criteria: Individuals aged ≥18 years who had exchanged sex for money or goods at least once in the past three months and had been living in Dar es Salaam for the past six months before recruitment.  Exclusion criteria: None reported. | Cross sectional study | LMICs: African Region: Tanzania | September 2017 - December 2017 | 958 | Past year prevalence: Sexual violence only: Unspecified: 32.8 [29.8 - 35.8] | - | HIV: PR = 1.4 [1.0 - 2.1];  aPR = 1.9 [1.3 - 2.8] |
| Ngale et al., 2019 (Fair)  [201] | Sex workers addressed only;  Inclusion criteria: Individuals aged ≥15 years who received money from someone other than a main partner in exchange for sex in the six months preceding the survey.  Exclusion criteria: Women unable or unwilling to provide written informed consent or who did not present a valid survey coupon. | Cross sectional study | LMICs: African Region: Mozambique | September 2011 - March 2012 | 1240 | 6-months prevalence: Sexual violence only: Unspecified: 11.1 [9.4 - 12.9] | - | Other STI than HIV: OR = 2.1 [1.6 - 2.8];  aOR = 2.1 [1.6 - 2.9] |
| Ochonye et al., 2023 (Poor)  [202] | Sex workers addressed only;  Inclusion criteria: Female sex workers aged ≥15 years who were willing to participate in the study.  Exclusion criteria: Individuals aged <15 years, non-female, or unwilling to participate in the study. | Cross sectional study | LMICs: African Region: Nigeria | 2018 - 2020 | 223 | Lifetime prevalence: Sexual violence only: Unspecified: 8.1 [4.5 - 11.6]; 9.0 [5.2 - 12.7];  Lifetime prevalence: Mixed types of violence: Unspecified: 48.0 [41.4 - 54.5];  Workplace: 19.3 [14.1 - 24.5]; 10.3 [6.3 - 14.3];  Police: 8.1 [4.5 - 11.6];  Partner: 6.7 [3.4 - 10.0];  Unspecified: 1.8 [0.1 - 3.5] | - | - |
| Onoja et al., 2020 (Poor)  [203] | Sex workers addressed only;  Inclusion criteria: None reported.  Exclusion criteria: None reported. | Non-randomised experimental study | LMICs: African Region: Nigeria | 2006 - 2011 | 261 | Lifetime prevalence: Sexual violence only: Unspecified: 53.6 [47.6 - 59.7]; 26.1 [20.7 - 31.4] | - | - |
| Pando et al., 2013 (Good)  [204] | Sex workers addressed only;  Inclusion criteria: Individuals aged >18 years who had exchanged sex for money during the previous six months.  Exclusion criteria: None reported. | Cross sectional study | LMICs: Region of the Americas: Argentina | October 2006 - November 2009 | 1255 | Lifetime prevalence: Sexual violence only: Unspecified: 24.1 [21.4 - 26.9] | - | HIV: OR = 2.1 [0.8 - 5.8];  aOR = 1.4 [0.8 - 2.4];  Other STI than HIV: OR = 1.3 [0.9 - 1.9];  aOR = 1.1 [0.9 - 1.3] |
| Patel et al., 2015 (Fair)  [205] | Sex workers addressed only;  Inclusion criteria: Women aged ≥18 years who had sex in exchange for cash or kind in the past month.  Exclusion criteria: None reported. | Cross sectional study | LMICs: South-East Asia Region: India | November 2010 - January 2011 | 1986 | Lifetime prevalence: Forced entry into sex work: 12.2 [10.8 - 13.7] | - | - |
| Patel et al., 2016 (Good)  [206] | Sex workers addressed only;  Inclusion criteria: Individuals aged ≥18 years who had sex in exchange for cash or kind in the past month.  Exclusion criteria: None reported. | Cross sectional study | LMICs: South-East Asia Region: India | April 2014 - May 2014 | 2400 | Past year prevalence: Mixed types of violence: Unspecified: 24.0 [22.3 - 25.7];  Workplace: 8.2 [7.1 - 9.3]; 4.6 [3.8 - 5.5];  Police: 2.6 [2.0 - 3.3];  Partner: 6.0 [5.1 - 7.0];  Other: 2.5 [1.9 - 3.2] | Depressive symptoms: aOR = 3.0 [2.4 - 3.6]; aOR = 2.4 [1.8 - 3.2]; aOR = 5.0 [3.4 - 7.6]; aOR = 2.2 [1.5 - 3.1]; aOR = 7.4 [4.5 - 13.0]; aOR = 2.0 [1.2 - 3.4] | - |
| Platt et al., 2022  (Good)  [207, 208] | Sex workers addressed only;  Inclusion criteria: Sex workers aged ≥18 years who had exchanged in-person sexual services in the last 3 months in East London or elsewhere in London if advertising online.  Exclusion criteria: None reported. | Cohort study | HICs: United Kingdom | May 2018 - October 2019 | 274 | 6-months prevalence: Sexual violence only: Unspecified: 19.0 [14.3 - 23.6];  Police: 4.2 [1.8 - 6.6];  Lifetime prevalence: Mixed types of violence: Police: 32.1 [26.6 - 37.6];  6-months prevalence: Mixed types of violence:  Workplace: 36.2 [27.0 - 45.4];  Partner: 18.1 [10.7 - 25.5] | Drug use: OR = 4.9 [2.2 - 10.8];  aOR = 3.8 [1.1 - 10.1]; Depressive  Depressive symptoms: OR = 2.3 [1.2 - 4.5];  aOR = 2.5 [1.1 - 5.9] | - |
| Rameto et al., 2023 (Good)  [209–211] | Sex workers addressed only;  Inclusion criteria: Individuals aged ≥15 years who sold sex to at least four paying partners in the four weeks prior to the survey, resided or worked at the selected study sites for at least four weeks, provided informed consent for the bio-behavioral survey and biological testing, and possessed a valid coupon.  Exclusion criteria: None reported. | Bio-behavioral Survey / cross-sectional study | LMICs: African Region: Ethiopia | December 2019 - May 2020 | 6085 | Past year prevalence: Sexual violence only: Unspecified: 12.7 [11.8 - 13.5] | Alcohol use: OR = 2.3 [2.0 - 2.7];  Depressive symptoms: OR = 2.2 [1.9 - 2.6] | - |
| Sherwood et al., 2015 (Fair)  [212] | Sex workers addressed only;  Inclusion criteria: Individuals aged >16 years, born female, who sold sex for money, goods, or favors in the past 12 months, residing in The Gambia, and able to provide verbal consent in English, Wolof, or Mandinka.  Exclusion criteria: None reported. | Cross sectional study | LMICs: African Region: Gambia, The | July 2011 - August 2011 | 251 | Lifetime prevalence: Sexual violence only: Workplace: 27.9 [22.3 - 33.4] | Depressive symptoms: OR = 2.0 [1.1 - 3.7];  aOR = 2.1 [1.1 - 4.2] | (Unwanted) pregnancy: OR = 1.4 [0.7 - 2.7]  aOR = 2.7 [1.1 - 6.5] |
| Silverman et al., 2014 (Good)  [213] | Sex workers addressed only;  Inclusion criteria: Individuals aged ≥18 years, HIV-infected, who reported involvement in sex trade in the past year and penile-vaginal or anal sex in the past 30 days.  Exclusion criteria: None reported. | Cross sectional study | LMICs: South-East Asia Region: India | November 2008 - February 2009 | 211 | Lifetime prevalence: Forced entry into sex work: 41.7 [35.1 - 48.4] | - | Other STI than HIV: OR = 1.0 [0.6 - 1.7];  aOR = 1.0 [0.6 - 1.8] |
| Srivastava et al., 2022 (Fair)  [214] | Sex working assessed as "risk  Inclusion criteria: None reported.  Exclusion criteria: Participants with incomplete survey information, duplicate, or unmatched participant IDs | Cross sectional study | LMICs: South-East Asia Region: India | July 2017 - September 2017 | 1897 | 6-months prevalence: Sexual violence only: Unspecified: 5.4 [4.4 - 6.4] | - | - |
| Stephano, 2022 (Fair)  [215] | Sex workers addressed only;  Inclusion criteria: Female commercial sex workers who consented to participate in the study.  Exclusion criteria: FCSWs who were seriously ill or had pronounced mental illnesses. | Cross sectional study | LMICs: African Region: Tanzania | March 2019 - April 2019 | 326 | Lifetime prevalence: Sexual violence only: Unspecified: 51.8 [46.4 - 57.3] | - | - |
| Surti et al., 2017 (Fair)  [216] | Sex workers addressed only; Inclusion criteria:  Inclusion criteria: Female sex workers registered at Sahyog community based organisation who were rapid plasma regain reactive between April and August 2014.  Exclusion criteria: None reported. | Cohort study | LMICs: South-East Asia Region: India | September 2014 - September 2014 | 16 | Lifetime prevalence: Sexual violence only: Unspecified: 31.2 [8.5 - 54.0]; 25.0 [3.8 - 46.2];  Workplace: 6.2 [-5.6 - 18.1] | - | - |
| Szwarcwald et al., 2018 (Fair)  [217] | Sex workers addressed only;  Inclusion criteria: Individuals aged ≥18 years who reported working as a sex worker in one of the study cities, had at least one sexual intercourse in exchange for money in the past four months, and presented a valid coupon to participate.  Exclusion criteria: None reported. | Cross sectional study | LMICs: Region of the Americas: Brazil | July 2016 - November 2016 | 4328 | - | - | HIV: OR = 1.5 [1.0 - 2.3] |
| Tounkara et al., 2014 (Good)  [218] | Sex workers addressed only;  Inclusion criteria: Women aged ≥15 years.  Exclusion criteria: None reported. | Cross sectional study | LMICs: African Region: Benin | 2012 - 2012 | 981 | 6-months prevalence: Sexual violence only: Unspecified: 13.5 [11.3 - 15.6] | - | HIV: aPR = 1.4 [1.0 - 2.0] |
| Twizelimana & Muula, 2021 (Fair)  [219] | Sex workers addressed only;  Inclusion criteria: Self-reported female sex workers aged 18–49 years who consented to participate in the study.  Exclusion criteria: FSWs who were sick, participating in another study, or coming from outside the study locations. | Cross sectional study | LMICs: African Region: Malawi | February 2019 - March 2019 | 290 | Lifetime prevalence: Mixed types of violence: Workplace: 21.0 [16.3 - 25.7] | - | - |
| Urada et al., 2013 (Fair)  [220] | Sex working assessed as "risk factor";  Inclusion criteria: Individuals aged ≥18 years, HIV-positive, meeting ‘at risk’ drinking levels in the prior six months, fluent in Russian, reporting unprotected anal or vaginal sex in the past six months, and providing a stable local address and telephone number.  Exclusion criteria: Individuals with unconfirmed HIV infection, missing contact information, attempting pregnancy, having a pending legal issue, not meeting alcohol or sexual activity criteria, refusing to participate, or too ill to participate. | Non-randomised experimental study | HICs: Russia | October 2007 - April 2010 | 42 | Lifetime prevalence: Mixed types of violence: Unspecified: 92.9 [85.1 - 100.6] | - | - |
| Vélez-Grau et al., 2021 (Good)  [221] | Sex workers addressed only;  Inclusion criteria: Female individuals aged >18 years who reported providing sex in exchange for money, goods, drugs, or services in the past three months, reported any illicit drug use in the past year, and had at least one incident of unprotected sex with a paying or intimate partner in the past three months.  Exclusion criteria: Individuals with cognitive impairments affecting consent, unable to communicate in Russian, planning to move from the study area within the following year, or unable to fully participate in intervention activities. | Randomised controlled trial | LMICs: European Region: Kazakhstan | May 2015 - 2017 | 400 | Lifetime prevalence: Mixed types of violence: Partner: 86.2 [82.9 - 89.6] | Suicidality: OR = 0.9 [0.4 - 1.8] | - |
| Wirtz et al., 2015  (Good)  [222–225] | Sex workers addressed only;  Inclusion criteria: Female individuals aged ≥18 years, living in one of the three study sites, who had sold sex for money, drugs, or other items of value within the last three months.  Exclusion criteria: None reported. | Cross sectional study | HICs: Russia | July 2011 - September 2011 | 754 | 6-months prevalence: Sexual violence only: Workplace: 11.4 [9.1 - 13.7]; 6.2 [4.5 - 8.0]  Police: 5.0 [3.5 - 6.6];  Lifetime prevalence: Mixed types of violence: Unspecified: 44.8 [41.3 - 48.4];  Workplace: 31.7 [28.4 - 35.0]; 11.4 [9.1 - 13.7]  Police: 16.0 [13.4 - 18.7];  Partner: 15.6 [13.1 - 18.2] | Drug use: aOR = 3.3 [1.5 - 7.1]; aOR = 3.2 [1.2 - 8.7] | HIV: aOR = 3.8 [1.7 - 8.2];  aOR = 3.4 [0.9 - 12.9] |
| Witte et al., 2023 (Good)  [226] | Sex workers addressed only;  Inclusion criteria: Individuals aged ≥18 years who had provided sex in exchange for money, goods, drugs, or services within the past 90 days, reported illicit drug use in the past 12 months, and had at least one incident of unprotected sex with a paying or non-paying partner in the past 90 days.  Exclusion criteria: Individuals with cognitive impairments affecting consent or participation, unable to communicate in Russian, or intending to move from the study site within the next year. | Randomised controlled trial | LMICs: European Region: Kazakhstan | May 2015 - October 2018 | 354 | 6-months prevalence: Sexual violence only: Workplace: 21.5 [17.2 - 25.7  Partner: 17.2 [13.3 - 21.2]; 16.1 [12.3 - 19.9] | - | - |

Note. *N* = number of included sex workers in sample, CI = Confidence Interval, OR = Odds Ratio, PR = Prevalence Ratio, RR = Risk Ratio, M = was also quantitatively analysed (mixed-method study)

## Table A4: Included quantitative studies from countries with regulatory models

| First Author, year  (Study quality)  [Publications used] | Targeted population | Study type | Region / country | Data collection time period | N | Sexual violence prevalences  % [95 %CI] | Mental health outcomes  OR/PR/RR  [95 % CI] | Sexual health outcomes  OR/PR/RR  [95 % CI] |
| --- | --- | --- | --- | --- | --- | --- | --- | --- |
| George et al., 2016 (Good)  [227] | Sex workers addressed only;  Inclusion criteria: Individuals aged ≥15 years,  born male and self-identifying as male,  who had traded sex for money, goods, or services at least once in the previous week,  resided in Lima,  and were able and willing to provide informed consent.  Exclusion criteria: None reported. | Cross sectional study | LMICs: Region of the Americas: Peru | January 2014 - August 2014 | 210 | 6-months prevalence: Sexual violence only: Unspecified: 16.2 [11.2 - 21.2];  6-months prevalence: Mixed types of violence: Workplace: 16.2 [11.2 - 21.2];  Partner: 31.9 [25.6 - 38.2]; Other: 42.4 [35.7 - 49.1] | - | HIV: PR = 1.6 [1.2 - 2.2]; aPR = 1.6 [1.0 - 2.6] |
| Kloek & Dijkstra, 2018, M (Fair)  [228] | Sex workers addressed only;  Inclusion criteria: Licensed or non-licensed sex workers in the Netherlands  with real-life client contact, regardless of the type of sex work performed,  able to complete the questionnaire in Dutch, English, or Spanish.  Exclusion criteria: Sex workers working exclusively via webcam or telephone,  and those not sufficiently fluent in Dutch, English, or Spanish. | Cross sectional study | HICs: Netherlands | January 2017 - September 2017 | 299 | Past year prevalence: Sexual violence only: Unspecified: 77.9 [73.2 - 82.6]; 38.1 [32.6 - 43.6];  Workplace: 30.1 [24.9 - 35.3]; 41.1 [35.6 - 46.7];  Partner: 10.0 [6.6 - 13.4];  Past year prevalence: Mixed types of violence: Unspecified: 97.0 [95.1 - 98.9];  Workplace: 90.0 [86.6 - 93.4]; 28.1 [23.0 - 33.2]; 43.1 [37.5 - 48.8]  Police: 20.1 [15.5 - 24.6];  Partner: 31.1 [25.9 - 36.4]; | Alcohol use: OR = 2.2;  Drug use: OR = 2.0 | - |
| Latimer et al., 2018 (Poor)  [229] | Sex working assessed as "risk factor";  Inclusion criteria: None reported.  Exclusion criteria: None reported. | Cross sectional study | HICs: Australia | December 2017 - February 2018 | 179 | Lifetime prevalence: Sexual violence only: Unspecified: 50.8 [43.5 - 58.2] | - | - |
| Reed et al., 2022, M (Good)  [230] | Sex workers addressed only;  Inclusion criteria: Cisgender women aged ≥18 years,  assigned female at birth,  who reported exchanging sex for money or goods in the past month,  agreed to treatment for any STIs, resided in Tijuana,  and had no plans to move in the next 18 months.  Exclusion criteria: None reported. | Cohort study | LMICs: Region of the Americas: Mexico | 2014 - 2016 | 228 | 6-months prevalence: Sexual violence only: Unspecified: 26.3 [20.6 - 32.0] | - | - |
| Rodríguez et al., 2014 (Poor)  [231] | Sex workers addressed only;  Inclusion criteria: **I**ndividuals aged 18–65 years  who were engaged in sex work,  able to read and write,  and accepted the invitation to participate.  Exclusion criteria: None reported. | Cross sectional study | LMICs: Region of the Americas: Mexico | 2010 - 2010 | 103 | Lifetime prevalence: Sexual violence only: Unspecified: 13.6 [7.0 - 20.2];  Lifetime prevalence: Mixed types of violence: Unspecified: 50.5 [40.8 - 60.1];  Workplace: 77.7 [69.6 - 85.7]; 30.1 [21.2 - 39.0]; 97.1 [93.8 - 100.3];  Police: 25.2 [16.9 - 33.6]; | - | - |
| Surís et al., 2022 (Fair)  [232] | Sex working assessed as "risk factor";  Inclusion criteria: Young people aged 24–26 years  residing in Switzerland.  Exclusion criteria: None reported. | Cross sectional study | HICs: Switzerland | 2017 - | 88 | Lifetime prevalence: Sexual violence only: Unspecified: 34.1 [24.2 - 44.0] | - | - |

Note. *N* = number of included sex workers in sample, CI = Confidence Interval, OR = Odds Ratio, PR = Prevalence Ratio, RR = Risk Ratio, M = was also quantitatively analysed (mixed-method study)

## Table A5: Included quantitative studies from countries with not specified legislative models

| First Author, year  (Study quality)  [Publications used] | Targeted population | Study type | Region / country | Data collection time period | N | Sexual violence prevalences  % [95 %CI] | Mental health outcomes  OR/PR/RR  [95 % CI] | Sexual health outcomes  OR/PR/RR  [95 % CI] |
| --- | --- | --- | --- | --- | --- | --- | --- | --- |
| International Committee on the Rights of Sex Workers in Europe (ICRSE), 2020 (Fair)  [233] | Sex workers addressed only;  Inclusion criteria: Migrant sex workers  who were already connected to sex worker organizations.  Exclusion criteria: None reported. | Mixed-method research  Excluded from qualitative meta-aggregation due to mixed sample with participants with forced entry into sex work | HICs: Austria, Belgium, France, Greece, Hungary, Ireland, Italy, the Netherlands, Romania, and the United Kingdom | June 2019 - December 2019 | 47 | Lifetime prevalence: Sexual violence only: Unspecified: 25.5 [13.1 - 38.0];  Workplace: 14.9 [4.7 - 25.1];  Lifetime prevalence: Forced entry into sex work: 19.1 [7.9 - 30.4] | - | - |
| European Sex Workers' Rights Alliance (ESWA), 2024, M (Poor)  [234, 235] | Sex workers addressed only;  Inclusion criteria: None reported.  Exclusion criteria: None reported. | Cross sectional study | Unspecified: Armenia, Belgium, France, Greece, the Netherlands, North Macedonia, Poland, Spain, Sweden, Switzerland, United Kingdom | - | 199 | Lifetime prevalence: Sexual violence only: Police: 10.1 [5.9 - 14.2]; 26.6 [20.5 - 32.8]; 2.5 [0.3 - 4.7];  Lifetime prevalence: Mixed types of violence: Police: 76.9 [71.0 - 82.7] | - | - |
| Beletsky et al., 2013 (Good)  [236–244] | Sex workers addressed only;  Inclusion criteria: Sex workers aged ≥18 years  who had unprotected vaginal or anal sex with a male client in the past month,  shared injection paraphernalia or injected illicit drugs in the past month,  could speak Spanish or English,  were able to provide informed consent,  had no plans to permanently move out of the city in the next 18 months,  and agreed to accept free STI treatment.  Exclusion criteria: None reported. | behavioral intervention study | LMICs: Region of the Americas: Mexico | October 2008 - October 2009 | 624 | Lifetime prevalence: Sexual violence only: Unspecified: 50.2 [46.2 - 54.1];  Workplace: 22.4 [19.1 - 25.7];  6-months prevalence: Sexual violence only: Workplace: 22.3 [19.0 - 25.5]; 5.5 [3.7 - 7.3]  Police: 17.0 [14.0 - 19.9]; 32.5 [28.9 - 36.2] | - | HIV: OR = 1.1 [0.5 - 2.5]; OR = 1.1 [0.3 - 5.0];  Miscarriage: OR = 1.9 [1.3 - 2.7];  Other STI than HIV: PR = 1.0 [0.8 - 1.3]; PR = 1.0 [0.6 - 1.4];  Miscarriage: aOR = 1.7 [1.1 - 2.6] |
| Cepeda & Nowotny, 2014, M ()  [245] | Sex workers addressed only;  Inclusion criteria: Female sex workers  aged >18 years  working in one of the selected venues and self-reporting participation in exchanging sex for money or drugs in that venue.  Exclusion criteria: None reported. | Qualitative research | LMICs: Region of the Americas: Mexico | - | 109 | Lifetime prevalence: Sexual violence only: Unspecified: 59.6 [50.4 - 68.8];  Lifetime prevalence: Mixed types of violence: Unspecified: 60.6 [51.4 - 69.7] | - | - |
| Geller et al., 2020 (Good)  [246] | Sex working assessed as "risk factor";  Inclusion criteria: Individuals aged ≥18 years  with a recent STI diagnosis at the index and follow-up visits,  who provided complete data on recent (past 6-month) gender-based violence (GBV) experience,  and contributed at least one visit pair between 1994 and 2018 (N = 3,461 participants, 59,239 visit pairs).  Exclusion criteria: Individuals with no recent STI diagnosis at the index visit (n = 940),  no recent sexual activity at the second visit (n = 13,324),  or HIV seroconversion over the course of a visit pair (n = 5). | Cohort study | HICs: United States | 1994 - 2018 | 98 | 6-months prevalence: Mixed types of violence: Unspecified: 34.7 [25.3 - 44.1] | - | - |
| Goldenberg et al., 2013 (Fair)  [247] | Sex workers addressed only;  Inclusion criteria: Individuals aged ≥18 years  who traded sex in the past 30 days,  reported lifetime use of heroin, cocaine, crack, or methamphetamine,  had a stable partner for at least 6 months with whom they had sex in the past 30 days,  and were able to recruit that partner to participate in the study.  Exclusion criteria: Individuals reporting high levels of current intimate partner violence. | Cross sectional study | LMICs: Region of the Americas: Mexico | May 2010 - September 2011 | 214 | Lifetime prevalence: Sexual violence only: Unspecified: 28.5 [22.5 - 34.6];  Partner: 17.3 [12.2 - 22.4];  Lifetime prevalence: Forced entry into sex work: 14.5 [9.8 - 19.2] | - | - |
| Jain et al., 2020 (Good)  [248] | Sex workers addressed only;  Inclusion criteria: Cisgender women aged ≥18 years  who self-identified as sex workers,  exchanged sex for money, drugs, or other goods in the past month,  engaged in condom-unprotected vaginal or anal sex with a male client in the past month,  were HIV-negative,  owned a cellular phone,  and were willing to receive treatment if STI-positive.  Exclusion criteria: None reported. | Cross sectional study | LMICs: Region of the Americas: Mexico | July 2016 - January 2017 | 295 | Lifetime prevalence: Mixed types of violence: Workplace: 36.3 [30.8 - 41.8] | Alcohol use: OR = 1.7 [1.0 - 2.7];  Drug use: OR = 2.2 [1.3 - 3.6];  Depressive symptoms: OR = 2.1 [1.3 - 3.4] | Other STI than HIV: OR = 1.1 [0.6 - 1.9] |
| Lafort et al., 2017 (Good)  [249, 250] | Sex workers addressed only;  Inclusion criteria: Female sex workers who received money or gifts for sex at least three times in the past six months; aged ≥18 years in Durban and Mombasa, and aged 15–17 years in Mozambique if considered emancipated minors.  Exclusion criteria: Female sex workers younger than 18 years in Durban and Mombasa (except emancipated minors in Mozambique). | Cross sectional study  Mixed-method research  Excluded from qualitative meta-aggregation due to lack of qualitative data | LMICs: South-East Asia Region: Mysore, India | - | 458 | Past year prevalence: Sexual violence only: Unspecified: 7.2 [4.8 - 9.6] | - | - |
|  |  |  | LMICs: African Region: Tete, Mozambique | - | 308 | Past year prevalence: Sexual violence only: Unspecified: 13.6 [9.5 - 17.8] | - | - |
|  |  |  | LMICs: African Region: Durban, South Africa | - | 400 | Past year prevalence: Sexual violence only: Unspecified: 36.4 [31.6 - 41.1] | - | - |
|  |  |  | LMICs: African Region: Mombasa, Kenya | - | 400 | Past year prevalence: Sexual violence only: Unspecified: 14.8 [11.3 - 18.3] | - | - |
| Lyons et al., 2020 (Good)  [251] | Sex workers addressed only;  Inclusion criteria: Individuals aged ≥18 years  who attributed more than half of their income in the past 12 months to selling sex,  self-reported female sex assigned at birth,  and were capable of providing informed consent.    Exclusion criteria: None reported. | Cross sectional study | LMICs: African Region: Burkina Faso, Senegal, Cote d'Ivoire, Guinea-Bissau, The Gambia, Togo, Cameroon, Lesotho, Kingdom of eSwatini, South Africa | January 2013 - May 2018 | 7259 | Lifetime prevalence: Sexual violence only: Unspecified: 30.5 [29.4 - 31.6] | - | HIV: OR = 1.3 [1.2 - 1.4];  aOR = 1.3 [1.1 - 1.5] |
| Olakunde et al., 2025 (Fair)  [252] | Sex working assessed as "risk factor";  Inclusion criteria: None reported.  Exclusion criteria: None reported. | Cross sectional study | LMICs: African Region: Nigeria | 2020 - 2020 | 1147 | Past year prevalence: Sexual violence only: Unspecified: 29.6 [26.9 - 32.2] | - | - |
| Oldenburg et al., 2015 (Good)  [253] | Sex working assessed as "risk factor";  Inclusion criteria: None reported.  Exclusion criteria: Individuals whose current gender identity was transgender or differed from their sex assigned at birth. | Cross sectional study | LMICs: Region of the Americas: Argentina, Bolivia, Brazil, Chile, Colombia, Costa Rica, Ecuador, El Salvador, Guatemala, Honduras, Mexico, Nicaragua, Panama, Paraguay, Peru, Uruguay, and Venezuela | 2012 - | 1732 | Lifetime prevalence: Mixed types of violence: Partner: 46.7 [44.3 - 49.0] | - | - |
| Semple et al., 2015 (Good)  [254] | Sex workers addressed only;  Inclusion criteria: Self-identified female sex workers (FSWs) aged ≥18 years,  who reported trading sex for money, drugs, shelter, or other material benefits in the past 2 months,  had unprotected vaginal or anal sex with a client at least once in that period,  and tested HIV-negative at baseline.  Exclusion criteria: None reported. | Cross sectional study | LMICs: Region of the Americas: Mexico | June 2011 - December 2013 | 1089 | 6-months prevalence: Sexual violence only: Workplace: 11.7 [9.8 - 13.6];  6-months prevalence: Mixed types of violence: Workplace: 22.6 [20.1 - 25.1] | Alcohol use: aOR = 1.0 [0.9 - 1.1];  Drug use: aOR = 2.9 [1.5 - 5.8] | - |
| Silverman et al., 2015 (Fair)  [255] | Sex workers addressed only;  Inclusion criteria: Female sex workers aged ≥18 years.  Exclusion criteria: None reported. | Cross sectional study | LMICs: Region of the Americas: Mexico | March 2013 - January 2014 | 603 | Lifetime prevalence: Sexual violence only: Workplace: 10.8 [8.3 - 13.3] | - | - |
| Zalla et al., 2019 (Good)  [256] | Sex workers included as subgroup;  Inclusion criteria: Individuals aged ≥15 years  who, in the past 3 months, reported any of the following:  sex with ≥3 different partners,  anal sex with anyone,  or meeting a new sexual partner at a public festival;  designed to capture all men who have sex with men (MSM) and female sex workers (FSWs) without requiring self-identification as a key population.  Exclusion criteria: None reported. | Cross sectional study | LMICs: Region of the Americas: Haiti | November 2011 - | 990 | Lifetime prevalence: Sexual violence only: Unspecified: 54.8 [51.7 - 57.9] | - | - |

Note. *N* = number of included sex workers in sample, CI = Confidence Interval, OR = Odds Ratio, PR = Prevalence Ratio, RR = Risk Ratio, M = was also quantitatively analysed (mixed-method study)

Studies included in meta-aggregation

## Table B1: Included qualitative studies from countries with full criminalisation

| First Author, year [Publications used] | Targeted population | Region / Country | Data collection time period | N | Study aim |
| --- | --- | --- | --- | --- | --- |
| Tocci, 2024  [257] | Sex workers addressed only; Inclusion criteria: Individuals aged ≥18 years who self-identify as current or former sex workers, have experienced mental health concerns while engaged in sex work, live in the United States, and can communicate in spoken English or American Sign Language (ASL) using a telecommunication device for the deaf (TDD).  Exclusion criteria: None reported. | HICs: United States | February 2023 - May 2023 | 10 | To explore the lived experiences of individuals in sex work in managing mental health concerns, including help-seeking behaviors and perceived barriers and facilitators to care, and to develop a participant-informed theoretical model to guide safe, accessible, and equitable mental health services for this population. |
| Antwi et al., 2023  [258] | Sex workers addressed only;  Inclusion criteria: Female commercial sex workers who provided verbal consent.  Exclusion criteria: Individuals aged <18 years, with a mental diagnosis or cognitive delay, male gender, or for whom a linguistically appropriate translator could not be obtained. | LMICs: African Region: Ghana | May 2012 - July 2012 | 19 | To identify and analyze occupational health and safety risks among West African female commercial sex workers in the Greater Accra region using qualitative methods, with the aim of informing public health interventions and occupational health policies for this population. |
| Bazzi et al., 2019  [259] | Sex workers addressed only;  Inclusion criteria: Individuals aged ≥18 years who traded sex in the past month for money, alcohol, drugs, or other material items, reported past-month “problematic” alcohol use (binge drinking ≥5 drinks or being drunk most/all of the time when drinking) or any injection/non-injection drug use, and experienced physical or sexual violence from intimate partners, clients, or police in the past year.  Exclusion criteria: None reported. | LMICs: African Region: Kenya | 2016 - 2017 | 73 | To examine the co-occurrence of substance use, violence, and HIV (syndemics) and their association with the acceptability and feasibility of HIV pre-exposure prophylaxis among female and male sex workers in Kenya. |
| Beaujolais et al., 2020  [260] | Sex workers addressed only;  Inclusion criteria: Female individuals aged ≥18 years living in Kathmandu.  Exclusion criteria: None reported. | LMICs: South-East Asia Region: Nepal | - | 30 | To explore situational factors associated with client-perpetrated violence against female sex workers in Kathmandu, Nepal. |
| Cange et al., 2017  [261] | Sex workers addressed only;  Inclusion criteria: Individuals aged ≥18 years who reported that more than 50% of their annual income in the past year came from sex work and were able to provide informed consent.  Exclusion criteria: None reported. | LMICs: African Region: Cameroon | - | 100 | To qualitatively examine the role of social cohesion and individual and community resilience strategies in promoting empowerment among sex workers in Cameroon, including strategies related to financial independence, HIV risk mitigation, social capital, and motherhood. |
| Cange et al., 2019, M  [20, 21] | Sex workers addressed only;  Inclusion criteria: Individuals aged ≥18 years,  born female,  who sold sex within the past 12 months with the majority of income derived from sex work,  resided in one of the study sites for at least 3 months,  able to provide informed consent in French or a local language (Mòoré or Dioula),  and (for the quantitative phase) in possession of a valid study coupon.  Exclusion criteria: None reported. | LMICs: African Region: Burkina Faso | January 2013 - July 2013 | 696 | To assess the impact of traumatic experiences on suicidal intentions among female sex workers in Burkina Faso to inform comprehensive, context-specific programming for this population. |
| Decker et al., 2013, M  [25] | Sex workers addressed only;  Inclusion criteria: Women,  including transwomen,  aged ≥18 years who traded sex for drugs, money, or a place to stay within the past three months.  Exclusion criteria: None reported. | HICs: United States | March 2012 - August 2012 | 35 | To describe the types of physical and sexual violence and mistreatment experienced by women involved in sex work in Baltimore by perpetrator type, and to examine the relationship between these experiences and HIV risk. |
| Dewey & St. Germain, 2014  [262] | Sex workers addressed only;  Inclusion criteria: None reported.  Exclusion criteria: None reported. | HICs: United States | - | 50 | To explore how women sex workers define coercion in their daily work and to examine their help-seeking practices in response to coercive experiences. |
| Katumba et al., 2024  [263] | Sex workers addressed only;  Inclusion criteria: Individuals aged ≥18 years who self-identified as sex workers or offered sex for money and spoke Luganda or English.  Exclusion criteria: None reported. | LMICs: African Region: Uganda | September 2022 - October 2022 | 20 | To provide a comprehensive understanding of the contexts in which women sell sex in Kampala, focusing on underrepresented groups, to inform targeted interventions aligned with the UNAIDS “leave no one behind” strategy. |
| Khofi et al., 2025  [264] | Sex workers addressed only;  Inclusion criteria: Women who identified themselves as victims or survivors of intimate partner violence (IPV).  Exclusion criteria: None reported. | LMICs: African Region: South Africa | 2022 - 2023 | 15 | To examine how structural violence, food insecurity, economic hardship, and gender-based violence influence the reproductive autonomy of migrant women engaging in transactional sex in urban South Africa, highlighting systemic barriers to healthcare and survival strategies. |
| Kyriakakis et al., 2024  [265] | Sex workers addressed only;  Inclusion criteria: Women aged ≥18 years engaged in sex work.  Exclusion criteria: None reported. | LMICs: Region of the Americas: Republic of Barbados | - | 30 | To explore the experiences of violence faced by cisgender women engaged in sex work in Barbados while on duty, examining how they exercise agency to create safety and how economic, social, and policy contexts influence their vulnerability, to inform supportive interventions. |
| Levine, 2021  [266] | Sex workers addressed only;  Inclusion criteria: None reported.  Exclusion criteria: None reported. | HICs: United States | 2009 - 2009 | 1 | To provide an autoethnographic account of dungeon labor, examining how a sex worker navigates identity performance and authenticity in interactions with colleagues, managers, and clients. |
| Lim et al., 2015  [267] | Sex workers addressed only;  Inclusion criteria: Individuals aged ≥18 years, born female, who reported sex work as their principal source of income in the past 12 months and could provide informed oral consent in French or English.  Exclusion criteria: None reported. | LMICs: African Region: Cameroon | March 2013 - August 2013 | inconclusive | To qualitatively examine the types and sources of violence experienced by female sex workers in Cameroon, explore their harm reduction and resilience strategies, and gather participant recommendations for interventions addressing violence and HIV risk. |
| Maher et al., 2015  [268] | Sex workers addressed only;  Inclusion criteria: Women aged 15–29 years who reported transactional sex (sex in exchange for money, goods, services, or drugs) in the previous three months and understood spoken Khmer.  Exclusion criteria: None reported. | LMICs: Western Pacific Region: Cambodia | 2009 - 2011 | 80 | To explore how the 2008 Trafficking Law in Phnom Penh affects female sex workers’ vulnerability to HIV and their access to health rights. |
| Marlow et al., 2014  [269] | Sex workers addressed only;  Inclusion criteria: Self-identified sex workers aged ≥18 years who attended a clinic in one of the largest urban slums in Kampala, Uganda, had just received either an induced abortion or post-abortion care, and provided written informed consent.  Exclusion criteria: None reported. | LMICs: African Region: Uganda | - | 9 | To explore the experiences of sex workers accessing induced abortion and post-abortion care at an urban private clinic in Uganda, and to identify strategies to improve service delivery and accessibility for this population. |
| Mbonye et al., 2014  [270] | Sex workers addressed only;  Inclusion criteria: Individuals who self-identified as active sex workers.  Exclusion criteria: None reported. | LMICs: African Region: Uganda | March 2010 - June 2011 | 40 | To qualitatively examine the drivers of alcohol consumption and related risky sexual behavior among female sex workers in Kampala, exploring how past experiences and current work practices shape these behaviors. |
| Nattabi et al., 2025  [271] | Sex workers addressed only;  Inclusion criteria: Individuals aged ≥18 years who reported engagement in unsafe transactional sex (sex act in exchange for pay) and at least one episode of unprotected sex in the past 30 days.  Exclusion criteria: None reported. | LMICs: African Region: Uganda | - | 53 | To explore the processes and factors influencing sex work exit among women, with the goal of informing interventions and programs that support safe and successful transitions out of sex work. |
| Okanlawon et al., 2013  [272] | Sex workers addressed only;  Inclusion criteria: Individuals who had sold sex to another man in the past six months and were actively seeking male paying clients.  Exclusion criteria: Individuals not considered “full-time” sex workers, i.e., those gainfully employed who sold sex only occasionally for leisure or extra income. | LMICs: African Region: Nigeria | 2009 - | 6 | To explore the experiences, social circumstances, vulnerabilities, and sexual health risks of male sex workers in Nigeria, aiming to reduce their vulnerability and address gaps in research beyond epidemiology. |
| Onyango et al., 2015  [273] | Sex workers addressed only;  Inclusion criteria: Young women aged 18–20 years who had been engaged in sex work for at least 2 years.  Exclusion criteria: None reported. | LMICs: African Region: Ghana | August 2011 - October 2011 | 48 | To qualitatively examine the social, economic, structural, and individual vulnerabilities of female adolescents selling sex in Kumasi, Ghana, to inform HIV prevention, harm reduction interventions, and targeted service delivery. |
| Panneh et al., 2022  [274, 275] | Sex workers addressed only;  Inclusion criteria: Individuals aged 18–45 years, not pregnant or breastfeeding, without chronic illnesses (e.g., diabetes, rheumatoid arthritis, asthma, or tuberculosis infection in the past 6 months), who had attended any of the seven SWOP clinics within the 12 months preceding the study.  Exclusion criteria: None reported. | LMICs: African Region: Kenya | January 2019 - January 2021 | 40 | To qualitatively investigate the lifetime mental health experiences, perceived risk factors, and coping strategies of female sex workers in Nairobi, Kenya, providing contextual insights into violence, substance use, and work-related challenges. |
| Pokharel et al., 2024  [80] | Sex working assessed as "risk factor";  Inclusion criteria: Transgender women  aged ≥18 years,  residing in Kathmandu Valley during data collection.  Exclusion criteria: Transgender women  aged <18 years,  not residing in Kathmandu Valley during data collection,  or unwilling to participate. | LMICs: South-East Asia Region: Nepal | July 2022 - December 2022 | 104 | To explore the factors contributing to commercial sex work among transgender women in Nepal and to examine their experiences, including stigma associated with sex work, using a mixed-methods approach. |
| Preble et al., 2021  [276] | Sex workers addressed only;  Inclusion criteria: Individuals who had ever sold or traded sex for money or other goods, regardless of sex trade market or whether they had exited sex work.  Exclusion criteria: Indirect sex workers (e.g., strippers, exotic dancers, phone sex workers), individuals who never exited sex work, those who did not explain risks or impacts of exiting in detail, and male or gender non-conforming sex workers. | HICs: United States | January 2012 - September 2014 | 19 | To examine how sex workers perceive and prioritize risks in indoor versus outdoor work settings, and how these perceptions influence behaviors, including exiting sex work, with implications for policies, harm reduction interventions, and future research. |
| Shepp 2023  [277] | Sex workers addressed only;  Inclusion criteria: Individuals aged ≥18 years who had engaged in sex work within the last five years and had experienced gender-based violence (e.g., domestic violence, sexual violence, harassment) for which they accessed gender-based violence services in the last five years.  Exclusion criteria: Individuals who never engaged in sex work, aged <18 years, or who had not experienced gender-based violence and/or had not accessed gender-based violence services. | HICs: United States | Fall 2021 - January 2022 | 34 | To explore the experiences of sex workers who are survivors of gender-based violence in accessing and navigating formal and informal support services, including the impact of anti-trafficking initiatives, barriers and facilitators to service use, and the role of informal support networks. |
| Sherman et al., 2015  [278] | Sex workers addressed only;  Inclusion criteria: Individuals aged ≥18 years who had traded sex for drugs, money, or a place to stay within the three months prior to being interviewed.  Exclusion criteria: None reported. | HICs: United States | March 2012 - August 2012 | 35 | To examine the nature of interactions between police and street- and venue-based female sex workers in Baltimore, considering both professional and personal contexts and the influence of broader structural factors such as criminalization, stigma, and sexism. |
| Siegel et al., 2023  [279, 280] | Sex workers addressed only;  Inclusion criteria: Individuals aged 18–45 years, assigned male at birth and currently identifying as male, genderqueer, or non-binary, self-identifying as Black/African-American, White/Caucasian, or Hispanic/Latino, fluent in English, residing in specified U.S. cities, who reported engaging in exchange sex with at least two different male partners in the prior three months, had anal sex with at least one of those partners, and met at least one partner via a hookup/dating app or website, and had never tested positive for HIV.  Exclusion criteria: None reported. | HICs: United States | October 2018 - April 2020 | 180 | To explore experiences of physical and sexual violence, threats, and robbery among male sex workers in the United States who primarily meet clients online and work independently. |
| Simmons & Syvertsen, 2022  [281] | Sex workers addressed only;  Inclusion criteria: None reported.  Exclusion criteria: None reported. | LMICs: African Region: Kenya | August 2016 - December 2016 | 45 | To ethnographically examine the experiences of Kenyan women sex workers within the context of global antiblackness, with a focus on informing violence prevention in global health while critically reflecting on the framing of global health interventions. |

Note. *N* = number of included sex workers in sample, M = was also quantitatively analysed (mixed-method study)

## Table B2: Included qualitative studies from countries with criminalisation of purchase of sex

| First Author, year [Publications used] | Targeted population | Region / Country | Data collection time period | N | Study aim |
| --- | --- | --- | --- | --- | --- |
| Benner, 2022  [282] | Sex workers addressed only;  Inclusion criteria: Individuals aged ≥19 years who had serviced clients on at least 15 occasions in one year, were legally entitled to work in Canada, and were HIV seronegative or uncertain of their HIV status at the time of the interviews.  Exclusion criteria: None reported. | HICs: Canada | July 2015 - August 2015 | 9 | To explore the following three research questions: (1) How are social contexts and conditions understood to mediate HIV vulnerabilities among survival sex workers living without HIV when offering commercial services to clients? (2) What are the social contexts and conditions under which survival sex workers perceive greater HIV vulnerabilities with their non-commercial, intimate partners? (3) What strategies do sex workers employ to maintain and support their sexual health, and how are these strategies understood? |
| Bungay & Guta, 2018  [283] | Sex workers addressed only;  Inclusion criteria: Individuals aged ≥19 years who provided consensual sexual services for money in an indoor setting in the previous 6 months.  Exclusion criteria: None reported. | HICs: Canada | 2014 - 2016 | 85 | To examine Canadian indoor sex workers’ strategies for preventing workplace violence and the socio-structural factors shaping these experiences, using Canadian data to advance understanding of health and safety in indoor sex work and provide recommendations for integrated, occupation-based public health responses. |
| Dawthorne 2023  [284] | Sex workers addressed only;  Inclusion criteria: Men aged ≥18 years who had provided intimate services, including selling sex (e.g., strippers, hookers, prostitutes, escorts, rentboys).  Exclusion criteria: None reported. | HICs: Canada | 2015 - 2017 | 43 | To provide a nuanced view of male sex work, highlighting structural and experiential differences among sex workers, their clients, and managers, following a polymorphous paradigm in the field of sex work   To highlight the complexity and plurality of sex workers’ lives and actions, emphasizing their unique knowledge of issues affecting them, which should inform health and social policies and practices (at all government levels) |
| Krüsi et al., 2016  [285, 286] | Sex workers addressed only;  Inclusion criteria: Individuals aged ≥18 years currently engaged in sex work (exchanged sex for money in the previous month) in the City of Vancouver, identifying as cisgender or transgender women.  Exclusion criteria: None reported. | HICs: Canada | January 2013 - November 2013 | 31 | To explore how structural vulnerability and stigma intersect with evolving policing strategies to shape street-based sex workers’ civic rights, experiences of violence, and negotiation of sexual risk reduction. To highlight the specific ways in which sex work-related stigma normalizes violence and continuously displaces sex workers from the neoliberal urban landscape, not only through police actions but also via neighbourhood watch groups, private security, and other mechanisms of urban gentrification. |
| Kuosmanen & de Cabo, 2021, M  [153] | Sex workers addressed only;  Inclusion criteria: Men who reported that at some point in their life they had sold sex  and who self-identified as men.  Exclusion criteria: Transgender respondents. | HICs: Sweden | 2011 - 2015 | 156 | To investigate how men who sell sex to men perceive risks in their work, their experiences of denigration, threats, and violence from clients, and the self-defense strategies they use to protect themselves. |
| Lyons et al., 2017  [287] | Sex workers addressed only;  Inclusion criteria: Individuals aged ≥14 years who had ever exchanged sex for money, resided in the Greater Vancouver area, and identified as having a gender identity or expression different from their sex assigned at birth.  Exclusion criteria: None reported. | HICs: Canada | June 2012 - May 2013 | 33 | To explore the lived experiences of violence among trans sex workers in Canada and the social-structural contexts that shape these experiences. |
| Yaakobovitch et al., 2024  [288] | Sex workers addressed only;  Inclusion criteria: Not reported.  Exclusion criteria: Female participants. | HICs: Israel | - | 27 | To focus on the lived experiences of amateur pornography male actors, addressing the under-researched amateur pornography genre, and explores their motivations, challenges, and strategies for coping with the specific difficulties of this work. |

Note. *N* = number of included sex workers in sample, M = was also quantitatively analysed (mixed-method study)

## Table B3: Included qualitative studies from countries with partial criminalisation

| First Author, year [Publications used] | Targeted population | Region / Country | Data collection time period | N | Study aim |
| --- | --- | --- | --- | --- | --- |
| Aborisade 2019  [289] | Sex workers addressed only;  Inclusion criteria: Female street-based commercial sex workers operating in various locations within the city of Ibadan.  Exclusion criteria: Brothel-based sex workers. | LMICs: African Region: Nigeria | October 2015 - March 2016 | 56 | To empirically assess human rights abuses by police against street-based sex workers in Nigeria, examine their public health implications, and explore sex workers’ strategies for coping with and preventing violent police encounters. |
| Eshetu et al., 2025  [290] | Sex workers addressed only;  Inclusion criteria: Individuals who had worked as sex workers for at least 12 months and had experienced at least one form of violence.  Exclusion criteria: None reported. | LMICs: African Region: Ethiopia | October 2020 - November 2020 | 12 | To explore the violence experienced by commercial sex workers in Gondar City, Northwest Ethiopia, including reasons for engaging in sex work and coping strategies.  To provide evidence to inform policymakers, health advocates, and researchers on protecting sex workers’ rights and addressing violence in this population. |
| Friend 2023  [291] | Sex workers addressed only;  Inclusion criteria: None reported.  Exclusion criteria: None reported. | LMICs: African Region: Senegal | January 2018 - June 2022 | 18 | To assess perceived risks and benefits of virtual sex work, including STI harm reduction, in collaboration with Dakar-based sex workers.  To develop a digital security toolkit to enhance digital privacy, autonomy, and sexual health and rights, incorporating participant perspectives, recommendations, and practical resources for those engaging in VSW or using digital platforms to connect with clients in diverse labor settings. |
| Katz et al., 2015, M  [186] | Sex workers addressed only;  Inclusion criteria: Females aged 18–35 who reported receiving money or goods in exchange for sexual services as a source of income in the last six months; for the quantitative component, participants were also required to be non-pregnant.  Exclusion criteria: None reported. | LMICs: South-East Asia Region: Bangladesh | May 2011 - July 2011 | 677 | To explore the discrepancy between reported widespread condom use and high abortion rates among female sex workers, and to assess whether their broader sexual and reproductive health needs are being met. Conducted with local non-governmental organization partners in Dhaka, the study uses a formative assessment among hotel-based and street-based female sex workers to calculate unmet contraceptive needs, identify additional sexual and reproductive health services, and determine service preferences, aiming to inform interventions that better address their sexual and reproductive health care needs. |
| Kiernan et al., 2016  [292] | Sex workers addressed only;  Inclusion criteria: Self-identified sex workers aged >18 years working in three high-end clubs in Goma frequented by expatriates and well-off Congolese men, including both male and female sex workers.  Exclusion criteria: Sex workers working in smaller boutique stores. | LMICs: African Region: Congo, Democratic Republic of the | July 2013 - July 2013 | 8 | To explore and describe the experiences of urban sex workers in the eastern Democratic Republic of the Congo, focusing on areas of vulnerability, including exposure to violence, barriers to accessing medical care, and the use of local resources. |
| Mashumba 2024  [293] | Sex workers addressed only;  Inclusion criteria: None reported.  Exclusion criteria: None reported. | LMICs: African Region: Botswana | November 2018 - February 2019 | 20 | To explore male sex workers’ experiences of victimisation in interactions with sex tourists in Botswana, the role of support groups, and proposed industry reforms.  To present their victimisation experiences, associated health risks, and perspectives on support, addressing the limited literature on men who sell sex. |
| Matheson et al., 2022  [294] | Sex workers addressed only;  Inclusion criteria: Individuals aged ≥18 years, male or female, involved in transactional sex, who use drugs, can provide informed consent, and live in Scotland.  Exclusion criteria: None reported. | HICs: United Kingdom | February 2019 - August 2019 | 16 | To identify information, support, and treatment needs related to drug use, sexual health, and bloodborne viruses risk behaviors among people involved in transactional sex, aiming to inform a gender-aware, rights-based approach to services for this highly stigmatized and underserved population. |
| Nichols 2014  [295] | Sex workers addressed only;  Inclusion criteria: Male-bodied individuals in Sri Lanka who worked in the sex industry and embraced a feminine gender identity.  Exclusion criteria: None reported. | LMICs: South-East Asia Region: Sri Lanka | - | 24 | Using a case study of transgender sex workers in Sri Lanka to illustrate how gender and sexual orientation intersect, producing unique victimization and exposing transgender sex workers to police abuses in a context where only feminine-identified gay men are culturally labeled as homosexual. |
| Panchanadeswaran et al., 2024  [296] | Sex workers addressed only;  Inclusion criteria: Cisgender women aged ≥30 years who had exchanged sex for cash or kind in the previous 3 months, solicited clients in streets or public venues (e.g., cinema halls, bus/railway stations, hotels/lodges) or independently through brokers or informal social networks, and provided sexual services in public places or in their own or others’ homes.  Exclusion criteria: None reported. | LMICs: South-East Asia Region: India | - | 39 | To explore the lived experiences of older female sex workers in India, examining their experiences within sex work and beyond as they age, contributing to the sparse literature on this population. |
| Roman 2021  [297] | Sex workers addressed only;  Inclusion criteria: Women currently working as jineteras (sex workers) in Havana, Cuba.  Exclusion criteria: None reported. | LMICs: Region of the Americas: Cuba | - | 7 | To investigate the lives of jineteras, including challenges, exploitation, socio-economic conditions, structural violence, sex tourism, health, safety, and security. The study is guided by four research questions: (1) What are the life stories of jineteras? (2) What kinds of conflicts do jineteras experience, and how do they manage them? (3) What are the perceived lived experiences and perceptions of jineteras? (4) What are the main reasons jineteras engage in jineterismo? |
| Ryan & McGarry, 2022  [298] | Sex workers addressed only;  Inclusion criteria: None reported.  Exclusion criteria: None reported. | HICs: Ireland | June 2019 - May 2020 | 21 | To explore strategies sex workers use to manage information about stigma, including with healthcare providers, and the emotional and physical costs of these strategies—such as isolation, anxiety, and low self-esteem—within Ireland’s 2017 legal framework criminalising the purchase of sex while maintaining offences against ancillary activities  To examine sex workers’ encounters with healthcare providers through the lens of structural violence, which includes assaults on self-respect and personhood, and frames the silencing of marginalized women’s healthcare experiences as a form of structural violence |
| Smaniotto Gehlen et al., 2018  [299] | Sex workers addressed only;  Inclusion criteria: Individuals aged ≥18 years who were present in the house at the time of data collection.  Exclusion criteria: None reported. | LMICs: Region of the Americas: Brazil | September 2012 - February 2013 | 8 | Guided by the question, “What are the situations of individual vulnerability to violence experienced by sex workers?” the study aims to describe the specific situations of vulnerability faced by female sex workers. |
| Twizelimana & Muula, 2015  [300] | Sex workers addressed only;  Inclusion criteria: Individuals aged 15–50 years.  Exclusion criteria: None reported. | LMICs: African Region: Malawi | 2013 - 2014 | 45 | To assess HIV and AIDS risk perception and self-efficacy to prevent infection among female sex workers in semi-urban Blantyre (Lunzu), Malawi, aiming to inform strategies that promote sexual and overall health for sex workers, their clients, and the wider community connected through sexual networks. |
| Zarhin & Fox, 2017  [301] | Sex workers addressed only;  Inclusion criteria: None reported.  Exclusion criteria: None reported. | HICs: Israel | October 2005 - May 2006 | 8 | To draw on observations and interviews with street-based sex workers in Israel to show how the lived experience of stigma alters their cognitive expectations, highlighting how stigma acts as a transformative experience with both material and cognitive consequences.  To examine how sex workers discuss their reasons for staying in sex work and how this relates to experiences of stigma, extending prior research by exploring not only coping strategies but also how stigma shapes actions and alters cognitive expectations. |

Note. *N* = number of included sex workers in sample, M = was also quantitatively analysed (mixed-method study)

## Table B4: Included qualitative studies from countries with regulatory models

| First Author, year [Publications used] | Targeted population | Region / Country | Data collection time period | N | Study aim |
| --- | --- | --- | --- | --- | --- |
| Kloek & Dijkstra, 2018, M  [228] | Sex workers addressed only;  Inclusion criteria: Licensed or non-licensed sex workers in the Netherlands  with real-life client contact, regardless of the type of sex work performed,  able to complete the questionnaire in Dutch, English, or Spanish.  Exclusion criteria: Sex workers working exclusively via webcam or telephone,  and those not sufficiently fluent in Dutch, English, or Spanish. | HICs: Netherlands | January 2017 - September 2017 | 299 | To examine how sex workers in the Netherlands defined and experienced violence, including its forms and extent, associated risk factors and perpetrators, and the role of police, legislation, and policy responses. |
| Reed et al., 2022, M  [230] | Sex workers addressed only;  Inclusion criteria: Cisgender women aged ≥18 years,  assigned female at birth,  who reported exchanging sex for money or goods in the past month,  agreed to treatment for any STIs, resided in Tijuana,  and had no plans to move in the next 18 months.  Exclusion criteria: None reported. | LMICs: Region of the Americas: Mexico | 2014 - 2016 | 228 | To assess associations between economic vulnerability, experiences of physical and sexual violence, and other HIV risk factors among female sex workers in Tijuana, Mexico, using quantitative and qualitative data to inform HIV prevention strategies. |

Note. *N* = number of included sex workers in sample, M = was also quantitatively analysed (mixed-method study)

## Table B5: Included qualitative studies from countries with full decriminalisation

| First Author, year [Publications used] | Targeted population | Region / Country | Data collection time period | N | Study aim |
| --- | --- | --- | --- | --- | --- |
| Armstrong 2016  [302] | Sex workers included as subgroup;  Inclusion criteria: None reported.  Exclusion criteria: None reported. | HICs: New Zealand | 2008 - 2011 | 28 | To examine how female street-based sex workers perceive and manage street harassment, and to assess the significance of these experiences within a decriminalized legal context, with particular attention to the limitations of decriminalization in improving the treatment of sex workers in public spaces. |

Note. *N* = number of included sex workers in sample, M = was also quantitatively analysed (mixed-method study)

## Table B6: Included qualitative studies from countries with not specified legislative models

| First Author, year [Publications used] | Targeted population | Region / Country | Data collection time period | N | Study aim |
| --- | --- | --- | --- | --- | --- |
| Crago 2015  [303] | Sex workers addressed only;  Inclusion criteria: None reported.  Exclusion criteria: None reported. | Unspecified: Albania, Bosnia and Herzegovina, Bulgaria, Hungary, Kazakhstan, Kyrgyzstan, Montenegro, Macedonia, Poland, Romania, Russia, Serbia, Slovakia, Turkey, Ukraine | 2015 - | 320 | To report findings from community-led research across sixteen countries of the region, examining daily violence against sex workers by police and clients, its effects on HIV risk reduction and working conditions, how policing causes displacement, and the barriers, facilitators, and dynamics affecting reporting, redress, and access to justice. |
| European Sex Workers’ Rights Alliance (ESWA), 2023  [304] | Sex workers addressed only;  Inclusion criteria: Current or former sex workers of any gender, age, sexual orientation, HIV status, drug use status, migration status, geographic location (urban or rural), disability status, or history of detention/incarceration, based in Europe.  Exclusion criteria: None reported. | HICs: Belgium, Finland, France, Germany, Italy, Lithuania, Poland, Russia, Spain, Sweden, The Netherlands, Turkey, United Kingdom | - | 70 | To explore sex workers’ experiences of stigma and discrimination in healthcare settings across Europe, aiming to inform interventions to reduce these barriers and improve access to appropriate, high-quality healthcare services. |
| European Sex Workers’ Rights Alliance (ESWA), 2024  [234, 235] | Sex workers addressed only;  Inclusion criteria: None reported.  Exclusion criteria: None reported. | Unspecified: Armenia, Belgium, France, Greece, the Netherlands, North Macedonia, Poland, Spain, Sweden, Switzerland, United Kingdom | - | 199 | To examine the impact of policing on sex workers’ access to justice across eleven European countries and four legal models of sex work, exploring how law enforcement practices, working conditions, and demographic characteristics—including migration status, sexuality, and gender—shape barriers to justice for sex workers as victims and alleged offenders. |
| European Sex Workers’ Rights Alliance (ESWA), 2025  [305] | Sex workers addressed only;  Inclusion criteria: None reported.  Exclusion criteria: None reported. | HICs: Greece, Ireland, Italy, Netherlands, United Kingdom | - | 41 | To provide a conceptual framework, empirical evidence, and policy recommendations to enhance understanding of, and responses to, the housing challenges faced by migrant sex workers across Europe.  To examine migration, sex work, and housing across five European countries by reviewing legislation, policies, and research, presenting primary data collected by ESWA member organisations, and outlining methodology and key findings.  Building on previous findings to explore good practices in addressing migration, sex work, and housing challenges, emphasizing self-management and self-organisation initiatives, and concludes with actionable recommendations for policy and practice.  To assess housing challenges faced by migrant sex workers across five European countries, highlighting how different legal, social, and policy contexts related to sex work, migration, and housing influence their ability to secure stable, adequate housing. |
| Cepeda & Nowotny, 2014, M  [245] | Sex workers addressed only;  Inclusion criteria: Female sex workers  aged >18 years  working in one of the selected venues and self-reporting participation in exchanging sex for money or drugs in that venue.  Exclusion criteria: None reported. | LMICs: Region of the Americas: Mexico | - | 109 | To describe the daily violence experienced by female sex workers in Mexico from clients, bar owners, other sex workers, and police.  To explore how venue, geographic, and prostitution area contexts, along with broader gendered social structures, shape victimization experiences. |
| Meiliana 2023  [306] | Sex workers addressed only;  Inclusion criteria: None reported.  Exclusion criteria: None reported. | LMICs: Western Pacific Region: Indonesia | - | 5 | To examine the Ronggeng culture and the negative stigma associated with this traditional art form, and to highlight the cultural violence experienced by women participating in Ronggeng practices. |
| Nelson 2020  [307] | Sex workers addressed only;  Inclusion criteria: Current cisgender women sex workers who exchanged sex for money in the previous month and solicited and serviced clients on the streets.  Exclusion criteria: None reported. | LMICs: African Region: Nigeria | October 2016 - February 2017 | 27 | To explore structural inequities, HIV vulnerability, and agency among street-based female sex workers in Nigeria, highlighting how structural violence shapes their experiences and informs legal reforms and HIV prevention efforts. |
| Oselin & Blasyak, 2013  [308] | Sex workers addressed only;  Inclusion criteria: Female street prostitutes.  Exclusion criteria: None reported. | HICs: United States | 2002 - 2006 | 17 | To investigate how female street-based sex workers respond to violence, examining how their circumstances shape the strategies they use and whether these strategies have an internal or external focus. |
| Scorgie et al., 2013  [309, 310] | Sex workers addressed only;  Inclusion criteria: Individuals aged ≥18 years who had at least one client in the past week and were not under the influence of drugs or alcohol at the time of recruitment.  Exclusion criteria: None reported. | LMICs: African Region: Kenya, Uganda, Zimbabwe, South Africa | December 2010 - January 2011 | 136 | To examine female, male, and transgender sex workers’ experiences of accessing public and private healthcare in four countries in East and Southern Africa, identifying barriers to care and strategies for improving services, alongside how sex workers navigate human rights abuses and violence. |
| Spyrelis & Ibisomi, 2022  [311] | Sex workers addressed only;  Inclusion criteria: Female sex workers aged ≥18 years working in the towns targeted by the project.  Exclusion criteria: None reported. | LMICs: African Region: Lesotho, Malawi, Mozambique, South Africa, eSwatini, Zambia | April 2018 - December 2018 | 20 | To explore the sexual and reproductive health awareness, needs, and contexts of female sex workers in towns targeted by the regional “SRHR-HIV Knows No Borders” intervention.  To present a rapid assessment conducted before the “SRHR-HIV Knows No Borders” project in six Southern African countries, providing baseline insights into sex workers’ sexual and reproductive health needs and contexts.  To describe the implementation of the project from 2016 to 2020 in six Southern African Development Community countries, aiming to improve sexual and reproductive health and HIV outcomes among youth, sex workers, and others in high-migration communities affected by mobility-related health risks. |

Note. *N* = number of included sex workers in sample, M = was also quantitatively analysed (mixed-method study)

References

1. Ayamah P, Aheto J, Atuahene KS, Annang DA, Nartey DT, Amuasi SA, Abrefa-Gyan T. Multiple indicators of violence against female sex workers and its associated factors in Ghana: Evidence from the 2015 integrated bio-behavioral surveillance survey cross-sectional study. Health Science Reports 2023. doi:10.1002/hsr2.1243.

2. Baral S, Ketende S, Green JL, Chen P-A, Grosso A, Sithole B, et al. Reconceptualizing the HIV epidemiology and prevention needs of female sex workers (FSW) in Swaziland. PLoS One 2014. doi:10.1371/journal.pone.0115465.

3. Beattie T, Adhiambo W, Kabuti R, Beksinska A, Ngurukiri P, Babu H, et al. The epidemiology of HIV infection among female sex workers in Nairobi, Kenya: A structural determinants and life-course perspective. PLOS Glob Public Health. 2024;4:e0001529. doi:10.1371/journal.pgph.0001529.

4. Beattie T, Kabuti R, Beksinska A, Babu H, Kung’u M, Shah P, et al. Violence across the Life Course and Implications for Intervention Design: Findings from the Maisha Fiti Study with Female Sex Workers in Nairobi, Kenya. Int J Environ Res Public Health 2023. doi:10.3390/ijerph20116046.

5. Shah P, Kabuti R, Beksinska A, Nyariki E, Babu H, Kungu M, et al. Childhood and adolescent factors shaping vulnerability to underage entry into sex work: A quantitative hierarchical analysis of female sex workers in Nairobi, Kenya. BMJ Open 2023. doi:10.1136/bmjopen-2023-078618.

6. Beksinska A, Nyariki E, Kabuti R, Kungu M, Babu H, Shah P, et al. Harmful Alcohol and Drug Use Is Associated with Syndemic Risk Factors among Female Sex Workers in Nairobi, Kenya. Int J Environ Res Public Health 2022. doi:10.3390/ijerph19127294.

7. Beksinska A, Jama Z, Kabuti R, Kungu M, Babu H, Nyariki E, et al. Prevalence and correlates of common mental health problems and recent suicidal thoughts and behaviours among female sex workers in Nairobi, Kenya. BMC Psychiatry 2021. doi:10.1186/s12888-021-03515-5.

8. Panneh M, Ding Q, Kabuti R, Bradley J, Ngurukiri P, Kungu M, et al. Associations of hair cortisol levels with violence, poor mental health, and harmful alcohol and other substance use among female sex workers in Nairobi, Kenya. Discov Ment Health. 2024;4:29. doi:10.1007/s44192-024-00086-1.

9. Beattie T, Pollock J, Kabuti R, Abramsky T, Kung’u M, Babu H, et al. Are violence, harmful alcohol/substance use and poor mental health associated with increased genital inflammation?: A longitudinal cohort study with HIV-negative female sex workers in Nairobi, Kenya. PLOS Glob Public Health. 2024;4:e0003592. doi:10.1371/journal.pgph.0003592.

10. Becker M, Mishra S, Bhattacharjee P, Musyoki H, Tennakoon A, Leung S, et al. Differential Burden of HIV Among Adolescent Girls and Young Women by Places Associated With Sex Work: An Observational Study in Mombasa, Kenya. J Acquir Immune Defic Syndr. 2024;96:121–9. doi:10.1097/QAI.0000000000003412.

11. Berger BO, Grosso A, Adams D, Ketende S, Sithole B, Mabuza XS, et al. The prevalence and correlates of physical and sexual violence affecting female sex workers in Swaziland. J Interpers Violence. 2018;33:2745–66. doi:10.1177/0886260516629385.

12. Bhardwaj A, Comins CA, Guddera V, Mcingana M, Young K, Phetlhu R, et al. Prevalence of depression, syndemic factors and their impact on viral suppression among female sex workers living with HIV in eThekwini, South Africa. BMC Womens Health 2023. doi:10.1186/s12905-023-02392-2.

13. Wang L, Dowdy DW, Comins CA, Young K, Mcingana M, Mulumba N, et al. Health-related quality of life of female sex workers living with HIV in South Africa: a cross-sectional study. Journal of the International AIDS Society 2022. doi:10.1002/jia2.25884.

14. Rock A, Comins C, Mulumba N, Young K, Mcingana M, Guddera V, et al. Antiretroviral Treatment Sharing among Female sex Workers Living with HIV in eThekwini (Durban), South Africa: Drivers and Implications for Treatment Success. Journal of the International Association of Providers of AIDS Care 2022. doi:10.1177/23259582221110820.

15. Bhattacharjee P, McClarty LM, Musyoki H, Anthony J, Kioko J, Kaosa S, et al. Monitoring HIV prevention programme outcomes among key populations in Kenya: Findings from a national survey. PLoS One 2015. doi:10.1371/journal.pone.0137007.

16. Bhattacharjee P, Ma H, Musyoki H, Cheuk E, Isac S, Njiraini M, et al. Prevalence and patterns of gender-based violence across adolescent girls and young women in Mombasa, Kenya. BMC Womens Health 2020. doi:10.1186/s12905-020-01081-8.

17. Bitty-Anderson AM, Bakoubayi AW, Gbeasor-Komlanvi FA, Sadio AJ, Coffie PA, Ekouevi DK. Gynecological health care services utilization and violence among female sex workers in Togo in 2021. Reproductive Health. 2024;21:160. doi:10.1186/s12978‑024‑01887‑x.

18. Blumenthal J, Landovitz R, Jain S, He F, Kofron R, Ellorin E, et al. Pre-Exposure Prophylaxis Perspectives, Sociodemographic Characteristics, and HIV Risk Profiles of Cisgender Women Seeking and Initiating PrEP in a US Demonstration Project. AIDS Patient Care and STDs. 2021;35:481–7. doi:10.1089/apc.2021.0114.

19. Bukenya JN, Wanyenze RK, Barrett G, Hall J, Makumbi F, Guwatudde D. Contraceptive use, prevalence and predictors of pregnancy planning among female sex workers in Uganda: A cross sectional study. BMC Pregnancy and Childbirth 2019. doi:10.1186/s12884-019-2260-4.

20. Cange CW, Wirtz AL, Ky-Zerbo O, Lougue M, Kouanda S, Baral S. Effects of traumatic events on sex workers’ mental health and suicide intentions in Burkina Faso: A trauma-informed approach. Sexual Health. 2019;16:348–57. doi:10.1071/SH17213.

21. Grosso AL, Ketende S, Dam K, Papworth E, Ouedraogo HG, Ky-Zerbo O, Baral S. Structural determinants of health among women who started selling sex as minors in Burkina Faso. J Acquir Immune Defic Syndr. 2015;68 Suppl 2:S162-70. doi:10.1097/QAI.0000000000000447.

22. Chersich MF, Bosire W, King’ola N, Temmerman M, Luchters S. Effects of hazardous and harmful alcohol use on HIV incidence and sexual behaviour: A cohort study of Kenyan female sex workers. Globalization and Health 2014. doi:10.1186/1744-8603-10-22.

23. Luchters S, Richter ML, Bosire W, Nelson G, Kingola N, Zhang X-D, et al. The Contribution of Emotional Partners to Sexual Risk Taking and Violence among Female Sex Workers in Mombasa, Kenya: A Cohort Study. PLoS One 2013. doi:10.1371/journal.pone.0068855.

24. Davis JD, Miles GM. “Strive harder and don’t lose hope”: sexual exploitation of male youth in the sex trade in Manila. International Journal of Sociology and Social Policy. 2020;41:689–706. doi:10.1108/IJSSP-05-2020-0189.

25. Decker MR, Pearson E, Illangasekare SL, Clark E, Sherman SG. Violence against women in sex work and HIV risk implications differ qualitatively by perpetrator. BMC Public Health 2013. doi:10.1186/1471-2458-13-876.

26. Decker MR, Lyons C, Billong SC, Njindam IM, Grosso A, Nunez GT, et al. Gender-based violence against female sex workers in Cameroon: Prevalence and associations with sexual HIV risk and access to health services and justice. Sexually Transmitted Infections. 2016;92:599–604. doi:10.1136/sextrans-2015-052463.

27. Decker MR, Nail JE, Lim S, Footer K, Davis W, Sherman SG. Client and partner violence among urban female exotic dancers and intentions for seeking support and justice. Journal of Urban Health. 2017;94:637–47. doi:10.1007/s11524-017-0195-5.

28. Decker MR, Tomko C, Wingo E, Sawyer A, Peitzmeier S, Glass N, Sherman SG. A brief, trauma-informed intervention increases safety behavior and reduces HIV risk for drug-involved women who trade sex. BMC Public Health 2017. doi:10.1186/s12889-017-4624-x.

29. Peitzmeier SM, Tomko C, Wingo E, Sawyer A, Sherman SG, Glass N, et al. Acceptability of microbicidal vaginal rings and oral pre-exposure prophylaxis for HIV prevention among female sex workers in a high-prevalence US city. AIDS Care - Psychological and Socio-Medical Aspects of AIDS/HIV. 2017;29:1453–7. doi:10.1080/09540121.2017.1300628.

30. Deuba K, Anderson S, Ekström AM, Pandey SR, Shrestha R, Karki DK, Marrone G. Micro-level social and structural factors act synergistically to increase HIV risk among Nepalese female sex workers. International Journal of Infectious Diseases. 2016;49:100–6. doi:10.1016/j.ijid.2016.06.007.

31. Figueroa JP, Cooper CJ, Edwards JK, Byfield L, Eastman S, Hobbs MM, Weir SS. Understanding the high prevalence of HIV and other sexually transmitted infections among socio-economically vulnerable men who have sex with men in jamaica. PLoS One 2015. doi:10.1371/journal.pone.0117686.

32. Gerassi LB, Jonson-Reid M, Plax K, Kaushik G. Trading Sex for Money or Compensation: Prevalence and Associated Characteristics from a Sexually Transmitted Infection (STI) Clinic Sample. Journal of Aggression, Maltreatment and Trauma. 2016;25:909–20. doi:10.1080/10926771.2016.1223245.

33. Giorgio M, Townsend L, Zembe Y, Guttmacher S, Kapadia F, Cheyip M, Mathews C. Social support, sexual violence, and transactional sex among female transnational migrants to South Africa. Am J Public Health. 2016;106:1123–9. doi:10.2105/AJPH.2016.303107.

34. Grosso AL, Bowring AL, Njindam IM, Decker MR, Lyons C, Rao A, et al. Sexually Transmitted Infection Risks and Symptoms Heightened Among Female Sex Workers who Started Selling Sex Before the Age of 18 in Five Cities in Cameroon. AIDS and Behavior 2023. doi:10.1007/s10461-023-04196-9.

35. Abelson A, Lyons C, Decker M, Ketende S, Mfochive Njindam I, Fouda G, et al. Lifetime experiences of gender-based violence, depression and condom use among female sex workers in Cameroon. International Journal of Social Psychiatry. 2019;65:445–57. doi:10.1177/0020764019858646.

36. Hail-Jares K, Chang R, Choi S, Zheng H, He N, Huang ZJ. Intimate-partner and client-initiated violence among female street-based sex workers in China: Does a support network help? PLoS One 2015. doi:10.1371/journal.pone.0139161.

37. Herpai N, Lazarus L, Forget E, Balakireva O, Pavlova D, McClarty L, et al. Exploring the dynamics of workplace typologies for sex workers in Eastern Ukraine. Global Public Health. 2022;17:2034–53. doi:10.1080/17441692.2021.1965180.

38. Hladik W, Baughman AL, Serwadda D, Tappero JW, Kwezi R, Nakato ND, Barker J. Burden and characteristics of HIV infection among female sex workers in Kampala, Uganda - a respondent-driven sampling survey. BMC Public Health 2017. doi:10.1186/s12889-017-4428-z.

39. Hoang TG, Pham MK, Sterk CE, Evans DP, Miedema SS, Yount KM. Prevalence of violence victimisation and poly-victimisation among female sex workers in Haiphong, Viet Nam: A cross-sectional study. Global Public Health. 2024;19:2308709. doi:10.1080/17441692.2024.2308709.

40. Hosseini-Hooshyar S, Mirzazadeh A, Karamouzian M, Sharifi H, Khajehkazemi R, Haghdoost A-A, Shokoohi M. Prevalence and Correlates of Sexual Violence Experienced by Female Sex Workers in Iran: Results from a National HIV Bio-Behavioral Surveillance Survey. Violence Against Women. 2022;28:872–89. doi:10.1177/10778012211008992.

41. Khezri M, Shokoohi M, Karamouzian M, Mirzazadeh A, Ghalekhani N, Gholamypour Z, et al. Induced abortion and associated factors among female sex workers in Iran. EUROPEAN JOURNAL OF CONTRACEPTION AND REPRODUCTIVE HEALTH CARE. 2020;25:434–8. doi:10.1080/13625187.2020.1815007.

42. Karamouzian M, Shokoohi M, Kaplan RL, Noroozi A, Sharifi H, Baral SD, Mirzazadeh A. Characterizing the relationship between incarceration and structural risks among female sex workers in Iran: findings of a nationwide biobehavioral surveillance survey. Annals of Epidemiology. 2019;35:29–34. doi:10.1016/j.annepidem.2019.04.011.

43. Shokoohi M, Karamouzian M, Sharifi H, Rahimi-Movaghar A, Carrico AW, Hosseini Hooshyar S, Mirzazadeh A. Crystal methamphetamine use and its correlates in women engaged in sex work in a developing country setting. Drug and Alcohol Dependence. 2018;185:260–5. doi:10.1016/j.drugalcdep.2017.12.025.

44. Shokoohi M, Karamouzian M, Bauer GR, Sharifi H, Hosseini Hooshyar S, Mirzazadeh A. Drug use patterns and associated factors among female sex workers in Iran. Addictive Behaviors. 2019;90:40–7. doi:10.1016/j.addbeh.2018.09.037.

45. Shokoohi M, Karamouzian M, Dolan K, Sharifi H, Mirzazadeh A. Social and structural determinants of health associated with drug use patterns among female sex workers in Iran: A latent class analysis. International Journal of Drug Policy 2021. doi:10.1016/j.drugpo.2020.102798.

46. Hosseini Divkolaye NS, Khalatbari J, Faramarzi M, Seighali F, Radfar S, ArabKhazaeli A, Burkle FM. Frequency and Factors Associated with Violence Against Female Sex Workers in Tehran, Iran. Sexuality and Culture. 2021;25:1–17. doi:10.1007/s12119-020-09745-1.

47. Jewkes R, Milovanovic M, Otwombe K, Chirwa E, Hlongwane K, Hill N, et al. Intersections of sex work, mental ill-health, ipv and other violence experienced by female sex workers: Findings from a cross-sectional community-centric national study in South Africa. Int J Environ Res Public Health 2021. doi:10.3390/ijerph182211971.

48. Jewkes R, Otwombe K, Dunkle K, Milovanovic M, Hlongwane K, Jaffer M, et al. Sexual IPV and non-partner rape of female sex workers: Findings of a cross-sectional community-centric national study in South Africa. SSM - Mental Health 2021. doi:10.1016/j.ssmmh.2021.100012.

49. Jewkes R, Milovanovic M, Otwombe K, Hlongwane K, Hill N, Mbowane V, et al. Understanding drivers of female sex workers’ experiences of external/enacted and internalised stigma: findings from a cross-sectional community-centric national study in South Africa. Culture, Health and Sexuality. 2023;25:1433–48. doi:10.1080/13691058.2022.2160014.

50. Kassanjee R, Welte A, Otwombe K, Jaffer M, Milovanovic M, Hlongwane K, et al. HIV incidence estimation among female sex workers in South Africa: a multiple methods analysis of cross-sectional survey data. The Lancet HIV. 2022;9:e781-e790. doi:10.1016/S2352-3018(22)00201-6.

51. Milovanovic M, Jewkes R, Otwombe K, Jaffer M, Hopkins K, Hlongwe K, et al. Community-led cross-sectional study of social and employment circumstances, HIV and associated factors amongst female sex workers in South Africa: study protocol. Glob Health Action 2021. doi:10.1080/16549716.2021.1953243.

52. Jiwatram-Negrón T, El-Bassel N. Overlapping intimate partner violence and sex trading among high-risk women: Implications for practice. Women and Health. 2019;59:672–86. doi:10.1080/03630242.2018.1544967.

53. Karamouzian M, Mirzazadeh A, Shokoohi M, Khajehkazemi R, Sedaghat A, Haghdoost AA, Sharifi H. Lifetime abortion of female sex workers in Iran: Findings of a national bio-behavioural survey In 2010. PLoS One 2016. doi:10.1371/journal.pone.0166042.

54. Kibone W, Pebolo PF, Olum R, Okot J, Opee J, Awor S, et al. Prevalence and factors associated with violence against women who exchange sex for money in Gulu City, Northern Uganda: a cross-sectional study. BMJ Public Health. 2025;3:e001486. doi:10.1136/ bmjph-2024-001486.

55. L’Engle KL, Mwarogo P, Kingola N, Sinkele W, Weiner DH. A randomized controlled trial of a brief intervention to reduce alcohol use among female sex workers in Mombasa, Kenya. Journal of Acquired Immune Deficiency Syndromes. 2014;67:446–53. doi:10.1097/QAI.0000000000000335.

56. Parcesepe AM, L’Engle KL, Martin SL, Green S, Suchindran C, Mwarogo P. Early Sex Work Initiation and Violence against Female Sex Workers in Mombasa, Kenya. Journal of Urban Health. 2016;93:1010–26. doi:10.1007/s11524-016-0073-6.

57. Parcesepe AM. Interpersonal violence and HIV sexual risk behaviors: The influence of alcohol harm reduction and early initiation of sex work among female sex workers in Mombasa, Kenya: ProQuest Information & Learning; 2016.

58. Logie CH, Wang Y, Lacombe-Duncan A, Jones N, Ahmed U, Levermore K, et al. Factors associated with sex work involvement among transgender women in Jamaica: a cross-sectional study. Journal of the International AIDS Society 2017. doi:10.7448/IAS.20.1.21422.

59. Logie CH, Wang Y, Lalor P, Williams D, Levermore K. Pre and post-exposure prophylaxis awareness and acceptability among sex workers in Jamaica: A cross-sectional study. AIDS and Behavior. 2021;25:330–43. doi:10.1007/s10461-020-02972-5.

60. Menza TW, Lipira L, Bhattarai A, Leon V-D, Orellana ER. Prevalence and correlates of transactional sex among women of low socioeconomic status in Portland, or. BMC Womens Health 2020. doi:10.1186/s12905-020-01088-1.

61. Mimiaga MJ, Hughto J, Klasko-Foster L, Jin H, Mayer KH, Safren SA, Biello KB. Substance use, mental health problems, and physical and sexual violence additively increase HIV risk between male sex workers and their male clients in Northeastern United States. Journal of Acquired Immune Deficiency Syndromes. 2021;86:305–12. doi:10.1097/QAI.0000000000002563.

62. Mishra S, Thompson LH, Sonia A, Khalid N, Emmanuel F, Blanchard JF. Sexual behaviour, structural vulnerabilities and HIV prevalence among female sex workers in Pakistan. Sexually Transmitted Infections. 2013;89:ii34-ii42. doi:10.1136/sextrans-2012-050776.

63. Mokinu RA, Yonge SA, Lafort Y, Sandfort TGM, Mantell JE, Gichangi PB. Sexual practices, their influencers, and utilization of HIV services among female sex workers in Mombasa County, Kenya. Pan Afr Med J. 2024;47:209. doi:10.11604/pamj.2024.47.209.41775.

64. Montgomery B, Rompalo A, Hughes J, Wang J, Haley D, Soto-Torres L, et al. Violence against women in selected areas of the United States. Am J Public Health. 2015;105:2156–66. doi:10.2105/AJPH.2014.302430.

65. Moradi G, Gouya MM, Amini EE, Ghorbani SS, Akbarpour S, Zareie B, et al. Intentional abortion and its associated factors among female sex workers in Iran: Results from national bio-behavioral surveillance-2020. PLoS One 2022. doi:10.1371/journal.pone.0273732.

66. Muldoon KA, Akello M, Muzaaya G, Simo A, Shoveller J, Shannon K. Policing the epidemic: High burden of workplace violence among female sex workers in conflict-affected northern Uganda. Global Public Health. 2017;12:84–97. doi:10.1080/17441692.2015.1091489.

67. Duff P, Birungi J, Dobrer S, Akello M, Muzaaya G, Shannon K. Social and structural factors increase inconsistent condom use by sex workers’ one-time and regular clients in Northern Uganda. AIDS Care - Psychological and Socio-Medical Aspects of AIDS/HIV. 2018;30:751–9. doi:10.1080/09540121.2017.1394966.

68. Erickson M, Goldenberg SM, Ajok M, Muldoon KA, Muzaaya G, Shannon K. Structural determinants of dual contraceptive use among female sex workers in Gulu, northern Uganda. International Journal of Gynecology and Obstetrics. 2015;131:91–5. doi:10.1016/j.ijgo.2015.04.029.

69. Erickson M, Goldenberg SM, Akello M, Muzaaya G, Nguyen P, Birungi J, Shannon K. Incarceration and exposure to internally displaced persons camps associated with reproductive rights abuses among sex workers in northern Uganda. Journal of Family Planning and Reproductive Health Care. 2017;43:201–9. doi:10.1136/jfprhc-2016-101492.

70. Erickson M, Goldenberg SM, Master A, Muzaaya G, Akello M, Braschel M, et al. Interpersonal and structural contexts of intimate partner violence among female sex workers in conflict-affected northern Uganda. Women & health. 2018;58:759–73. doi:10.1080/03630242.2017.1342742.

71. Goldenberg SM, Muzaaya G, Akello M, Nguyen P, Birungi J, Shannon K. War-Related Abduction and History of Incarceration Linked to High Burden of HIV Among Female Sex Workers in Conflict-Affected Northern Uganda. Journal of acquired immune deficiency syndromes (1999). 2016;73:109–16. doi:10.1097/QAI.0000000000001030.

72. Mutagoma M, Nyirazinyoye L, Sebuhoro D, Riedel DJ, Ntaganira J. Sexual and physical violence and associated factors among female sex workers in Rwanda: a cross-sectional survey. International Journal of STD and AIDS. 2019;30:241–8. doi:10.1177/0956462418800848.

73. Nabayinda J, Namirembe R, Kizito S, Nsubuga E, Nabunya P, Sensoy Bahar O, et al. Correlates of Intimate Partner Violence Among Young Women Engaged in Sex Work in Southern Uganda. J Interpers Violence. 2023;38:10749–70. doi:10.1177/08862605231175908.

74. Nabayinda J, Witte SS, Kizito S, Nanteza F, Nsubuga E, Sensoy Bahar O, et al. The impact of an economic empowerment intervention on intimate partner violence among women engaged in sex work in southern Uganda: A cluster randomized control trial. Soc Sci Med. 2024;348:116846. doi:10.1016/j.socscimed.2024.116846.

75. Ouma S, Ndejjo R, Abbo C, Tumwesigye NM. Client-perpetrated gender-based violence among female sex workers in conflict-affected Northern Uganda: A cross-sectional study. BMJ Open 2021. doi:10.1136/bmjopen-2020-046894.

76. Owen BN, M-Giroux M, Matse S, Mnisi Z, Baral S, Ketende S, et al. Prevalence and correlates of anal intercourse among female sex workers in eSwatini. PLoS One 2020. doi:10.1371/journal.pone.0228849.

77. Parcesepe AM, Toivgoo A, Chang M, Riedel M, Carlson C, DiBennardo R, Witte SS. Physical and sexual violence, childhood sexual abuse and HIV/STI risk behaviour among alcohol-using women engaged in sex work in Mongolia. Global Public Health. 2015;10:88–102. doi:10.1080/17441692.2014.976240.

78. Pedersen CJ, Wickersham JA, Altice FL, Kamarulzaman A, Khoshnood K, Gibson BA, et al. Prevalence and Correlates of Active Amphetamine-Type Stimulant Use Among Female Sex Workers in Malaysia. Frontiers in Psychiatry 2022. doi:10.3389/fpsyt.2022.879479.

79. Pitpitan EV, Kalichman SC, Eaton LA, Watt MH, Sikkema KJ, Skinner D, et al. Men (and Women) as “Sellers” of Sex in Alcohol-Serving Venues in Cape Town, South Africa. Prevention Science. 2014;15:296–308. doi:10.1007/s11121-013-0381-y.

80. Pokharel HS, Myia SD, Chalise A, Paudel S. Exploring commercial sex work among transgender women in Nepal: Contributors and stigma - A mixed-method study. PLoS One. 2024;19:e0314619. doi:10.1371/journal.pone.0314619.

81. Poxon A, Leis M, McDermott M, Kariri A, Kaul R, Kimani J. Emergency departments as under-utilized venues to provide HIV prevention services to female sex workers in Nairobi, Kenya. International Journal of Emergency Medicine 2023. doi:10.1186/s12245-023-00516-x.

82. Reilly KH, Wang J, Zhu Z, Li S, Yang T, Ding G, et al. Inconsistent Condom Use and History of Trauma Among Vietnamese Female Sex Workers in a Chinese Border Region. Sexuality and Culture. 2014;18:119–31. doi:10.1007/s12119-013-9178-1.

83. Rivera AV, Carrillo SA, Braunstein SL. Individual, Environmental, and Early Life Factors Associated With Client-Perpetrated Violence Among Women Who Exchange Sex in New York City, 2016. J Interpers Violence. 2021;36:NP6065-NP6084. doi:10.1177/0886260518811422.

84. Roberts ST, Flaherty BP, Deya R, Masese L, Ngina J, McClelland RS, et al. Patterns of gender-based violence and associations with mental health and HIV risk behavior among female sex workers in Mombasa, Kenya: A latent class analysis. AIDS Behav. 2018;22:3273–86. doi:10.1007/s10461-018-2107-4.

85. Heller M, Roberts ST, Masese L, Ngina J, Chohan N, Chohan V, et al. Gender-Based Violence, Physiological Stress, and Inflammation: A Cross-Sectional Study. Journal of Women’s Health. 2018;27:1152–61. doi:10.1089/jwh.2017.6743.

86. Kwendakwema CN, Sabo MC, Roberts ST, Masese L, McClelland RS, Shafi J, et al. Sexual Violence, Genital Cytokines, and Colposcopy Findings: A Cross-Sectional Study of Women Engaged in Sex Work in Mombasa, Kenya. Sexually Transmitted Diseases. 2025;52:29–36. doi:10.1097/OLQ.0000000000002070.

87. Tolstrup D, Roberts ST, Deya R, Wanje G, Shafi J, James JR, et al. Intimate partner sexual violence is associated with unhealthy alcohol use among Kenyan women engaged in sex work. Drug Alcohol Depend Rep. 2025;14:100315. doi:10.1016/j.dadr.2024.100315.

88. Roberts E, Ma H, Bhattacharjee P, Musyoki HK, Gichangi P, Avery L, et al. Low program access despite high burden of sexual, structural, and reproductive health vulnerabilities among young women who sell sex in Mombasa, Kenya. BMC Public Health 2020. doi:10.1186/s12889-020-08872-6.

89. Schwitters A, Swaminathan M, Serwadda D, Muyonga M, Shiraishi RW, Benech I, et al. Prevalence of Rape and Client-Initiated Gender-Based Violence Among Female Sex Workers: Kampala, Uganda, 2012. AIDS and Behavior. 2015;19:68–76. doi:10.1007/s10461-014-0957-y.

90. Sherman SG, Park JN, Galai N, Allen ST, Huettner SS, Silberzahn BE, et al. Drivers of HIV Infection among Cisgender and Transgender Female Sex Worker Populations in Baltimore City: Results from the SAPPHIRE Study. Journal of Acquired Immune Deficiency Syndromes. 2019;80:513–21. doi:10.1097/QAI.0000000000001959.

91. Decker MR, Park JN, Allen ST, Silberzahn B, Footer K, Huettner S, et al. Inconsistent Condom Use Among Female Sex Workers: Partner-specific Influences of Substance Use, Violence, and Condom Coercion. AIDS and Behavior. 2020;24:762–74. doi:10.1007/s10461-019-02569-7.

92. Decker M, Rouhani S, Park JN, Galai N, Footer K, White R, et al. Incidence and predictors of violence from clients, intimate partners and police in a prospective US-based cohort of women in sex work. Occupational and Environmental Medicine. 2021;78:160–6. doi:10.1136/oemed-2020-106487.

93. Glick JL, Lim S, Beckham SW, Tomko C, Park JN, Sherman SG. Structural vulnerabilities and HIV risk among sexual minority female sex workers (SM-FSW) by identity and behavior in Baltimore, MD. Harm Reduction Journal 2020. doi:10.1186/s12954-020-00383-2.

94. Park JN, Gaydos CA, White RH, Decker MR, Footer K, Galai N, et al. Incidence and Predictors of Chlamydia, Gonorrhea and Trichomonas among a Prospective Cohort of Cisgender Female Sex Workers in Baltimore, Maryland. Sexually Transmitted Diseases. 2019;46:788–94. doi:10.1097/OLQ.0000000000001085.

95. Park JN, Decker MR, Bass JK, Galai N, Tomko C, Jain KM, et al. Cumulative Violence and PTSD Symptom Severity Among Urban Street-Based Female Sex Workers. J Interpers Violence. 2021;36:10383–404. doi:10.1177/0886260519884694.

96. Rouhani S, White RH, Park JN, Sherman SG. High willingness to use overdose prevention sites among female sex workers in Baltimore, Maryland. Drug and Alcohol Dependence 2020. doi:10.1016/j.drugalcdep.2020.108042.

97. Rouhani S, Decker MR, Tomko C, Silberzahn B, Allen ST, Park JN, et al. Resilience among Cisgender and Transgender Women in Street-Based Sex Work in Baltimore, Maryland. Women’s Health Issues. 2021;31:148–56. doi:10.1016/j.whi.2020.11.002.

98. Tomko C, Park JN, Allen ST, Glick J, Galai N, Decker MR, et al. Awareness and interest in HIV pre-exposure prophylaxis among street-based female sex workers: Results from a US context. AIDS Patient Care and STDs. 2019;33:49–57. doi:10.1089/apc.2018.0182.

99. Zemlak JL, White RH, Nestadt DF, Alexander KA, Park JN, Sherman SG. Interpersonal Violence and Contraceptive Method Use by Women Sex Workers. Women’s Health Issues. 2021;31:516–22. doi:10.1016/j.whi.2021.08.001.

100. Rosen JG, Beckham SW, Glick JL, White RH, Park JN, Footer KHA, Sherman SG. Acceptability of Event-Driven and Long-Acting HIV Pre-Exposure Prophylaxis Formulations Among Transgender Women Engaged in Street-Based Sex Work in Baltimore, Maryland. Transgend Health. 2024;9:185–91. doi:10.1089/trgh.2022.0057.

101. Siconolfi D, Storholm ED, Vincent W, Pollack L, Rebchook GM, Huebner DM, et al. Prevalence and Correlates of Sexual Violence Experienced by Young Adult Black Men Who Have Sex with Men. Arch Sex Behav. 2021;50:3621–36. doi:10.1007/s10508-021-02011-x.

102. Sileo KM, Kintu M, Kiene SM. The intersection of intimate partner violence and HIV risk among women engaging in transactional sex in Ugandan fishing villages. AIDS Care - Psychological and Socio-Medical Aspects of AIDS/HIV. 2018;30:444–52. doi:10.1080/09540121.2017.1391985.

103. Simmelink AM, Gichuki CM, Ampt FH, Manguro G, Lim M, Agius P, et al. Assessment of the lifetime prevalence and incidence of induced abortion and correlates among female sex workers in Mombasa, Kenya: a secondary cohort analysis. BMJ Open 2022. doi:10.1136/bmjopen-2021-053218.

104. Stenersen MR, Thomas K, McKee S. Police and Transgender and Gender Diverse People in the United States: A Brief Note on Interaction, Harassment, and Violence. J Interpers Violence. 2022;37:NP23527-NP23540. doi:10.1177/08862605211072161.

105. Stoebenau K, Dunkle K, Willan S, Shai N, Gibbs A. Assessing risk factors and health impacts across different forms of exchange sex among young women in informal settlements in South Africa: A cross-sectional study. Social Science and Medicine 2023. doi:10.1016/j.socscimed.2022.115637.

106. Štulhofer A, Sinković M, Božić J, Baćak V. Victimization and HIV Risks Among Croatian Female Sex Workers: Exploring the Mediation Role of Depressiveness and the Moderation Role of Social Support. Violence Against Women. 2017;23:67–88. doi:10.1177/1077801216636241.

107. Štulhofer A, Laušević D, Božičević I, Baćak V, Mugoša B, Terzić N, Drglin T. HIV risks among female sex workers in croatia and Montenegro. Collegium Antropologicum. 2010;34:881–6.

108. Štulhofer A, Landripet I, Božić J, Božičević I. HIV risks and HIV prevention among female sex workers in two largest urban settings in Croatia, 2008-2014. AIDS Care - Psychological and Socio-Medical Aspects of AIDS/HIV. 2015;27:767–71. doi:10.1080/09540121.2014.996519.

109. Tomko C, Musci RJ, Kaufman MR, Underwood CR, Decker MR, Sherman SG. Mental health and HIV risk differs by co-occurring structural vulnerabilities among women who sell sex. AIDS care. 2023;35:205–14. doi:10.1080/09540121.2022.2121374.

110. Nestadt DF, Tomko C, Schneider KE, Kerrigan D, Decker MR, Sherman SG. Co-occurring Threats to Agency Among Female Sex Workers in Baltimore, Maryland. J Interpers Violence. 2022;37:NP8818-NP8843. doi:10.1177/0886260520978188.

111. Nestadt DF, Schneider KE, Tomko C, Sherman SG. Criminalization and coercion: sexual encounters with police among a longitudinal cohort of women who exchange sex in Baltimore, Maryland. Harm Reduction Journal 2023. doi:10.1186/s12954-023-00738-5.

112. Hendrickson ZM, Tomko C, Galai N, Sisson LN, Glick JL, Sherman SG. A Longitudinal Analysis of Residential Mobility and Experience of Client Violence Among Women Who Exchange Sex in Baltimore. J Interpers Violence. 2023;38:11017–45. doi:10.1177/08862605231178492.

113. Tsai LC, Carlson CE, Aira T, Norcini Pala A, Riedel M, Witte SS. The impact of a microsavings intervention on reducing violence against women engaged in sex work: a randomized controlled study. BMC International Health and Human Rights. 2016;16:1–10. doi:10.1186/s12914-016-0101-3.

114. Urada LA, Strathdee SA, Morisky DE, Schilling RF, Simbulan NP, Estacio LR, Raj A. Sex work and its associations with alcohol and methamphetamine use among female bar and spa workers in the Philippines. Asia-Pacific Journal of Public Health. 2014;26:138–46. doi:10.1177/1010539512471969.

115. Wang M, Lim SH, Gibson BA, Azwa I, Guadamuz TE, Altice FL, et al. Correlates of newly diagnosed HIV infection among cisgender women sex workers and transgender women sex workers in Greater Kuala Lumpur, Malaysia. International Journal of STD and AIDS. 2021;32:609–19. doi:10.1177/0956462420970417.

116. Wechsberg WM, Peasant C, Kline T, Zule WA, Ndirangu J, Browne FA, et al. HIV Prevention Among Women Who Use Substances And Report Sex Work: Risk Groups Identified Among South African Women. AIDS and Behavior. 2017;21:155–66. doi:10.1007/s10461-017-1889-0.

117. Wells C, Schwartz S, Phaswana-Mafuya N, Lambert A, Kose Z, Mcingana M, et al. Characterizing the patterns of HIV disclosure to clients among South African female sex workers in Port Elizabeth. AIDS and Behavior. 2018;22:3924–32. doi:10.1007/s10461-018-2199-x.

118. White D, Wilson KS, Masese LN, Wanje G, Jaoko W, Mandaliya K, et al. Alcohol use and associations with biological markers and self-reported indicators of unprotected sex in human immunodeficiency virus-positive female sex workers in Mombasa, Kenya. Sexually Transmitted Diseases. 2016;43:642–7. doi:10.1097/OLQ.0000000000000502.

119. Lokken E, Richardson BA, Wanje G, Wilson K, Jaoko W, Kinuthia J, McClelland RS. Brief report: Incidence and correlates of unintended pregnancy in hivpositive kenyan sex workers. JAIDS Journal of Acquired Immune Deficiency Syndromes. 2019;85:11-17. doi:10.1097/QAI.0000000000002402.

120. Wilson KS, Deya R, Masese L, Simoni JM, Stoep AV, Shafi J, et al. Prevalence and correlates of intimate partner violence in HIV-positive women engaged in transactional sex in Mombasa, Kenya. International Journal of STD and AIDS. 2016;27:1194–203. doi:10.1177/0956462415611514.

121. Wilson KS, Deya R, Yuhas K, Simoni J, Vander Stoep A, Shafi J, et al. A Prospective Cohort Study of Intimate Partner Violence and Unprotected Sex in HIV-Positive Female Sex Workers in Mombasa, Kenya. AIDS and Behavior. 2016;20:2054–64. doi:10.1007/s10461-016-1399-5.

122. Wilson KS, Wanje G, Yuhas K, Simoni JM, Masese L, Vander Stoep A, et al. A Prospective Study of Intimate Partner Violence as a Risk Factor for Detectable Plasma Viral Load in HIV-Positive Women Engaged in Transactional Sex in Mombasa, Kenya. AIDS and Behavior. 2016;20:2065–77. doi:10.1007/s10461-016-1420-z.

123. Wilson KS. The relationship between intimate partner violence, unprotected sex, and detectable plasma viral load in HIV-positive female sex workers in Kenya: ProQuest Information & Learning; 2016.

124. Williams JE, Dangerfield DT, Kral AH, Wenger LD, Bluthenthal RN. Correlates of Sexual Coercion among People Who Inject Drugs (PWID) in Los Angeles and San Francisco, CA. Journal of Urban Health. 2019;96:469–76. doi:10.1007/s11524-018-0238-6.

125. Wirtz AL, Schwartz S, Ketende S, Anato S, Nadedjo FD, Ouedraogo HG, et al. Sexual violence, condom negotiation, and condom use in the context of sex work: Results from two West African countries. Journal of Acquired Immune Deficiency Syndromes. 2015;68:S171-S179. doi:10.1097/QAI.0000000000000451.

126. Grosso AL, Ketende S, Stahlman S, Ky-Zerbo O, Ouedraogo HG, Kouanda S, et al. Development and reliability of metrics to characterize types and sources of stigma among men who have sex with men and female sex workers in Togo and Burkina Faso. BMC Infectious Diseases 2019. doi:10.1186/s12879-019-3693-0.

127. Xie Y, Xiong M, Qi Z, Shen J, Xu X, Luo X, et al. Client-Perpetrated Violence Experience Among Female Sex Worker in Guangdong, South China: Results from a Cross-Sectional Study. AIDS and Behavior. 2023;27:806–15. doi:10.1007/s10461-022-03813-3.

128. Yeo EJ, Hlongwane K, Otwombe K, Hopkins KL, Variava E, Martinson N, et al. Key risk factors for substance use among female sex workers in Soweto and Klerksdorp, South Africa: A cross-sectional study. PLoS One 2022. doi:10.1371/journal.pone.0261855.

129. Coetzee J, Gray GE, Jewkes R. Prevalence and patterns of victimization and polyvictimization among female sex workers in Soweto, a South African township: a cross-sectional, respondent-driven sampling study. Glob Health Action 2017. doi:10.1080/16549716.2017.1403815.

130. Bird Y, Lemstra M, Rogers M, Moraros J. Third-world realities in a first-world setting: A study of the HIV/AIDS-related conditions and risk behaviors of sex trade workers in Saskatoon, Saskatchewan, Canada. Sahara J. 2016;13:152–61. doi:10.1080/17290376.2016.1229213.

131. Goldenberg SM, Buglioni N, Krusi A, Frost E, Moreheart S, Braschel M, Shannon K. Housing instability and evictions linked to elevated intimate partner and workplace violence among women sex workers in Vancouver, Canada: Findings of a prospective, community-based cohort, 2010-2019. Am J Public Health. 2023;113:442–52. doi:10.2105/AJPH.2022.307207.

132. Argento E, Muldoon KA, Duff P, Simo A, Deering KN, Shannon K. High prevalence and partner correlates of physical and sexual violence by intimate partners among street and off-street sex workers. PLoS One 2014. doi:10.1371/journal.pone.0102129.

133. Argento E, Shannon K, Nguyen P, Dobrer S, Chettiar J, Deering KN. The role of dyad-level factors in shaping sexual and drug-related HIV/STI risks among sex workers with intimate partners. Drug and Alcohol Dependence. 2015;157:166–73. doi:10.1016/j.drugalcdep.2015.10.022.

134. Argento E, Strathdee SA, Goldenberg S, Braschel M, Montaner J, Shannon K. Violence, trauma and living with HIV: Longitudinal predictors of initiating crystal methamphetamine injection among sex workers. Drug and Alcohol Dependence. 2017;175:198–204. doi:10.1016/j.drugalcdep.2017.02.014.

135. Argento E, Strathdee SA, Shoveller JA, Braschel M, Shannon K. Correlates of Suicidality Among A Community-Based Cohort of Women Sex Workers: The Protective Effect of Social Cohesion. J Interpers Violence. 2021;36:9709–24. doi:10.1177/0886260519870167.

136. Argento E, Goldenberg S, Braschel M, Machat S, Strathdee SA, Shannon K. The impact of end-demand legislation on sex workers’ access to health and sex worker-led services: A community-based prospective cohort study in Canada. PLoS One 2020. doi:10.1371/journal.pone.0225783.

137. Deering KN, Lyons T, Feng CX, Nosyk B, Strathdee SA, Montaner J, Shannon K. Client demands for unsafe sex: The socioeconomic risk environment for HIV among street and off-street sex workers. J Acquir Immune Defic Syndr. 2013;63:522–31. doi:10.1097/QAI.0b013e3182968d39.

138. Duff P, Sou J, Chapman J, Dobrer S, Braschel M, Goldenberg S, Shannon K. Poor working conditions and work stress among Canadian sex workers. Occupational Medicine. 2017;67:515–21. doi:10.1093/occmed/kqx092.

139. Goldenberg SM, Chettiar J, Nguyen P, Dobrer S, Montaner J, Shannon K. Complexities of short-term mobility for sex work and migration among sex workers: Violence and sexual risks, barriers to care, and enhanced social and economic opportunities. Journal of Urban Health. 2014;91:736–51. doi:10.1007/s11524-014-9888-1.

140. Goldenberg S, Liyanage R, Braschel M, Shannon K. Structural barriers to condom access in a community-based cohort of sex workers in Vancouver, Canada: influence of policing, violence and end-demand criminalisation. BMJ Sexual and Reproductive Health. 2020;46:301–7. doi:10.1136/bmjsrh-2019-200408.

141. Goldenberg SM, Perry C, Watt S, Bingham B, Braschel M, Shannon K. Violence, policing, and systemic racism as structural barriers to substance use treatment amongst women sex workers who use drugs: Findings of a community-based cohort in Vancouver, Canada (2010–2019). Drug and Alcohol Dependence 2022. doi:10.1016/j.drugalcdep.2022.109506.

142. Lyons T, Kerr T, Duff P, Feng C, Shannon K. Youth, violence and non-injection drug use: Nexus of vulnerabilities among lesbian and bisexual sex workers. AIDS Care - Psychological and Socio-Medical Aspects of AIDS/HIV. 2014;26:1090–4. doi:10.1080/09540121.2013.869542.

143. Machat S, Lyons T, Braschel M, Shannon K, Goldenberg S. Internet solicitation linked to enhanced occupational health and safety outcomes among sex workers in Metro Vancouver, Canada 2010–2019. Occupational and Environmental Medicine. 2022;79:373–9. doi:10.1136/oemed-2021-107429.

144. McBride B, Shannon K, Pearson J, Krüsi A, Braschel M, Goldenberg SM. Seeing pre-screened, regular clients associated with lower odds of workplace sexual violence and condom refusal amidst sex work criminalization: findings of a community-based cohort of sex workers in Metro Vancouver, Canada (2010-2019). BMC Public Health 2022. doi:10.1186/s12889-022-12903-9.

145. Muldoon KA, Deering KN, Feng CX, Shoveller JA, Shannon K. Sexual relationship power and intimate partner violence among sex workers with non-commercial intimate partners in a Canadian setting. AIDS Care - Psychological and Socio-Medical Aspects of AIDS/HIV. 2015;27:512–9. doi:10.1080/09540121.2014.978732.

146. Puri N, Shannon K, Nguyen P, Goldenberg SM. Burden and correlates of mental health diagnoses among sex workers in an urban setting. BMC Womens Health 2017. doi:10.1186/s12905-017-0491-y.

147. Socías ME, Shannon K, Montaner JS, Guillemi S, Dobrer S, Nguyen P, et al. Gaps in the hepatitis C continuum of care among sex workers in Vancouver, British Columbia: Implications for voluntary hepatitis C virus testing, treatment and care. Canadian Journal of Gastroenterology and Hepatology. 2015;29:411–6. doi:10.1155/2015/381870.

148. Sou J, Goldenberg SM, Duff P, Nguyen P, Shoveller J, Shannon K. Recent im/migration to Canada linked to unmet health needs among sex workers in Vancouver, Canada: Findings of a longitudinal study. Health Care for Women International. 2017;38:492–506. doi:10.1080/07399332.2017.1296842.

149. Pearson J, Krüsi A, Shannon K, Ettinger E, Kerrigan D, Braschel M, et al. The protective association of social cohesion on sex workers’ experiences of violence and access to community support: Impacts of resource sharing, trust and connection among a community-based cohort in Metro Vancouver, Canada (2010-2022). PLOS ONE. 2024;19:e0314749. doi:10.1371/journal.pone.0314749.

150. Argento E, Chettiar J, Nguyen P, Montaner J, Shannon K. Prevalence and correlates of nonmedical prescription opioid use among a cohort of sex workers in Vancouver, Canada. International Journal of Drug Policy. 2015;26:59–66. doi:10.1016/j.drugpo.2014.07.010.

151. Barreto D, Shoveller J, Braschel M, Duff P, Shannon K. The Effect of Violence and Intersecting Structural Inequities on High Rates of Food Insecurity among Marginalized Sex Workers in a Canadian Setting. Journal of Urban Health. 2019;96:605–15. doi:10.1007/s11524-018-0281-3.

152. Harris MT, Goldenberg S, Cui Z, Fairbairn N, Milloy M-J, Hayashi K, et al. Association of sex work and social-structural factors with non-fatal overdose among women who use drugs in Vancouver, Canada. International Journal of Drug Policy 2023. doi:10.1016/j.drugpo.2022.103950.

153. Kuosmanen J, Cabo A de. Men Selling Sex to Men in Sweden: Balancing Safety and Risk. J Interpers Violence. 2021;36:NP2601-NP2623. doi:10.1177/0886260518762448.

154. Landsberg A, Shannon K, Krüsi A, DeBeck K, Milloy M-J, Nosova E, et al. Criminalizing Sex Work Clients and Rushed Negotiations among Sex Workers Who Use Drugs in a Canadian Setting. Journal of urban health : bulletin of the New York Academy of Medicine. 2017;94:563–71. doi:10.1007/s11524-017-0155-0.

155. Logie CH, Sokolovic N, Kazemi M, Smith S, Islam S, Lee M, et al. Recent sex work and associations with psychosocial outcomes among women living with HIV: findings from a longitudinal Canadian cohort study. Journal of the International AIDS Society 2022. doi:10.1002/jia2.25874.

156. Mosnier E, Hoyer M, Artigas F, Regnault H, Richard E, Michels D, et al. Enhancing sexual health and empowerment among migrant women sex workers: a community health worker-led intervention in Marseille, France. Front Public Health. 2024;12:1359363. doi:10.3389/fpubh.2024.1359363.

157. Prangnell A, Shannon K, Nosova E, DeBeck K, Milloy M-J, Kerr T, Hayashi K. Workplace violence among female sex workers who use drugs in Vancouver, Canada: does client-targeted policing increase safety? Journal of public health policy. 2018;39:86–99. doi:10.1057/s41271-017-0098-4.

158. Richardson LA, Long C, DeBeck K, Nguyen P, Milloy M-J, Wood E, Kerr TH. Socioeconomic marginalisation in the structural production of vulnerability to violence among people who use illicit drugs. Journal of Epidemiology and Community Health. 2015;69:686–92. doi:10.1136/jech-2014-205079.

159. Swaich A, Richardson L, Cui Z, DeBeck K, Milloy M-J, Kerr T, Hayashi K. Experiences of violence during the COVID-19 pandemic among people who use drugs in a Canadian setting: a gender-based cross-sectional study. BMC Public Health 2023. doi:10.1186/s12889-023-15929-9.

160. Alemayehu M, Yohannes G, Damte A, Fantahun A, Gebrekirstos K, Tsegay R, et al. Prevalence and predictors of sexual violence among commercial sex workers in Northern Ethiopia. Reproductive Health 2015. doi:10.1186/s12978-015-0036-5.

161. Amogne MD, Balcha TT, Agardh A. Prevalence and correlates of physical violence and rape among female sex workers in Ethiopia: A cross-sectional study with respondent-driven sampling from 11 major towns. BMJ Open 2019. doi:10.1136/bmjopen-2018-028247.

162. Aristegui I, Castro Avila J, Villes V, Delabre RM, Orellano G, Aguilera M, et al. Female sex workers and police violence during the Covid-19 health crisis in 2020–2021: results from the EPIC multi-country community-based research program in Argentina. Harm Reduction Journal 2022. doi:10.1186/s12954-022-00714-5.

163. Arumugam E, Aridoss S, David JK, Jaganathasamy N, Balasubramanian G, Natesan M, et al. Injecting drug use & HIV prevalence among female sex workers: Evidence from the National Integrated Biological & Behavioural Surveillance, India. Indian Journal of Medical Research. 2022;155:413–22. doi:10.4103/ijmr.IJMR_2932_20.

164. Misra G, Sahu D, Reddy US, Nair S. Correlates of HIV prevalence among female sex workers in four north and east Indian states: findings of a national bio-behavioural survey. International Journal of STD and AIDS. 2019;30:120–30. doi:10.1177/0956462418799018.

165. Avila MM, Dos Ramos Farías, María S., Fazzi L, Romero M, Reynaga E, Marone R, Pando MA. High Frequency of Illegal Drug Use Influences Condom Use Among Female Transgender Sex Workers in Argentina: Impact on HIV and Syphilis Infections. AIDS and Behavior. 2017;21:2059–68. doi:10.1007/s10461-017-1766-x.

166. Ballester-Arnal R, Gil-Llario MD, Castro-Calvo J, Bergero-Miguel T, Guzmán-Parra J. Transgender sex work in Spain: Psychosocial profile and mental health. In: Nuttbrock LA, editor. Transgender Sex Work and Society. La Vergne: Harrington Park Press LLC; 2018. p. 350–366. doi:10.17312/harringtonparkpress/2017.11.tsws.020.

167. Beksinska A, Prakash R, Isac S, Mohan HL, Platt L, Blanchard J, et al. Violence experience by perpetrator and associations with HIV/STI risk and infection: a cross-sectional study among female sex workers in Karnataka, south India. BMJ Open 2018. doi:10.1136/BMJOPEN-2017-021389.

168. Biello KB, Thomas BE, Johnson BE, Closson EF, Navakodi P, Dhanalakshmi A, et al. Transactional sex and the challenges to safer sexual behaviors: a study among male sex workers in Chennai, India. AIDS care. 2017;29:231–8. doi:10.1080/09540121.2016.1204421.

169. Boothe M, Comé C, Semá Baltazar C, Chicuecue N, Seleme J, Chitsondzo Langa D, et al. High burden of self-reported sexually transmitted infections among key populations in Mozambique: The urgent need for an integrated surveillance system. BMC Infectious Diseases 2020. doi:10.1186/s12879-020-05276-0.

170. Bossard C, Chihana M, Nicholas S, Mauambeta D, Weinstein D, Conan N, et al. HIV, sexual violence, and termination of pregnancy among adolescent and adult female sex workers in Malawi: A respondent-driven sampling study. PLoS One 2022. doi:10.1371/journal.pone.0279692.

171. Bradley J, Rajaram S, Moses S, Gowda GC, Pushpalatha R, Ramesh BM, et al. Female sex worker client behaviors lead to condom breakage: a prospective telephone-based survey in Bangalore, South India. AIDS Behav. 2013;17:559–67. doi:10.1007/s10461-012-0192-3.

172. Bugssa G, Dessalegn B, Dimtsu B, Berhane Y. Prevalence and factors associated with HIV and hepatitis B virus infections among female commercial sex workers in mekelle, Ethiopia: Cross sectional study. International Journal of Pharmaceutical Sciences and Research. 2015;6:135–46. doi:10.13040/IJPSR.0975-8232.6(1).135-46.

173. Chabata ST, Hensen B, Chiyaka T, Mushati P, Busza J, Floyd S, et al. Condom use among young women who sell sex in Zimbabwe: a prevention cascade analysis to identify gaps in HIV prevention programming. Journal of the International AIDS Society 2020. doi:10.1002/jia2.25512.

174. Chabata ST, Hensen B, Chiyaka T, Mushati P, Musemburi S, Dirawo J, et al. The impact of the DREAMS partnership on HIV incidence among young women who sell sex in two Zimbabwean cities: Results of a non-randomised study. BMJ Global Health 2021. doi:10.1136/bmjgh-2020-003892.

175. Crowell TA, Keshinro B, Baral SD, Schwartz SR, Stahlman S, Nowak RG, et al. Stigma, access to healthcare, and HIV risks among men who sell sex to men in Nigeria. Journal of the International AIDS Society 2017. doi:10.7448/IAS.20.1.21489.

176. Daka D, Hailemeskel G, Fenta DA. Prevalence of Hepatitis B Virus infection and associated factors among female sex workers using respondent-driven sampling in Hawassa City, Southern Ethiopia. BMC Microbiology 2022. doi:10.1186/s12866-022-02444-x.

177. Daka D, Hailemeskel G, Fenta DA. Seroprevalence of hepatitis b virus and associated factors among female sex workers using respondent-driven sampling in hawassa city, ethiopia. Infection and Drug Resistance. 2021;14:4301–11. doi:10.2147/IDR.S332333.

178. Davis A, Jiwatram-Negrón T, Primbetova S, Terlikbayeva A, Bilokon Y, Chubukova L, El-Bassel N. Multi-level risk factors associated with sex trading among women living with HIV in Kazakhstan: A neglected key population. International journal of STD & AIDS. 2017;28:1397–404. doi:10.1177/0956462417708678.

179. El-Bassel N, Norcini Pala A, Mukherjee TI, McCrimmon T, Mergenova G, Terlikbayeva A, et al. Association of Violence against Female Sex Workers Who Use Drugs with Nonfatal Drug Overdose in Kazakhstan. JAMA Network Open 2020. doi:10.1001/jamanetworkopen.2020.20802.

180. Faini D, Msafiri F, Munseri P, Bakari M, Lyamuya E, Sandström E, et al. The prevalence, incidence, and risk factors for HIV among female sex workers - A cohort being prepared for a bhase IIb HIV vaccine trial in Dar es Salaam, Tanzania. Journal of Acquired Immune Deficiency Syndromes. 2022;91:439–48. doi:10.1097/QAI.0000000000003097.

181. Grosso AL, Busch S, Mothopeng T, Sweitzer S, Nkonyana J, Mpooa N, et al. HIV risks and needs related to the Sustainable Development Goals among female sex workers who were commercially sexually exploited as children in Lesotho. Journal of the International AIDS Society 2018. doi:10.1002/jia2.25042.

182. Hendrickson ZM, Leddy AM, Galai N, Mbwambo JK, Likindikoki S, Kerrigan D. Work-related mobility and experiences of gender-based violence among female sex workers in Iringa, Tanzania: A crosssectional analysis of baseline data from Project Shikamana. BMJ Open 2018. doi:10.1136/bmjopen-2018-022621.

183. Hendrickson ZM, Leddy AM, Galai N, Wilson Beckham S, Davis W, Mbwambo JK, et al. Mobility for sex work and recent experiences of gender-based violence among female sex workers in Iringa, Tanzania: A longitudinal analysis. PLoS One 2021. doi:10.1371/journal.pone.0252728.

184. Hentges B, Martins RS, Da Silva JRP, Hübner DPG, Leal AF, Teixeira LB, et al. Lifetime sexual violence among transgender women and travestis (TGW) in Brazil: Prevalence and associated factors. Revista brasileira de epidemiologia [Brazilian journal of epidemiology]. 2024;27Suppl 1:e240013.supl.1. doi:10.1590/1980-549720240013.supl.1.

185. Javalkar P, Platt L, Prakash R, Beattie T, Bhattacharjee P, Thalinja R, et al. What determines violence among female sex workers in an intimate partner relationship? Findings from North Karnataka, south India. BMC Public Health 2019. doi:10.1186/s12889-019-6673-9.

186. Katz KR, McDowell M, Green M, Jahan S, Johnson L, Chen M. Understanding the Broader Sexual and Reproductive Health Needs of Female Sex Workers in Dhaka, Bangladesh. Int Perspect Sex Reprod Health. 2015;41:182–90. doi:10.1363/4118215.

187. Lima F, Merchán-Hamann E, Urdaneta M, Damacena GN, Szwarcwald CL. Factors associated with violence against female sex workers in ten Brazilian cities. Cadernos de Saude Publica 2017. doi:10.1590/0102-311x00157815.

188. Lima A de, Magno L, Luppi CG, Szwarcwald CL, Grangeiro A, Santana EP, et al. Sexual violence and low rates of HIV post-exposure prophylaxis access among female sex workers in Brazil. AIDS and Behavior. 2022;26:4082–92. doi:10.1007/s10461-022-03734-1.

189. Longo J, Woromogo SH, Diemer H-C, Tekpa G, Nambei WS, Gresenguet G. Young women who sell sex in Bangui, Central African Republic: A neglected group highly vulnerable to HIV. Journal of Public Health (United Kingdom). 2023;45:e630-e638. doi:10.1093/pubmed/fdad130.

190. Luiz N, Muleia R, Abecasis A, Banze A, Langa D, Semá Baltazar C. Unveiling triple vulnerability among Mozambican female sex workers-Stigma, physical violence and sexual violence. PLOS ONE. 2025;20:e0312550. doi:10.1371/journal.pone.0312550.

191. Nuvunga S, Banze ÁR, Muleia R, Langa DC, Sacarlal J, Baltazar CS. Temporal trends in HIV prevention service access and use among female sex workers (FSW) in Mozambique: a comparative analysis 2011-2019. BMC Public Health. 2025;25:1995. doi:10.1186/s12889-025-23114-3.

192. Semá Baltazar C, Muleia R, Ribeiro Banze A, Boothe M. Prevalence and correlates of hazardous alcohol drinking and drug use among female sex workers and men who have sex with men in Mozambique. BMC Public Health. 2024;24:872. doi:10.1186/s12889-024-18273-8.

193. Lyons C, Grosso A, Drame FM, Ketende S, Diouf D, Ba I, et al. Physical and sexual violence affecting female sex workers in Abidjan, côte d’ivoire: Prevalence, and the relationship with the work environment, HIV, and access to health services. Journal of Acquired Immune Deficiency Syndromes. 2017;75:9–17. doi:10.1097/QAI.0000000000001310.

194. Maheu-Giroux M, Baral S, Vesga JF, Diouf D, Diabaté S, Alary M, et al. Anal intercourse among female sex workers in Côte d’Ivoire: Prevalence, determinants, and model-based estimates of the population-level impact on HIV transmission. American Journal of Epidemiology. 2018;187:287–97. doi:10.1093/aje/kwx244.

195. MacLin BJ, Wang Y, Rodriguez-Diaz C, Donastorg Y, Perez M, Gomez H, et al. Comparing typologies of violence exposure and associations with syndemic health outcomes among cisgender and transgender female sex workers living with HIV in the Dominican Republic. PLoS One 2023. doi:10.1371/journal.pone.0291314.

196. MacLin BJ, Wang Y, Rodriguez-Diaz C, Donastorg Y, Perez M, Gomez H, et al. Beyond a deficit-based approach: Characterizing typologies of assets for cisgender and transgender female sex workers and their relationship with syndemic health outcomes. PLOS Glob Public Health. 2023;3:e0002314. doi:10.1371/journal.pgph.0002314.

197. Mendoza C, Barrington C, Donastorg Y, Perez M, Fleming PJ, Decker MR, Kerrigan D. Violence from a sexual partner is significantly associated with poor HIV care and treatment outcomes among female sex workers in the Dominican Republic. Journal of Acquired Immune Deficiency Syndromes. 2017;74:273–8. doi:10.1097/QAI.0000000000001250.

198. Marquez NG, Elmi N, Lyons C, Turpin G, Moran H, Ba I, et al. Sexual violence affecting female sex workers in Côte d’Ivoire: prevalence, context, and associated mental health and substance use outcomes. BMC Public Health. 2024;24:2947. doi:10.1186/s12889-024-20177-6.

199. Milner AN, Hearld KR, Abreau N, Budhwani H, Mayra Rodriguez-Lauzurique R, Paulino-Ramirez R. Sex work, social support, and stigma: Experiences of transgender women in the Dominican Republic. International Journal of Transgenderism. 2019;20:403–12. doi:10.1080/15532739.2019.1596862.

200. Mizinduko MM, Moen K, Likindikoki S, Mwijage A, Leyna GH, Makyao N, et al. HIV prevalence and associated risk factors among female sex workers in Dar es Salaam, Tanzania: tracking the epidemic. International Journal of STD and AIDS. 2020;31:950–7. doi:10.1177/0956462420917848.

201. Ngale K, Cummings B, Horth R. Unseen, unheard and unprotected: prevalence and correlates of violence among female sex workers in Mozambique. Culture, Health and Sexuality. 2019;21:898–913. doi:10.1080/13691058.2018.1524512.

202. Ochonye BB, Sanni OF, Abiodun PO, Tewobola O, Ogbonna N. Gender-Based Violence Against Female Sex Workers in Nigeria How Helpful Are Grassroots Interventions? International Journal of Occupational Safety and Health. 2023;13:313–20. doi:10.3126/ijosh.v13i3.45980.

203. Onoja AJ, Sanni F, Shaibu J, Onoja S, Oguche D, Adamu I, Abiodun PO. Baseline and postintervention assessment of sexual violence and condom use among female sex workers in a semiurban African community. Social Health and Behavior. 2020;3:124–9. doi:10.4103/SHB.SHB_29_20.

204. Pando MA, Coloccini RS, Reynaga E, Rodriguez Fermepin M, Gallo Vaulet L, Kochel TJ, et al. Violence as a barrier for HIV prevention among female sex workers in Argentina. PLoS One 2013. doi:10.1371/journal.pone.0054147.

205. Patel SK, Saggurti N, Pachauri S, Prabhakar P. Correlates of Mental Depression Among Female Sex Workers in Southern India. Asia-Pacific Journal of Public Health. 2015;27:809–19. doi:10.1177/1010539515601480.

206. Patel SK, Ganju D, Prabhakar P, Adhikary R. Relationship between mobility, violence and major depression among female sex workers: a cross-sectional study in southern India. BMJ Open 2016. doi:10.1136/bmjopen-2016-011439.

207. Platt L, Bowen R, Grenfell P, Stuart R, Sarker MD, Hill K, et al. The effect of systemic racism and homophobia on police enforcement and sexual and emotional violence among sex workers in East London: Findings from a cohort study. Journal of Urban Health. 2022;99:1127–40. doi:10.1007/s11524-022-00673-z.

208. Elmes J, Stuart R, Grenfell P, Walker J, Hill K, Hernandez P, et al. Effect of police enforcement and extreme social inequalities on violence and mental health among women who sell sex: findings from a cohort study in London, UK. Sexually Transmitted Infections. 2022;98:323–31. doi:10.1136/sextrans-2021-055088.

209. Rameto MA, Abdella S, Ayalew J, Tessema M, Bulti J, Bati F, Lulseged S. Prevalence and factors associated with inconsistent condom use among female sex workers in Ethiopia: findings from the national biobehavioral survey, 2020. BMC Public Health 2023. doi:10.1186/s12889-023-17253-8.

210. Debel L, Ayalew J, Abdella S, Bulti J, Bejiga B, Wariso FB, et al. Gender-based violence and associated factors among female sex workers in Ethiopia. Evidence from The National Bio-behavioral Survey, 2020. Front Public Health. 2023;11:1213725. doi:10.3389/fpubh.2023.1213725.

211. Yimam JA, Luslseged S, Tura JB, Bedassa BB, Wariso FB, Rameto MA, Abdella S. Determinants of depressive and alcohol use disorders among female sex workers in Ethiopia: evidence from a national bio-behavioral survey, 2020. BMC Psychiatry. 2024;24:344. doi:10.1186/s12888-024-05799-9.

212. Sherwood JA, Grosso A, Decker MR, Peitzmeier S, Papworth E, Diouf D, et al. Sexual violence against female sex workers in the Gambia: A cross-sectional examination of the associations between victimization and reproductive, sexual and mental health. BMC Public Health 2015. doi:10.1186/s12889-015-1583-y.

213. Silverman JG, Saggurti N, Cheng DM, Decker MR, Coleman SM, Bridden C, et al. Associations of sex trafficking history with recent sexual risk among HIV-infected FSWs in India. AIDS Behav. 2014;18:555–61. doi:10.1007/s10461-013-0564-3.

214. Srivastava A, Davis JP, Patel P, Daniel EE, Karkal S, Rice E. Polyvictimization, Sex Work, and Depressive Symptoms Among Transgender Women and Men Who Have Sex With Men. J Interpers Violence. 2022;37:NP11089-NP11109. doi:10.1177/0886260521990840.

215. Stephano EE. Prevalence of sexual assault and use of emergency contraceptives among female commercial sex workers in Dodoma city, Central Tanzania: A cross-sectional study. Tanzania Journal of Health Research. 2022;23:53–4. doi:10.4314/thrb.v23i1.1S.

216. Surti SB, Kosambiya JK, Khokhar N, Patni MM, Gohil AH, Kamdar ZN, et al. Rapid response to syphilis outbreak among female sex workers. Indian Journal of Community Medicine. 2017;42:214–7. doi:10.4103/ijcm.IJCM_254_16.

217. Szwarcwald CL, Damacena GN, Souza-Junior P de, Guimarães M, Almeida W de, Souza Ferreira AP de, et al. Factors associated with HIV infection among female sex workers in Brazil. Medicine (United States). 2018;97:S54-S61. doi:10.1097/MD.0000000000009013.

218. Tounkara FK, Diabaté S, Guédou FA, Ahoussinou C, Kintin F, Zannou DM, et al. Violence, condom breakage, and HIV infection among female sex workers in Benin, West Africa. Sexually Transmitted Diseases. 2014;41:312–8. doi:10.1097/OLQ.0000000000000114.

219. Twizelimana D, Muula AS. Unmet contraceptive needs among female sex workers (FSWs) in semi urban Blantyre, Malawi. Reproductive Health 2021. doi:10.1186/s12978-020-01064-w.

220. Urada LA, Raj A, Cheng DM, Quinn E, Bridden C, Blokhina EA, et al. History of intimate partner violence is associated with sex work but not sexually transmitted infection among HIV-positive female drinkers in Russia. International Journal of STD and AIDS. 2013;24:287–92. doi:10.1177/0956462412472809.

221. Vélez-Grau C, El-Bassel N, McCrimmon T, Chang M, Terlikbayeva A, Primbetova S, et al. Suicidal ideation among women who engage in sex work and have a history of drug use in Kazakhstan. Mental Health and Prevention 2021. doi:10.1016/j.mhp.2021.200208.

222. Wirtz AL, Peryshkina A, Mogilniy V, Beyrer C, Decker MR. Current and recent drug use intensifies sexual and structural HIV risk outcomes among female sex workers in the Russian Federation. International Journal of Drug Policy. 2015;26:755–63. doi:10.1016/j.drugpo.2015.04.017.

223. Decker MR, Wirtz AL, Moguilnyi V, Peryshkina A, Ostrovskaya M, Nikita M, et al. Female sex workers in three cities in Russia: HIV prevalence, risk factors and experience with targeted HIV prevention. AIDS and Behavior. 2014;18:562–72. doi:10.1007/s10461-013-0577-y.

224. Peitzmeier SM, Wirtz AL, Peryshkina A, Sherman S, Colantuoni E, Beyrer C, Decker MR. Associations Between Violence and HIV Risk Behaviors Differ by Perpetrator Among Russian Sex Workers. AIDS and Behavior. 2020;24:812–22. doi:10.1007/s10461-019-02668-5.

225. Peitzmeier SM, Wirtz AL, Beyrer C, Peryshkina A, Sherman SG, Colantuoni E, Decker MR. Polyvictimization among Russian sex workers: Intimate partner, police, and pimp violence cluster with client violence. J Interpers Violence. 2021;36:NP8056-NP8081. doi:10.1177/0886260519839431.

226. Witte SS, Pala AN, Mukherjee TI, Yang LS, McCrimmon T, Mergenova G, et al. Reducing Partner Violence Against Women who Exchange Sex and use Drugs through a Combination Microfinance and HIV Risk Reduction Intervention: A Cluster Randomized Trial. AIDS and Behavior. 2023;27:4084–93. doi:10.1007/s10461-023-04122-z.

227. George PE, Bayer AM, Garcia PJ, Perez-Lu JE, Burke JG, Coates TJ, Gorbach PM. Is intimate partner and client violence associated with condomless anal intercourse and HIV among male sex workers in Lima, Peru? AIDS and Behavior. 2016;20:2078–89. doi:10.1007/s10461-016-1327-8.

228. Kloek M, Dijkstra M. Sex work, stigma and violence in the netherlands. Netherlands; 2018.

229. Latimer RL, Vodstrcil LA, Fairley CK, Cornelisse VJ, Chow E, Read T, Bradshaw CS. Non-consensual condom removal, reported by patients at a sexual health clinic in Melbourne, Australia. PLoS One 2018. doi:10.1371/journal.pone.0209779.

230. Reed E, West BS, Frost E, Salazar M, Silverman JG, McIntosh CT, et al. Economic vulnerability, violence, and sexual risk factors for HIV among female sex workers in Tijuana, Mexico. AIDS and Behavior 2022. doi:10.1007/s10461-022-03670-0.

231. Rodríguez EM, Fuentes P, Ramos-Lira L, Gutiérrez R, Ruiz E. Violencia en el entorno laboral del trabajo sexual y consumo de sustancias en mujeres mexicanas = Violence in the work environment of sex work and substance use in a group of Mexican women. Salud Mental. 2014;37:355–60. doi:10.17711/SM.0185-3325.2014.041.

232. Surís J-C, Stadelmann S, Auderset D, Barrense-Dias Y. Transactional sex among young people in Switzerland: A cross-sectional study. Sexual Health. 2022;18:445–52. doi:10.1071/SH21104.

233. International Committee on the Rights of Sex Workers in Europe (ICRSE). Undeserving victims?: A community report on migrant sex worker victims of crime in Europe; 2020.

234. European Sex Workers’ Rights Alliance (ESWA). NEW RESEARCH | Exposed from all sides: The role of policing in sex workers’ access to justice; November 11, 2024.

235. European Sex Workers’ Rights Alliance (ESWA). Police violence against sex workers in europe: Policy brief; June 02, 2025.

236. Beletsky L, Lozada R, Gaines T, Abramovitz D, Staines H, Vera A, et al. Syringe confiscation as an HIV risk factor: The public health implications of arbitrary policing in Tijuana and Ciudad Juarez, Mexico. Journal of Urban Health. 2013;90:284–98. doi:10.1007/s11524-012-9741-3.

237. Beletsky L, Martinez G, Gaines T, Nguyen L, Lozada R, Rangel G, et al. Mexico’s northern border conflict: Collateral damage to health and human rights of vulnerable groups. Revista Panamericana de Salud Publica/Pan American Journal of Public Health. 2012;31:403–10. doi:10.1590/S1020-49892012000500008.

238. McDougal L, Strathdee SA, Rangel G, Martinez G, Vera A, Sirotin N, et al. Adverse pregnancy outcomes and sexual violence among female sex workers who inject drugs on the United States-Mexico border. Violence and Victims. 2013;28:496–512. doi:10.1891/0886-6708.11-00129.

239. Stockman JK, Morris MD, Martinez G, Lozada R, Patterson TL, Ulibarri MD, et al. Prevalence and correlates of female condom use and interest among injection drug-using female sex workers in two Mexico-US border cities. AIDS and Behavior. 2012;16:1877–86. doi:10.1007/s10461-012-0235-9.

240. Strathdee SA, Lozada R, Martinez G, Vera A, Rusch M, Nguyen L, et al. Social and structural factors associated with HIV infection among female sex workers who inject drugs in the Mexico-US border region. PLoS One. 2011;6:e19048. doi:10.1371/journal.pone.0019048.

241. West BS, Abramovitz D, Staines H, Vera A, Patterson TL, Strathdee SA. Predictors of Injection Cessation and Relapse among Female Sex Workers who Inject Drugs in Two Mexican-US Border Cities. Journal of Urban Health. 2016;93:141–54. doi:10.1007/s11524-015-9995-7.

242. West BS, Becerra Ramirez M, Bristow CC, Abramovitz DA, Vera A, Staines H, et al. Correlates of trichomoniasis among female sex workers who inject drugs in two Mexico-US border cities. International Journal of STD and AIDS. 2020;31:866–75. doi:10.1177/0956462420929463.

243. West BS, Henry BF, Agah N, Vera A, Beletsky L, Rangel MG, et al. Typologies and Correlates of Police Violence Against Female Sex Workers Who Inject Drugs at the Mexico-United States Border: Limits of *De Jure* Decriminalization in Advancing Health and Human Rights. J Interpers Violence. 2022;37:NP8297-NP8324. doi:10.1177/0886260520975820.

244. Ulibarri MD, Hiller SP, Lozada R, Rangel MG, Stockman JK, Silverman JG, v.d. Ojeda. Prevalence and characteristics of abuse experiences and depression symptoms among injection drug-using female sex workers in mexico. Journal of Environmental and Public Health 2013. doi:10.1155/2013/631479.

245. Cepeda A, Nowotny KM. A border context of violence: Mexican female sex workers on the U.S.-Mexico border. Violence Against Women. 2014;20:1506–31. doi:10.1177/1077801214557955.

246. Geller RJ, Decker MR, Adedimeji AA, Weber KM, Kassaye S, Taylor TN, et al. A prospective study of exposure to gender-based violence and risk of sexually transmitted infection acquisition in the women’s interagency hiv study, 1995-2018. Journal of Women’s Health. 2020;29:1256–67. doi:10.1089/jwh.2019.7972.

247. Goldenberg SM, Rangel G, Staines H, Vera A, Lozada R, Nguyen L, et al. Individual, interpersonal, and social-structural correlates of involuntary sex exchange among female sex workers in two Mexico-U.S. border cities. Journal of Acquired Immune Deficiency Syndromes. 2013;63:639–46. doi:10.1097/QAI.0b013e318296de71.

248. Jain JP, Strathdee SA, Patterson TL, Semple SJ, Harvey-Vera A, Magis-Rodríguez C, et al. Perceived barriers to pre-exposure prophylaxis use and the role of syndemic factors among female sex workers in the Mexico-United States border region: a latent class analysis. AIDS care. 2020;32:557–66. doi:10.1080/09540121.2019.1626338.

249. Lafort Y, Greener R, Roy A, Greener L, Ombidi W, Lessitala F, et al. Sexual and reproductive health services utilization by female sex workers is context-specific: Results from a cross-sectional survey in India, Kenya, Mozambique and South Africa. Reproductive Health. 2017;14:1–10. doi:10.1186/s12978-017-0277-6.

250. Lafort Y, Greener L, Lessitala F, Chabeda S, Greener R, Beksinska M, et al. Effect of a ‘diagonal’ intervention on uptake of HIV and reproductive health services by female sex workers in three sub-Saharan African cities. Tropical Medicine and International Health. 2018;23:774–84. doi:10.1111/tmi.13072.

251. Lyons C, Schwartz SR, Murray SM, Shannon K, Diouf D, Mothopeng T, et al. The role of sex work laws and stigmas in increasing HIV risks among sex workers. Nature Communications 2020. doi:10.1038/s41467-020-14593-6.

252. Olakunde BO, Adeyinka DA, Ujam C, Cherkos AS, Yahaya HB, Ndukwe CD, Anenih JO. Sexual Violence and the Increased Risk of HIV among MSM in Nigeria. AIDS Behav. 2025;29:2117–24. doi:10.1007/s10461-025-04675-1.

253. Oldenburg CE, Perez-Brumer AG, Biello KB, Landers SJ, Rosenberger JG, Novak DS, et al. Transactional sex among men who have sex with men in Latin America: Economic, sociodemographic, and psychosocial factors. Am J Public Health. 2015;105:e95-e102. doi:10.2105/AJPH.2014.302402.

254. Semple SJ, Stockman JK, Pitpitan EV, Strathdee SA, Chavarin CV, Mendoza D, et al. Prevalence and correlates of client-perpetrated violence against female sex workers in 13 Mexican cities. PLoS One 2015. doi:10.1371/journal.pone.0143317.

255. Silverman JG, Servin A, Goldenberg SM, Magis-Rodriguez C, Ritter J, Raj A, Brouwer KC. Sexual violence and HIV infection associated with adolescent vs. adult entry into the sex trade in Mexico. JAMA - Journal of the American Medical Association. 2015;314:516–8. doi:10.1001/jama.2015.7376.

256. Zalla LC, Herce ME, Edwards JK, Michel J, Weir SS. The burden of HIV among female sex workers, men who have sex with men and transgender women in Haiti: results from the 2016 Priorities for Local AIDS Control Efforts (PLACE) study. Journal of the International AIDS Society 2019. doi:10.1002/jia2.25281.

257. Tocci B. The Mental Health Management of Individuals in Sex Work. Milwaukee, Wisconsin, USA: Marquette University; 2024.

258. Antwi AA, Ross MW, Markham C. Occupational Health and Safety among Female Commercial Sex Workers in Ghana: A Qualitative Study. SEXES. 2023;4:26–37. doi:10.3390/sexes4010003.

259. Bazzi AR, Yotebieng K, Otticha S, Rota G, Agot K, Ohaga S, Syvertsen JL. PrEP and the syndemic of substance use, violence, and HIV among female and male sex workers: a qualitative study in Kisumu, Kenya. Journal of the International AIDS Society 2019. doi:10.1002/jia2.25266.

260. Beaujolais B, Kaloga M, Karandikar S, Gezinski LB, Kadambari P, Maskey K. Client-Perpetrated Violence Toward Female Sex Workers in Kathmandu. Violence Against Women. 2020;26:249–67. doi:10.1177/1077801219832117.

261. Cange CW, LeBreton M, Saylors K, Billong S, Tamoufe U, Fokam P, Baral S. Female sex workers’ empowerment strategies amid HIV-related socioeconomic vulnerabilities in Cameroon. Culture, Health and Sexuality. 2017;19:1053–65. doi:10.1080/13691058.2017.1291993.

262. Dewey S, St. Germain T. “It Depends on the Cop:” Street-Based Sex Workers’ Perspectives on Police Patrol Officers. Sexuality research & social policy : journal of NSRC : SR & SP. 2014;11:256–70. doi:10.1007/s13178-014-0163-8.

263. Katumba KR, Haumba M, Mayanja Y, Machira YW, Gafos M, Quaife M, et al. Understanding the contexts in which female sex workers sell sex in Kampala, Uganda: a qualitative study. BMC Womens Health. 2024;24:371. doi:10.1186/s12905-024-03216-7.

264. Khofi L, Manderson L, Moyer E. Food insecurity, intimate partner violence, and barriers to sexual and reproductive health care among women in Lorentzville, South Africa. Soc Sci Med. 2025;369:117785. doi:10.1016/j.socscimed.2025.117785.

265. Kyriakakis S, Compton-Almo C, Goddard-Durant S. Subsistence and Survival: Strategies Women in the Republic of Barbados Engaged in Transactional Sex Work Employ to Stay Safe. Journal of Aggression, Maltreatment & Trauma. 2024;33:1280–98. doi:10.1080/10926771.2024.2332604.

266. Levine EC. Female-to-male to mistress: A layered account of layered performances. Sexualities. 2021;24:252–75. doi:10.1177/1363460720931329.

267. Lim S, Peitzmeier S, Cange C, Papworth E, LeBreton M, Tamoufe U, et al. Violence against female sex workers in Cameroon: accounts of violence, harm reduction, and potential solutions. J Acquir Immune Defic Syndr. 2015;68 Suppl 2:S241-7. doi:10.1097/QAI.0000000000000440.

268. Maher L, Dixon T, Phlong P, Mooney-Somers J, Stein E, Page K. Conflicting rights: How the prohibition of human trafficking and sexual exploitation infringes the right to health of female sex workers in Phnom Penh, Cambodia. Health Hum Rights. 2015;17:E102-13.

269. Marlow HM, Shellenberg K, Yegon E. Abortion services for sex workers in Uganda: Successful strategies in an urban clinic. Culture, Health and Sexuality. 2014;16:931–43. doi:10.1080/13691058.2014.922218.

270. Mbonye M, Rutakumwa R, Weiss H, Seeley J. Alcohol consumption and high risk sexual behaviour among female sex workers in Uganda. African Journal of AIDS Research. 2014:145–51. doi:10.2989/16085906.2014.927779.

271. Nattabi J, Sensoy Bahar O, Nabayinda J, Nabunya P, Kiyingi J, Kizito S, et al. Crossroads of choice: a qualitative study of the factors influencing decisions to transition from sex work among women engaged in sex work in Southern Uganda. BMC Womens Health. 2025;25:196. doi:10.1186/s12905-025-03631-4.

272. Okanlawon K, Adebowale AS, Titilayo A. Sexual hazards, life experiences and social circumstances among male sex workers in Nigeria. Culture, health & sexuality. 2013;15 Suppl:22–33. doi:10.1080/13691058.2012.754053.

273. Onyango MA, Adu-Sarkodie Y, Agyarko-Poku T, Asafo MK, Sylvester J, Wondergem P, et al. “It’s all about making a life”: Poverty, HIV, violence, and other vulnerabilities faced by young female sex workers in Kumasi, Ghana. Journal of Acquired Immune Deficiency Syndromes. 2015;68:S131-S137. doi:10.1097/QAI.0000000000000455.

274. Panneh M, Gafos M, Nyariki E, Liku J, Shah P, Wanjiru R, et al. Mental health challenges and perceived risks among female sex Workers in Nairobi, Kenya. BMC Public Health. 2022;22:2158. doi:10.1186/s12889-022-14527-5.

275. Wanjiru R, Nyariki E, Babu H, Lwingi I, Liku J, Jama Z, et al. Beaten but not down! Exploring resilience among female sex workers (FSWs) in Nairobi, Kenya. BMC Public Health 2022. doi:10.1186/s12889-022-13387-3.

276. Preble K, Magruder K, Cimino AN. It’s like being an electrician, you’re gonna get shocked’: Differences in the perceived risks of indoor and outdoor sex work and its impact on exiting. 2021;14:625–46. doi:10.1080/15564886.2019.1630043.

277. Shepp V. Seeking support under the state: Aex worker’s experiences navigating gender-based violence services: ProQuest Information & Learning; 2023.

278. Sherman SG, Footer K, Illangasekare S, Clark E, Pearson E, Decker MR. “What makes you think you have special privileges because you are a police officer?” A qualitative exploration of police’s role in the risk environment of female sex workers. AIDS Care - Psychological and Socio-Medical Aspects of AIDS/HIV. 2015;27:473–80. doi:10.1080/09540121.2014.970504.

279. Siegel K, Cabán M, Brown-Bradley CJ, Schrimshaw EW. Experiences of interpersonal violence among a diverse sample of male sex workers. Culture, Health and Sexuality. 2024;26:531–45. doi:10.1080/13691058.2023.2231049.

280. Siegel K, Cabán M, Brown-Bradley CJ, Schrimshaw EW. Male Sex Workers’ Strategies to Manage Client-Related Risks of Violence. J Interpers Violence. 2023;38:10814–38. doi:10.1177/08862605231176804.

281. Simmons B, Syvertsen JL. Learning from women who trade sex in Kenya about the antiblackness of Global Health. Social Science and Medicine 2022. doi:10.1016/j.socscimed.2022.115246.

282. Benner BE. HIV vulnerability among survival sex workers through sexual violence and drug taking in a qualitative study from Victoria, Canada, with additional implications for pre-exposure prophylaxis for sex workers. Frontiers in Sociology 2022. doi:10.3389/fsoc.2021.714208.

283. Bungay V, Guta A. Strategies and Challenges in Preventing Violence Against Canadian Indoor Sex Workers. Am J Public Health. 2018;108:393–8. doi:10.2105/AJPH.2017.304241.

284. Dawthorne N. Intelligible variability: Narratives of male sex work in London Ontario Canada: ProQuest Information & Learning; 2023.

285. Krüsi A, Kerr T, Taylor C, Rhodes T, Shannon K. ‘They won’t change it back in their heads that we’re trash’: the intersection of sex work-related stigma and evolving policing strategies. Sociol Health Illn. 2016;38:1137–50. doi:10.1111/1467-9566.12436.

286. Krüsi A, Pacey K, Bird L, Taylor C, Chettiar J, Allan S, et al. Criminalisation of clients: Reproducing vulnerabilities for violence and poor health among street-based sex workers in Canada - A qualitative study. BMJ Open 2014. doi:10.1136/bmjopen-2014-005191.

287. Lyons T, Krüsi A, Pierre L, Kerr T, Small W, Shannon K. Negotiating violence in the context of transphobia and criminalization: The experiences of trans sex workers in Vancouver, Canada. Qualitative health research. 2017;27:182–90. doi:10.1177/1049732315613311.

288. Yaakobovitch T, Bensimon M, Idisis Y. A qualitative analysis of male actors in amateur pornography: motivations, implications and challenges. Curr Psychol. 2024;43:27084–202. doi:10.1007/s12144-024-06329-2.

289. Aborisade RA. Police abuse of sex workers in Nigeria: evidence from a qualitative study. Police Practice and Research. 2019;20:405–19. doi:10.1080/15614263.2018.1500283.

290. Eshetu HB, Zewdie A, Girma E, Kassie A, Adugna A, Nigusie A, Handebo S. “Many People Do Not Consider Us Human” Violence Against Commercial Sex Workers in Gondar City: A Phenomenological Study. SAGE Open 2025. doi:10.1177/21582440251324724.

291. Friend J. Digital privacy is a sexual health necessity: a community-engaged qualitative study of virtual sex work and digital autonomy in Senegal. Sexual and Reproductive Health Matters 2023. doi:10.1080/26410397.2023.2272741.

292. Kiernan B, Mishori R, Masoda M. ‘There is fear but there is no other work’: a preliminary qualitative exploration of the experience of sex workers in eastern Democratic Republic of Congo. Culture, Health and Sexuality. 2016;18:237–48. doi:10.1080/13691058.2015.1073790.

293. Mashumba L. ‘Blowjobs are jobs too’: An exploratory study into victimization experiences of male sex workers in Botswana. Sexuality and Culture. 2024;28:54–70. doi:10.1007/s12119-023-10105-y.

294. Matheson C, Bon L, Bowman L, Hannah A, Macleod K. Vulnerability, risk and harm for people who use drugs and are engaged in transactional sex: Learning for service delivery. Int J Environ Res Public Health 2022. doi:10.3390/ijerph19031840.

295. Nichols AJ. Intersections of gender and sexuality in police abuses against transgender sex workers in Sri Lanka. In: Nichols AJ, editor. Handbook of LGBT Communities, Crime, and Justice; 2014. p. 165–182. doi:10.1007/978-1-4614-9188-0_9.

296. Panchanadeswaran S, Vijayakumar G, Chacko S, Subramaniam S, Dasari S, Lee S, Brazda M. “A little love is enough for me to live my life…”: Precarity and resilience among older sex workers in India. J Women Aging. 2024;36:410–26. doi:10.1080/08952841.2024.2360259.

297. Roman N. Prostitution in Havana, Cuba, a conflict analysis of ‘The phenomenon of Jineterismo in Havana, Cuba’ A narrative study: ProQuest Information & Learning; 2021.

298. Ryan P, McGarry K. ‘I miss being honest’: Sex workers’ accounts of silence and disclosure with health care providers in Ireland. Culture, Health and Sexuality. 2022;24:688–701. doi:10.1080/13691058.2021.1879271.

299. Smaniotto Gehlen RG, Da Costa MC, Arboit J, Da Silva EB. Instances of vulnerability to violence experienced by female sex workers: A case study. Ciencia y Enfermeria 2018. doi:10.4067/s0717-95532018000100208.

300. Twizelimana D, Muula AS. Hiv and aids risk perception among sex workers in semi-urban blantyre, Malawi. Tanzania Journal of Health Research 2015. doi:10.4314/thrb.v17i3.5.

301. Zarhin D, Fox N. ‘Whore stigma’ as a transformative experience: altered cognitive expectations among Jewish-Israeli street-based sex workers. Culture, Health and Sexuality. 2017;19:1078–91. doi:10.1080/13691058.2017.1292367.

302. Armstrong L. “Who’s the slut, who’s the whore?”. Feminist Criminology. 2016;11:285–303. doi:10.1177/1557085115588553.

303. Crago A-L. Failures of justice: State and non-state violence against sex workers; 2015.

304. European Sex Workers’ Rights Alliance (ESWA). “Two pairs of gloves”; 2023.

305. European Sex Workers’ Rights Alliance (ESWA). No place to call home: How housing policies and criminalisation impact migrant sex workers; May 14, 2025.

306. Meiliana S. Cultural violence: Feminist power analysis on Ronggeng performing art. Research in Dance Education 2023. doi:10.1080/14647893.2023.2258804.

307. Nelson E-U. Structural inequities, HIV vulnerability and women’s agency: Street-based sex workers in Nigeria. Global Public Health. 2020:1800–9. doi:10.1080/17441692.2020.1791211.

308. Oselin SS, Blasyak A. Contending with Violence: Female Prostitutes’ Strategic Responses on the Streets. Deviant Behavior. 2013;34:274–90. doi:10.1080/01639625.2012.735896.

309. Scorgie F, Nakato D, Harper E, Richter M, Maseko S, Nare P, et al. ‘We are despised in the hospitals’: Sex workers’ experiences of accessing health care in four African countries. Culture, Health and Sexuality. 2013;15:450–65. doi:10.1080/13691058.2012.763187.

310. Scorgie F, Vasey K, Harper E, Richter M, Nare P, Maseko S, Chersich MF. Human rights abuses and collective resilience among sex workers in four African countries: A qualitative study. Globalization and Health 2013. doi:10.1186/1744-8603-9-33.

311. Spyrelis A, Ibisomi L. “It is just a lot to deal with”: A qualitative study exploring the sexual and reproductive health needs of a sample of female sex workers in six locations in Southern Africa. African Journal of Reproductive Health. 2022;26:72–80. doi:10.29063/ajrh2022/v26i5.8.
