# Supplementary material for: Systematic review of sexual violence against sex workers: implications for mental and sexual health
Source: BMC Public Health. 2026 Jun 30;26:2126. doi: 10.1186/s12889-026-28204-4 (PMC13360242; doi:10.1186/s12889-026-28204-4)
Supplement: Supplementary file 4 — Additional file 4. Details on qualitative results. [file 12889_2026_28204_MOESM4_ESM.docx]

**Systematic review of sexual violence against sex workers: Implications for mental and sexual health**

**Additional file 4**

Marie Püffel1, İsmail Orbay2*, Ira Salo3*, Henriette Berg1*, Lea Hasanagic1*, Elisa Ruiz Burga4, Thérèse Bernier5, Nina Heinrichs1

1Bielefeld University | Department of Psychology | Bielefeld | Germany

2Protestant University of Applied Sciences Berlin | Department of Social Work | Berlin | Germany

3University of Turku | Faculty of Law | Turku | Finland

4University College London | Institute of Global Health | London | United Kingdom

5George Brown Polytechnic | Faculty of Applied Science, Construction and Engineering Technology | Toronto | Canada

* Authors had same amount of contribution to paper

**Table A Overview of qualitative results by theme**

| **Sexual violence theme** | **Subthemes including references** | **Example finding and illustration** |
| --- | --- | --- |
| **Exploitation in context of sex work** | **Varying from forced with physical violence to limited consent due to economical pressure**  (Shepp 2023; Roman 2021; Dawthorne 2023; Decker et al. 2013; Siegel et al. 2023a) | **Finding:** “Others were more explicitly recruited by pimps out side the context of an existing relationship. In speaking  to the potential for severe violence, one such participant describes her pimp’s control, enforced with the threat of a gun, and its culmination in her purposeful arrest as a  means of escaping him. Pimps exploited women’s social vulnerabilities, in the following case homelessness, as a means of establishing their control”  **Illustration:** “He wasn’t going to let me out of his sight. He had a room upstairs down the street somewhere, up over this store or something. He was always going to watch me. It was like, basically, he was kidnapping me. That’s what he was trying to say. Like, “You haven’t got anywhere to go.” She is homeless. “I got you. You aren’t going to leave me. You’re going to make that money right here in the basement, and you’re going to be with me.” …Yeah, he wanted half of it. Then he goes, “Well, you’re mine. You work for me.” I’m like, “How am I going to get away from this guy?” He had a gun and everything. He was a pimp… What I did was I went on the street, and when I saw the cops, I pulled my dress up like this, and I said, “You want a blow job,” in front of the cops so that they could arrest me. That was the only way that was going to save me from the pimp.” (p.7)  (Decker et al. 2013) |
| **Sexual assault and coercion = (attempted) unconsented penetration (anal, oral, vaginal with genital and/or object)** | **(Attempted) coerced penetration**    (Bazzi et al. 2019; Crago 2015; Decker et al. 2013; Okanlawon et al. 2013; Preble et al. 2021; Scorgie and Nakato et al. 2013; Scorgie and Vasey et al. 2013; Shepp 2023; Sherman et al. 2015; Beaujolais et al. 2020; Cange et al. 2019; Dawthorne 2023; Katz KR et al. 2015; Kloek and Dijkstra 2018; Levine 2021; Nelson 2020; Nichols 2014; Oselin and Blasyak 2013; Panneh et al. 2022; Smaniotto Gehlen et al. 2018; Spyrelis and Ibisomi 2022; Katumba et al. 2024; Eshetu et al. 2025; European Sex Workers' Rights Alliance November 11, 2024, June 02, 2025; Nattabi et al. 2025; Pokharel et al. 2024; Khofi et al. 2025)   - **using (threats of) physical violence**   (Aborisade 2019; Benner 2022; Beaujolais et al. 2020; Cepeda and Nowotny 2014; Crago 2015; Decker et al. 2013; Kiernan et al. 2016; Kloek and Dijkstra 2018; Lim S et al. 2015; Mashumba 2024; Nichols 2014; Okanlawon et al. 2013; Oselin and Blasyak 2013; Roman 2021; Shepp 2023; Siegel et al. 2023a; Smaniotto Gehlen et al. 2018; Katumba et al. 2024; Tocci 2024; Pokharel et al. 2024)   - **without using (threats of) physical violence**   (Antwi et al. 2023; Beaujolais et al. 2020; Crago 2015; Nelson 2020; Scorgie and Vasey et al. 2013; European Sex Workers' Rights Alliance May 14, 2025; Pokharel et al. 2024) | - **using (threats of) physical violence**   **Finding:** “Furthermore, non-use of condom was coupled with extreme violence, which women perceived as stemming from stigma and a specific antagonism toward sex workers that they had similarly perceived from client perpetrators. That is, police were particularly brutal in their assault, punishing women for being sex workers”  **Illustration:** “You take them like all the rest of your clients but. when a uniformed man takes you, they are always brutal. He doesn’t want to do you. in a way that should be done. He wants to assault you. as if he is saying ah here’s a woman from the street. I should destroy her this one time” (p. S243)  (Lim S et al. 2015)   - **without using (threats of) physical violence**   **Finding:** “While clients and police are perhaps the most visible role-players in the working environment of sex workers, a wide range of people, including landlords, hotel and bar staff, security guards and brothel owners, are either centrally involved in the sex trade or operate on its fringes. Our research found that these individuals frequently take advantage of sex workers’ vulnerable position and the illegality of sex work to extort money or sex.”  **Illustration:** “One day I was harassed by a client and when I told the bar manager he demanded sex so that he can help me. My landlord also demanded sex because I could not afford rent.” (25 year old female, Mombasa)” (p. 6)  (Scorgie and Vasey et al. 2013) |
|  | **Coerced penetration in exchange of:**   - **freedom / non-arrestment / “Subbotnick”**   (Crago 2015; Aborisade 2019; Decker et al. 2013; Dewey and St. Germain 2014; Lim S et al. 2015; Mbonye et al. 2014; Nichols 2014; Onyango et al. 2015; Sherman et al. 2015; Simmons and Syvertsen 2022; Spyrelis and Ibisomi 2022; Twizelimana and Muula 2015; European Sex Workers' Rights Alliance November 11, 2024, June 02, 2025; Kyriakakis et al. 2024; Eshetu et al. 2025; Khofi et al. 2025)   - **Basic human needs while incarcerated**   (European Sex Workers' Rights Alliance November 11, 2024, June 02, 2025) | - **freedom / non-arrestment / “Subbotnick”**   **Finding:** “Overwhelmingly, the primary form of police abuse was coercive sex whereby police exploited power dynamics inherent to their relationship with sex workers. The threat of arrest, largely implicit though occasionally explicit, was sufficiently powerful to coerce sex, most often oral sex, such that overt force and physical violence from police was rarely discussed. Participants often described these scenarios as exchanging sex for their freedom.”  **Illustration:** “A little bit of everything has happened to me on the street, I’ve been propositioned by police, I’ve traded my freedom and drugs from police for sex.” (p. 7)  (Decker et al. 2013)   - **Basic human needs while incarcerated**   **Finding:** “Several participants in Greece also reported being coerced into sexual acts by police officers during detention or arrest. They were told they could only use the toilet if they provided sexual services to the officer”  **Illustration:** “In a detention centre that I remember, I wanted to go to the bathroom — not just me, other girls too. They would only let us go, if we gave blowjobs to the officer through the bars. From a certain point onwards, it was a daily routine for them and for us. Some gave in, some peed in the cells on purpose” (p. 26)  (European Sex Workers' Rights Alliance June 02, 2025) |
|  | **(Attempt of) coercion of condom-less sex**  (Aborisade 2019; Bazzi et al. 2019; Dawthorne 2023; Katz KR et al. 2015; Kloek and Dijkstra 2018; Lim S et al. 2015; Mbonye et al. 2014; Onyango et al. 2015; Panneh et al. 2022; Decker et al. 2013; Crago 2015; Eshetu et al. 2025; Nattabi et al. 2025)   - **using (threats of) physical violence**   (Beaujolais et al. 2020; Cange et al. 2019; Cepeda and Nowotny 2014; Katz KR et al. 2015; Kiernan et al. 2016; Nelson 2020; Okanlawon et al. 2013; Onyango et al. 2015; Ryan and McGarry 2022; Siegel et al. 2023a; Wanjiru et al. 2022; Eshetu et al. 2025)   - **using economical pressure**   (Crago 2015; Krüsi et al. 2014; Matheson et al. 2022; Okanlawon et al. 2013; Onyango et al. 2015; Reed et al. 2022; Spyrelis and Ibisomi 2022; Twizelimana and Muula 2015) | - **using (threats of) physical violence**   **Finding:** “Participants described how clients engaged in aggressive behavior if they did not want to use condoms with the sex workers”  **Illustration**: “They used to threaten me sometimes. If I don’t agree to have sex without a condom, they try to beat me.” (p. 256)  (Beaujolais et al. 2020)   - **using economical pressure**   **Finding:** “According to the accounts provided by respondents, many clients demanded unprotected sex because they believed it would increase sexual pleasure. Sex workers reported that many clients deliberately paid less when a condom was used. Some male sex workers therefore engaged in unprotected sex in order not to lose clients who patronised them and  so as to earn better pay”  **Illustration:** “Poverty drove me into doing this and I’m unhappy. I have kissed many mouths. I’ve had sex with different people and the problem is that, some rich men that can pay well refuse condom.  They say they want to feel me ‘skin to skin’. I don’t know what they derive from it. I feel worthless and tired of this because it’s risky”  p. S26-27)  (Okanlawon et al. 2013) |
|  | **Condom sabotage / stealthing**  (Decker et al. 2013; Kuosmanen and Cabo 2021; Mashumba 2024; Onyango et al. 2015; Ryan and McGarry 2022; Smaniotto Gehlen et al. 2018; Spyrelis and Ibisomi 2022; Khofi et al. 2025; Kyriakakis et al. 2024) | **Finding:** “A major theme was also that of stealthing. Many gay and bisexual MSWs reported another phenomenon they referred to as ‘stealthing’. As described by participants, stealthing was referred to as the removal of condoms during anal sex without the sex workers’ knowledge”  **Illustration:** “…next thing I see was him coming out without a condom, I asked where the condom was thinking it was left inside of me, but he said he removed it a long time ago to enjoy me more. I got so angry, I felt like an object, like a had no right to be asked whether to remove a condom or not… (Ferocious).” (p. 60)  (Mashumba 2024)  **Finding:** “Many women reported experiences of condoms accidently breaking due to rough sex; however, they were especially concerned about intentional damage by clients. Some explained that the motive was to infect sex workers with HIV”  **Illustration:** “Some HIV victims knowingly try to infect prostitutes just because they don’t want to die alone. They may intentionally damage the condom before coming so that during sex it breaks.” (p. S135)  (Onyango et al. 2015) |
|  | **Longer sex than agreed to**  (Crago 2015; Beaujolais et al. 2020; Siegel et al. 2023a; Eshetu et al. 2025) | **Finding:** “Another factor relating to participants’ experiences  of violence was the length of time for providing services. Clients frequently insisted that sex workers continue working beyond the agreed upon length of time. (…) Rama indicated that she was forced to continue engaging in sexual activity beyond her comfort level. Attempts to establish boundaries were ignored by her clients.”  **Illustration:** “Yes, I have some problems. Some do for long periods of time, and as much as time they demand, we do it. As much time as they want, we have to give them.” […]  “It is very hard and painful . . . sometimes the client takes too much time, and they repeatedly want to have more sex than they pay for.” (p. 258)  (Beaujolais et al. 2020) |
|  | **(Attempted) gang rape, including more clients than agreed to**  (Aborisade 2019; Beaujolais et al. 2020; Cange et al. 2017; Crago 2015; Decker et al. 2013; Kuosmanen and Cabo 2021; Maher L et al. 2015; Marlow et al. 2014; Matheson et al. 2022; Nichols 2014; Oselin and Blasyak 2013; Shepp 2023; Siegel et al. 2023b; Simmons and Syvertsen 2022) | **Finding:** “The respondents expressed that the police are often insatiable in their demands for money and sex anytime they come across them while on their duty posts. In some instances, the police engage in gang rape in their bid to satisfy their sexual urge and abuse the prostitutes in the process.”  **Illustration:** “. . .we were standing at a junction around Mokola roundabout when a patrol van of the police rounded us up. We all took off but while other escaped into some corners, I made the mistake of running on the main road which made them to catch up with me easily with their vehicle. They grabbed me and threw me into the back of their van and ordered me to lie face-down on the floor of the van. They drove for some minutes and stopped at an isolated bushy area. Two of them then dragged me down from the van and ordered me to remove my cloth, which I did. They took their turns on me while I lie of the road. They were obviously drunk and were very violent, slapping, punching and pouring their drinks on me while they were at it... none of them used protection (condom), I’m not sure they even gave it a thought to use one...they were six in all. . .after they were through, they asked me to go while they drove off leaving me there at the spot. I waited till morning before I could get help” (p. 413)  (Aborisade 2019) |
|  | **Refusal of payment (including cancelation of online payment afterwards)**  (Antwi et al. 2023; Beaujolais et al. 2020; Cange et al. 2017; Cange et al. 2019; Kiernan et al. 2016; Kloek and Dijkstra 2018; Lim S et al. 2015; Mbonye et al. 2014; Okanlawon et al. 2013; Oselin and Blasyak 2013; Preble et al. 2021; Scorgie and Vasey et al. 2013; Scorgie and Nakato et al. 2013; Siegel et al. 2023a, 2023b; Smaniotto Gehlen et al. 2018; Panchanadeswaran et al. 2024; Kyriakakis et al. 2024; Eshetu et al. 2025; European Sex Workers' Rights Alliance November 11, 2024, June 02, 2025; Nattabi et al. 2025; Katumba et al. 2024) | **Finding:** “Indoor and outdoor sex workers alike spoke about the risk of loss from being robbed or cheated out of money. Only seven participants reported being robbed, but the rest “expected” it.”  **Illustration:** “I think a worst day in the sex industry is when you don’t make any money or you have a guy– or you get counterfeit money. That pisses you off … And you look in the envelope, ‘cause when he came in, he flashed the envelope … and it’s 20s and it’s dark in the room. So he hurries up, puts the money back in the envelope, and you’re like, “Okay, I saw it. It’s 20s. Whatever.” And he leaves, and you look at the envelope, and it’s only 20s on one side, because he made color copies, and you’re so– I was so fucking pissed, ‘cause he got me. He stole from me. He stole my time. He stole my energy. He was a thief.” (p. 637-638)  (Preble et al. 2021) |
| **Physically forced sexual activity (without penetration)** | **Non-consensual clothing removal**  (Crago 2015; Lim S et al. 2015; Kloek and Dijkstra 2018; Siegel et al. 2023a; European Sex Workers' Rights Alliance November 11, 2024, June 02, 2025) | **Finding:** “Client violence was reported in the context of client demands that went beyond the negotiated agreement, as illustrated by the following situation in which the original agreement was for sex without clothing removal:”  **Illustration:** “Sometimes you go to the room with the client and  he had proposed to give you 1000 francs. When  you arrive at the room and he asks you to take off  your clothing and you refuse, he’ll start to attack  you. He takes off your clothes” (p. S242-243)  (Lim S et al. 2015) |
|  | **Unwanted touching of own body (e.g., breasts, butt, genitals)**  (Dewey and St. Germain 2014; Nichols 2014; Roman 2021; Meiliana 2023; Kloek and Dijkstra 2018; European Sex Workers' Rights Alliance November 11, 2024, June 02, 2025, 2023) | **Finding:** „Another sex worker who used to work at a massage salon pointed out that clients at massage salons often try to push boundaries.” […] “The same sex worker indicated that she finds working in a massage salons far more precarious than other types of sex work.”  **Illustration:** “In many salons, a client for a tantra massage or a more expensive massage may be allowed to touch the masseuse’s breasts and buttocks, but not the vagina. My experience is that really more than half of the men will always try to get their hand between your legs and react not always kindly to a rejection.” […] “Especially because it isn’t clear up front what the service includes and what the client may expect.” (p. 20)  (Kloek and Dijkstra 2018) |
|  | **Unwanted touching of another/ perpetrators body**  (Nichols 2014; Dewey and St. Germain 2014) | **Finding:** “However, women were divided with respect to the extent that undercover officers could actually engage in sexual activities. One woman described physical contact with an officer that most other women believed prohibited”  **Illustration:** “I know in the state of Colorado, cops can pull out their stuff and have you touch it, and they can touch you. I got  hemmed up like that one time and the cop actually put  his hand down my shirt, and had me touch his stuff too,  and he was a cop. And he drove about a block and a half  from where he picked me up and there were cops  everywhere, he was a police officer, he put his hand  down my shirt, groped me, had me touch him, and he  was a cop, all day long” (p. 263)  (Dewey and St. Germain 2014) |
|  | **Voyerism**  (Nichols 2014) | **Finding:** “Some respondents reported being forced to masturbate officers, provide oral sex, and engage in sexual acts with other nachchi while the officer watched. Nalin illustrated an example of forced masturbation with another nachchi sex worker in the street by police:”  **Illustration:** “[The police] have caught [us], when we were in the park, while we were behaving [working]… police told … [another nachchi sex worker] to take my one [penis], and told me to take [the other nachchi sex worker’s] one. … After watching, told us to run and kicked us and left us…So they also must like to watch us doing it no, and we did it, so what else to do?“ (p. 176)  (Nichols 2014) |
|  | **Unwanted “rough sex” / aggressiveness right down to physical violence during intercourse**  (Siegel et al. 2023a; Cepeda and Nowotny 2014; Scorgie and Nakato et al. 2013; Scorgie and Vasey et al. 2013; Wanjiru et al. 2022; Yaakobovitch et al. 2024; Tocci 2024) | **Finding:** “Irma, a 22-year-old divorcee who worked in La Zona de Tolerancia, described a violent situation with a client whom she described as loco (crazy)”  **Illustration:** “When we arrived in the room we began to have sexual intercourse. All of a sudden he turned me around and grabbed my arms. I thought he wanted to have sex from behind but since he had not paid for that I fought back. But what he started doing was slapping my butt and pinching it. The only thing I could do was scream because it hurt. I know he was high on cocaine. I could not get loose. He would just hit me harder and all I could do was scream louder. Finally, some guards came in and kicked him out.” (p. 1518)  (Cepeda and Nowotny 2014) |
|  | **Physical violence during intercourse leading to severe sexual health conditions**  (Scorgie and Nakato et al. 2013) | **Finding:** “Some women reported frequently experiencing “lower abdominal pain” – potentially a sign of untreated STIs or other reproductive health problems. Discomfort and even injury during sexual intercourse was also common.”  **Illustration:** “I found a client, and he was bigger than me, as you can see I’m thin. He slept with me in bad way. Then when we finish, I try to stand up I feel pain inside me. And by that time I could not stand up. He took me to a hospital. The nurse told me that I have injured inside my womb then they told me that they are going to take out my womb because they can’t operate it, now I’m living without a womb.” (p. 5)  (Scorgie and Nakato et al. 2013) |
|  | **Unwanted genital examination**  (Levine 2021) | **Finding and illustration of self-ethnographic research:** “At least one prospective mistress had been forced to strip and submit to genital inspection to assess whether she qualified as a “real” woman. Although Rikki/I would have passed such an inspection, she/I might well have refused if asked and been summarily fire” (p. 259)  (Levine 2021) |
|  | **Invasive body searching by police** (European Sex Workers' Rights Alliance November 11, 2024, June 02, 2025) | **Finding:** „Several participants were also forced to remain naked during police operations at their workplaces or were subjected to invasive body searches, including strip searches. Some described being compelled by police officers to strip naked in public under the pretext of drug searches — acts that served as a form of humiliation, punishment, or expressions of arbitrary power and cruelty. Enforced and prolonged nudity is recognised as a form of inhuman and degrading treatment, a type of sexualised torture, and an act of humiliation intended to undermine personal integrity, self-esteem, and human dignity.“  **Illustration:** “I was not treated well. I was accused of something I didn’t do. They searched me in the street and stripped me naked, and they shouted at me that I was carrying drugs on me. In other words, I was treated very badly. They did the same to a colleague. I don’t think it’s right that they strip you naked in the street, take your bag, throw everything on the ground in front of everybody. “  ESWA 2024/25 (p. 24)  (European Sex Workers' Rights Alliance June 02, 2025) |
|  | **Forced Abortion**  (Decker et al. 2013; Khofi et al. 2025) | **Finding:** “The following participant illustrates the potential for both unprotected sex with pimps, as well as violence in the context of jealousy and sexual control, even when pimps themselves are arranging and profiting from sex trade.”  **Illustration:** “Then I ended up pregnant by him, and of course he beat on me severely. He would set me up with guys for money, and then after I would do it he would beat me up because he was jealous, because this guy would ask for me again or something and he would think something happened, or I was holding back on him. It was always something. I was eight months pregnant by him, and he kicked me down a flight of steps. We got arguing and he kicked me down a flight of steps, and my son was born stillborn. I got a birth certificate and a death certificate all in the same day. I think that was the hardest thing, one of the hardest things I’ve ever went through.“ (p. 6-7)  (Decker et al. 2013) |
| **In-person sexual violence without physical touch** | **Sexual verbal harassment: e.g., humiliating or ridiculing comments, insults, …**  **Often based on sex work stigma, homophobia, transphobia and/or racism**  (European Sex Workers' Rights Alliance 2023, May 14, 2025, November 11, 2024, June 02, 2025; Panneh et al. 2022; Spyrelis and Ibisomi 2022; Armstrong 2016; Meiliana 2023; Dawthorne 2023; Scorgie and Nakato et al. 2013; Scorgie and Vasey et al. 2013; Cange et al. 2019; Friend 2023; Kloek and Dijkstra 2018; Maher L et al. 2015; Nichols 2014; Nelson 2020; Tocci 2024; Panchanadeswaran et al. 2024; Yaakobovitch et al. 2024; Katumba et al. 2024; Eshetu et al. 2025) | **Finding:** „In instances, providers were reported to have made comments referring to the inevitable abandonment of sex workers by their partners, given perceived difficulties in accepting their profession. In other cases, comments related to heteronormative and cis-sexist stereotypes surrounding purity and worth, as linked to sexual activity. […] This included implicating notions of “family honour.” This expanded to the idea that sex work and parenting were incompatible:”  **Illustration:** “my gynaecologist said that “nobody will want to marry that much of a used wh*re.” (p. 19)  (European Sex Workers' Rights Alliance 2023) |
|  | **Sexualized behaviours, including sexualized gestures, stealing underwear**  (European Sex Workers' Rights Alliance June 02, 2025, November 11, 2024) | **Finding:** „Some of the most common and prevalent forms of sexualised violence and harassment involved sexist and sexualised comments made by on-duty police officers during routine identity checks, interventions,  and raids at sex work venues. These included offensive gestures and remarks on sex workers’ occupation, appearance, advertisements, as well as explicitly sexualised innuendos and stares. […] One participant recounted an incident in which a police officer stole her underwear during a search.”  **Illustration 1:** “One time, I swear, this cop had his legs open with his hand on his balls, on his dick.”  **Illustration 2:** “I once had in my bag a little nighty and little panties, too. I had all that kind of stuff there in my bag. It disappeared. I saw that he was rummaging through my bag in a side room. I was told that I would get them back at the exit. But when I got to the exit, they had disappeared.”  (p. 23)  (European Sex Workers' Rights Alliance June 02, 2025) |
|  | **Sexual street harassment: verbal harassment particularly on the streets (also often combined with physical violence)**  (Kiernan et al. 2016; Krüsi A et al. 2016; Sherman et al. 2015; Armstrong 2016; Crago 2015; Dawthorne 2023; Dewey and St. Germain 2014; Scorgie and Nakato et al. 2013; Scorgie and Vasey et al. 2013) | **Finding:** “A number of women described pervasive verbal harassment within the context of being out on the street: [...] Beyond police's failure as custodians of women's safety on and off the street, police harassment emerged as an insidious tool of social control, with little connection to women's street work:”  **Illustration:** “They stop and ask you ‘you got an ID on you? What are you standing here for? Are you trying to get picked up? Why don't you get away from here? Where are you walking to?’ First of all, you can't tell me to go in a house and I can take a walk. I can be out jogging. Sometimes they'll tell you if I see you again you're going to jail. For what? I haven't done anything. I could see if you see me get out of a car or you caught me doing something, but you can't just keep harassing me because I'm walking down the street.... Some of them just go, ‘well, slut, you're going to jail.  We're going to take your hooker ass to the jail” (p. 5)  (Sherman et al. 2015) |
|  | **Sexual abuse as defined as “manipulative, psychologically abusive tactics to keep partner [or person] in submissive position of power” (p. 323) by Bagwell-Gray et al. (2015)**  Including threats of outing  (Zarhin and Fox 2017; Crago 2015) | **Finding:** “Fear of losing custody was reported by two other sex workers in Montenegro as the reason for not reporting spousal violence. […] In the above case, the abusive ex-partner’s use of the threat of “outing” a sex worker to police is illustrative of the ways that systemic discrimination by state-actors can be coopted to enforce violence and threats by state and non-state perpetrators.”  **Illustration:** “I have a child. When he was born his father refused to hear about him, so there is only my name on his Birth Certificate. As I am a sex worker, his father was saying that he is a bastard child and that even I don’t know who the father is as I have sex with everybody. But a year after his birth, this man appears and starts beating me up in order to take the child. (…) He was threatening me that he will tell police that I am sex worker and he told me that he will find witnesses for that.” (p. 78)  (Crago 2015)  **Finding:** „She went on to explain that her husband knew she had been a sex worker in the past and used it against her during their disputes, undermining the boundaries between the personal and the professional that she tried hard to retain. After three years of marriage, she had had enough of ‘being treated as a prostitute’ at home and divorced him.”  **Illustration:** “being treated as a prostitute” (p. 1085)  (Zarhin and Fox 2017) |
|  | **Sexual objectification and dehumanization, including a broad range on constructs such as fetishization or paternalizing behaviour**  (Dawthorne 2023; Kloek and Dijkstra 2018; Levine 2021; European Sex Workers' Rights Alliance 2023; Scorgie and Nakato et al. 2013; Scorgie and Vasey et al. 2013; Shepp 2023; Spyrelis and Ibisomi 2022; Zarhin and Fox 2017; Smaniotto Gehlen et al. 2018; Roman 2021) | **Finding:** “Including voyeurism and fetishization of sex work. […] Fetishization of sex workers, contributed to a reported sense of entitlement to sex workers’ bodies, and seeing sex workers as ‘public property’”  **Illustration:** “There was this entitlement that I would be willing to have sex with the person talking, at any time, because of my line of work” (p. 16-17)  (European Sex Workers' Rights Alliance 2023)  **Finding:** “Dehumanisation of sex workers played into hierarchies of perceived ‘deservedness’ of care among healthcare providers”  **Illustration:** “Psych ward staff told me that the hospital is wasting space and money on me.” (p. 24)  (European Sex Workers' Rights Alliance 2023)  **Finding:** “Many sex workers in this research were faced with unnecessary, unwanted and intrusive questions. Respondents were asked, for instance, about how many clients they saw in a week, how much they were being paid, what they were feeling and thinking afterward, what their parents and friends thought of their work, but also what their selfesteem was like. These types of questions were asked by strangers, landlords and representatives of (government) agencies such as the police and the UWV.”  **Illustration:** “I found the questions very ... I felt, like, being dirty and filthy.” (p. 22)  (Kloek and Dijkstra 2018)  **Finding:** “The notion that sex workers were not perceived to be “human” emerged strongly in all study sites, but particularly in east Africa.”  **Illustration:** “[People in the community say] I’mnotahuman being … I am just useless, spoilt and that’s the end of me. They can’t allow me to spoil others. When they see my child they say ‘that’sa prostitute’s child, look at it, child of a prostitute.’” (24 year old female, Kampala)“ (p. 7)  (Scorgie and Vasey et al. 2013) |
|  | **Voyerism**  (Meiliana 2023) | **Finding:** “In addition, the explanation of one of the Ronggeng dancers regarding ‘naughty’ hands led me to question […] As artists, Ronggeng dancers are often abused, physically and verbally, when performing. Physically, they are often subjected to harassment, such as spectators, who try to put money into their chests, kiss them, touch, or squeeze their bodies, and even peek at them while changing clothes. These findings are in line with Caturwati (2019), who describes that drunk male-spectators often harassed Ronggeng dancers by putting money to their kemben while touching and squeezing their breasts. […] Verbally, they often offered to ‘serve’ which refers to sexual intercourse, and offered to be the audience’s mistress.“  **Illustration:** “‘I often met naughty spectators. Sometimes they tried to kiss me, sometimes tried to put money in my chest, and once there was an audience peeking at me while changing costumes.” (p. 12)  (Meiliana 2023) |
| **Technology-facilitated sexual violence** | **Including intimate image abuse, blackmailing with intimate images, non-consensual recording of a sex tapes, impersonation (e.g., police officers posing as clients), unconsented sending of nudes**  (Kuosmanen and Cabo 2021; Ryan and McGarry 2022; Friend 2023; European Sex Workers' Rights Alliance November 11, 2024, June 02, 2025, May 14, 2025; Yaakobovitch et al. 2024) | **Finding:** “A participant in Limerick revealed how she was being blackmailed into sending money to stop the illegal distribution of her photographs, threatening her safety and mental well-being”  **Illustration:** “I’m just feeling so powerless with it all like. The more stuff that goes around like, this guy was blackmailing me basically going “send me more pictures and I’ll delete it” (p. 698)  (Ryan and McGarry 2022) |
| **Sexual neglect** | **Refusal of health care**  (Scorgie and Nakato et al. 2013; Shepp 2023; European Sex Workers' Rights Alliance 2023; Mashumba 2024; Nichols 2014; Bazzi et al. 2019; Ryan and McGarry 2022; Spyrelis and Ibisomi 2022; Khofi et al. 2025) | **Finding:** “Participants cited broad challenges with accessing public-sector facilities that are presumably common to all patients attending these facilities. These included, primarily, long waiting times, high user fees, medicine shortages and inadequate transport to hospitals. But SWs experienced several additional barriers to receiving decent care. Providers were described as “abusive” and “hostile”, at times explicitly withholding treatment, referring SWs unnecessarily, or explicitly blaming SWs for their illnesses.”  **Illustration:** “When I fell sick and went to a health centre and they realised that I was a SW, they did not treat me like a human being. When the health worker came to attend to me she said that I should go to the other health worker and when I reached the other health worker, I was told that he had no time for me. So I left without getting treatment“ (p. 6)  (Scorgie and Nakato et al. 2013) |
|  | **Reduced access to health care**  (Roman 2021; Spyrelis and Ibisomi 2022; European Sex Workers' Rights Alliance 2023; Maher L et al. 2015; European Sex Workers' Rights Alliance November 11, 2024, June 02, 2025; Khofi et al. 2025; Tocci 2024) | **Finding:** “Some women elaborated on how the displacement of sex workers consequent to the police crackdown had disrupted women’s networks, restricting their access not only to condoms, but to medical services such as HIV testing and treatment.”  **Illustration:** “Because the policemen crack down often we cannot earn money. We are sleepless, so we sleep at day time, so I am lazy to go to check my health. I have no feeling to go.“ (p. 108)  (Maher L et al. 2015) |
|  | **Violation of privacy / breach of confidentiality (including outing)**  (European Sex Workers' Rights Alliance 2023; Crago 2015; Bungay and Guta 2018; Scorgie and Nakato et al. 2013) | **Finding:** “Male SWs in particular are often seen as a curiosity by health workers”  **Illustration:** “One time I had a problem and went to a clinic and I explained to the doctor and the doctor was like, ‘Let me come back’ and after five minutes I saw all the nurses coming and peeping in the room looking at me and going back. So when the doctor came back I asked him, ‘Have you said anything to these people? So I don’t have a right to confidentiality between me and you” (p.7-8)  (Scorgie and Nakato et al. 2013) |
|  | **(Fear of) hate crimes based on sexual orientation and/or gender identity**  (Lyons et al. 2017; Dawthorne 2023; Crago 2015; Kloek and Dijkstra 2018; European Sex Workers' Rights Alliance November 11, 2024, June 02, 2025) | **Finding:** “Participants described experiencing violence from clients  because of their gender”  **Illustration:** “[He] almost stabbed me . . . I seen that knife and I jumped out . . . He swung at me, but he never got me. I got out of the truck just in time . . . I think you know ’cause I was trans and he didn’t like it.” (p. 185)  (Lyons et al. 2017) |
|  | **Forced gendered behaviour**  (Nichols 2014; European Sex Workers' Rights Alliance 2023) | **Finding:** “In some cases, forced gendered behavior was accompanied by verbal abuse, primarily by the use of the derogatory term ponnaya. For example, Ranil stated that they are also verbally abused when they are forced to do these things by police: […] This example shows forced gendered behavior occurring in tandem with sexual verbal abuse, specifically targeting their homosexuality in the verbal references to oral and anal sex.”  **Illustration:** “The police…disturb you- come and crack jokes, so put you in a difficult situation only. So that is they insult [you] really badly, tell to dance, tell ‘‘Dance!’’, ‘‘How did y’all give [the ass],’’ they ask, ‘‘how did y’all do [have sex],’’ they ask, ‘‘how do y’all suck the Sirs,’’ so those [things] only [they say]. Listen to those [questions] plenty of times and after giving answers to those only, in the end they remand and put [us] in prison also.” (p. 175)  (Nichols 2014) |
|  | **Further stigma and/or discrimination, including social exclusion**  (Dawthorne 2023; Levine 2021; European Sex Workers' Rights Alliance 2023, May 14, 2025; Scorgie and Nakato et al. 2013; Scorgie and Vasey et al. 2013; Kloek and Dijkstra 2018; Yaakobovitch et al. 2024; Nattabi et al. 2025; Tocci 2024; Panchanadeswaran et al. 2024; Pokharel et al. 2024) | **Finding:** “Rick, despite positive experiences with clients and exploration of same-sex desires, compares himself to his peers, considering the low-status of sex work (and his addiction)”  **Illustration:** “There seems to be a real stigma about it [in London] like, it just seems... people will go on and on about how disgusting it is, and those people are usually first in line. [I] definitely [experience] a little bit of shame because I mean like I’m going on 35 next year and you know all my peers that I grew up with I hang around with are doing shit with their lives and I’m out here getting my dick sucked.” (p. 186)  (Dawthorne 2023) |

References

Aborisade RA. Police abuse of sex workers in Nigeria: evidence from a qualitative study. Police Practice and Research. 2019;20(4):405–19. doi:10.1080/15614263.2018.1500283.

Antwi AA, Ross MW, Markham C. Occupational Health and Safety among Female Commercial Sex Workers in Ghana: A Qualitative Study. SEXES. 2023;4(1):26–37. doi:10.3390/sexes4010003.

Armstrong L. “Who’s the Slut, Who’s the Whore?”. Feminist Criminology. 2016;11(3):285–303. doi:10.1177/1557085115588553.

Bagwell-Gray ME, Messing JT, Baldwin-White A. Intimate Partner Sexual Violence: A Review of Terms, Definitions, and Prevalence. Trauma Violence Abuse. 2015;16(3):316–35. doi:10.1177/1524838014557290.

Bazzi AR, Yotebieng K, Otticha S, Rota G, Agot K, Ohaga S, Syvertsen JL. PrEP and the syndemic of substance use, violence, and HIV among female and male sex workers: a qualitative study in Kisumu, Kenya. Journal of the International AIDS Society 2019. doi:10.1002/jia2.25266.

Beaujolais B, Kaloga M, Karandikar S, Gezinski LB, Kadambari P, Maskey K. Client-Perpetrated Violence Toward Female Sex Workers in Kathmandu. Violence Against Women. 2020;26(2):249–67. doi:10.1177/1077801219832117.

Benner BE. HIV Vulnerability Among Survival Sex Workers Through Sexual Violence and Drug Taking in a Qualitative Study From Victoria, Canada, With Additional Implications for Pre-exposure Prophylaxis for Sex Workers. Frontiers in Sociology 2022. doi:10.3389/fsoc.2021.714208.

Bungay V, Guta A. Strategies and Challenges in Preventing Violence Against Canadian Indoor Sex Workers. Am J Public Health. 2018;108(3):393–8. doi:10.2105/AJPH.2017.304241.

Cange CW, LeBreton M, Saylors K, Billong S, Tamoufe U, Fokam P, Baral S. Female sex workers’ empowerment strategies amid HIV-related socioeconomic vulnerabilities in Cameroon. Culture, Health and Sexuality. 2017;19(10):1053–65. doi:10.1080/13691058.2017.1291993.

Cange CW, Wirtz AL, Ky-Zerbo O, Lougue M, Kouanda S, Baral S. Effects of traumatic events on sex workers’ mental health and suicide intentions in Burkina Faso: A trauma-informed approach. Sexual Health. 2019;16(4):348–57. doi:10.1071/SH17213.

Cepeda A, Nowotny KM. A border context of violence: Mexican female sex workers on the U.S.-Mexico border. Violence Against Women. 2014;20(12):1506–31. doi:10.1177/1077801214557955.

Dawthorne N. Intelligible variability: Narratives of male sex work in London Ontario Canada: ProQuest Information & Learning; 2023.

Decker MR, Pearson E, Illangasekare SL, Clark E, Sherman SG. Violence against women in sex work and HIV risk implications differ qualitatively by perpetrator. BMC Public Health 2013. doi:10.1186/1471-2458-13-876.

Dewey S, St. Germain T. “It Depends on the Cop:” Street-Based Sex Workers’ Perspectives on Police Patrol Officers. Sexuality research & social policy : journal of NSRC : SR & SP. 2014;11(3):256–70. doi:10.1007/s13178-014-0163-8.

Eshetu HB, Zewdie A, Girma E, Kassie A, Adugna A, Nigusie A, Handebo S. “Many People Do Not Consider Us Human” Violence Against Commercial Sex Workers in Gondar City: A Phenomenological Study. SAGE Open 2025. doi:10.1177/21582440251324724.

Friend J. Digital privacy is a sexual health necessity: a community-engaged qualitative study of virtual sex work and digital autonomy in Senegal. Sexual and Reproductive Health Matters 2023. doi:10.1080/26410397.2023.2272741.

Katumba KR, Haumba M, Mayanja Y, Machira YW, Gafos M, Quaife M, et al. Understanding the contexts in which female sex workers sell sex in Kampala, Uganda: a qualitative study. BMC Womens Health. 2024;24(1):371. doi:10.1186/s12905-024-03216-7.

Katz KR, McDowell M, Green M, Jahan S, Johnson L, Chen M. Understanding the Broader Sexual and Reproductive Health Needs of Female Sex Workers in Dhaka, Bangladesh. Int Perspect Sex Reprod Health. 2015;41(4):182–90. doi:10.1363/4118215.

Khofi L, Manderson L, Moyer E. Food insecurity, intimate partner violence, and barriers to sexual and reproductive health care among women in Lorentzville, South Africa. Soc Sci Med. 2025;369:117785. doi:10.1016/j.socscimed.2025.117785.

Kiernan B, Mishori R, Masoda M. ‘There is fear but there is no other work’: a preliminary qualitative exploration of the experience of sex workers in eastern Democratic Republic of Congo. Culture, Health and Sexuality. 2016;18(3):237–48. doi:10.1080/13691058.2015.1073790.

Krüsi A, Pacey K, Bird L, Taylor C, Chettiar J, Allan S, et al. Criminalisation of clients: Reproducing vulnerabilities for violence and poor health among street-based sex workers in Canada - A qualitative study. BMJ Open 2014. doi:10.1136/bmjopen-2014-005191.

Krüsi A, Kerr T, Taylor C, Rhodes T, Shannon K. ‘They won’t change it back in their heads that we’re trash’: the intersection of sex work-related stigma and evolving policing strategies. Sociol Health Illn. 2016;38(7):1137–50. doi:10.1111/1467-9566.12436.

Kuosmanen J, Cabo A de. Men Selling Sex to Men in Sweden: Balancing Safety and Risk. J Interpers Violence. 2021;36(5-6):NP2601-NP2623. doi:10.1177/0886260518762448.

Kyriakakis S, Compton-Almo C, Goddard-Durant S. Subsistence and Survival: Strategies Women in the Republic of Barbados Engaged in Transactional Sex Work Employ to Stay Safe. Journal of Aggression, Maltreatment & Trauma. 2024;33(10):1280–98. doi:10.1080/10926771.2024.2332604.

Levine EC. Female-to-male to mistress: A layered account of layered performances. Sexualities. 2021;24(1-2):252–75. doi:10.1177/1363460720931329.

Lim S, Peitzmeier S, Cange C, Papworth E, LeBreton M, Tamoufe U, et al. Violence against female sex workers in Cameroon: accounts of violence, harm reduction, and potential solutions. J Acquir Immune Defic Syndr. 2015;68 Suppl 2:S241-7. doi:10.1097/QAI.0000000000000440.

Lyons T, Krüsi A, Pierre L, Kerr T, Small W, Shannon K. Negotiating Violence in the Context of Transphobia and Criminalization: The Experiences of Trans Sex Workers in Vancouver, Canada. Qualitative health research. 2017;27(2):182–90. doi:10.1177/1049732315613311.

Maher L, Dixon T, Phlong P, Mooney-Somers J, Stein E, Page K. Conflicting Rights: How the Prohibition of Human Trafficking and Sexual Exploitation Infringes the Right to Health of Female Sex Workers in Phnom Penh, Cambodia. Health Hum Rights. 2015;17(1):E102-13.

Marlow HM, Shellenberg K, Yegon E. Abortion services for sex workers in Uganda: successful strategies in an urban clinic. Culture, Health and Sexuality. 2014;16(8):931–43. doi:10.1080/13691058.2014.922218.

Mashumba L. ‘Blowjobs are Jobs Too’: An Exploratory Study into Victimization Experiences of Male Sex Workers in Botswana. Sexuality and Culture. 2024;28(1):54–70. doi:10.1007/s12119-023-10105-y.

Matheson C, Bon L, Bowman L, Hannah A, Macleod K. Vulnerability, Risk and Harm for People Who Use Drugs and Are Engaged in Transactional Sex: Learning for Service Delivery. Int J Environ Res Public Health 2022. doi:10.3390/ijerph19031840.

Mbonye M, Rutakumwa R, Weiss H, Seeley J. Alcohol consumption and high risk sexual behaviour among female sex workers in Uganda. African Journal of AIDS Research. 2014(2):145–51. doi:10.2989/16085906.2014.927779.

Meiliana S. Cultural violence: feminist power analysis on Ronggeng performing art. Research in Dance Education 2023. doi:10.1080/14647893.2023.2258804.

Nattabi J, Sensoy Bahar O, Nabayinda J, Nabunya P, Kiyingi J, Kizito S, et al. Crossroads of choice: a qualitative study of the factors influencing decisions to transition from sex work among women engaged in sex work in Southern Uganda. BMC Womens Health. 2025;25(1):196. doi:10.1186/s12905-025-03631-4.

Nelson E-U. Structural inequities, HIV vulnerability and women’s agency: Street-based sex workers in Nigeria. Global Public Health. 2020:1800–9. doi:10.1080/17441692.2020.1791211.

Nichols AJ. Intersections of gender and sexuality in police abuses against transgender sex workers in Sri Lanka. In: Nichols AJ, ed. Handbook of LGBT Communities, Crime, and Justice; 2014. p. 165–182. doi:10.1007/978-1-4614-9188-0_9.

Okanlawon K, Adebowale AS, Titilayo A. Sexual hazards, life experiences and social circumstances among male sex workers in Nigeria. Culture, health & sexuality. 2013;15 Suppl:22–33. doi:10.1080/13691058.2012.754053.

Onyango MA, Adu-Sarkodie Y, Agyarko-Poku T, Asafo MK, Sylvester J, Wondergem P, et al. “It’s all about making a life”: Poverty, HIV, violence, and other vulnerabilities faced by young female sex workers in Kumasi, Ghana. Journal of Acquired Immune Deficiency Syndromes. 2015;68:S131-S137. doi:10.1097/QAI.0000000000000455.

Oselin SS, Blasyak A. Contending with Violence: Female Prostitutes’ Strategic Responses on the Streets. Deviant Behavior. 2013;34(4):274–90. doi:10.1080/01639625.2012.735896.

Panchanadeswaran S, Vijayakumar G, Chacko S, Subramaniam S, Dasari S, Lee S, Brazda M. “A little love is enough for me to live my life…”: Precarity and resilience among older sex workers in India. J Women Aging. 2024;36(5):410–26. doi:10.1080/08952841.2024.2360259.

Panneh M, Gafos M, Nyariki E, Liku J, Shah P, Wanjiru R, et al. Mental health challenges and perceived risks among female sex Workers in Nairobi, Kenya. BMC Public Health. 2022;22(1):2158. doi:10.1186/s12889-022-14527-5.

Pokharel HS, Myia SD, Chalise A, Paudel S. Exploring commercial sex work among transgender women in Nepal: Contributors and stigma-A mixed-method study. PLoS One. 2024;19(12):e0314619. doi:10.1371/journal.pone.0314619.

Preble K, Magruder K, Cimino AN. It’s like being an electrician, you’re gonna get shocked’: Differences in the perceived risks of indoor and outdoor sex work and its impact on exiting. 2021;14(5):625–46. doi:10.1080/15564886.2019.1630043.

Reed E, West BS, Frost E, Salazar M, Silverman JG, McIntosh CT, et al. Economic vulnerability, violence, and sexual risk factors for HIV among female sex workers in Tijuana, Mexico. AIDS and Behavior 2022. doi:10.1007/s10461-022-03670-0.

Roman N. Prostitution in Havana, Cuba, a conflict analysis of ‘The phenomenon of Jineterismo in Havana, Cuba’ A narrative study: ProQuest Information & Learning; 2021.

Ryan P, McGarry K. ‘I miss being honest’: sex workers’ accounts of silence and disclosure with health care providers in Ireland. Culture, Health and Sexuality. 2022;24(5):688–701. doi:10.1080/13691058.2021.1879271.

Scorgie F, Vasey K, Harper E, Richter M, Nare P, Maseko S, Chersich MF. Human rights abuses and collective resilience among sex workers in four African countries: A qualitative study. Globalization and Health 2013. doi:10.1186/1744-8603-9-33.

Scorgie F, Nakato D, Harper E, Richter M, Maseko S, Nare P, et al. ‘We are despised in the hospitals’: Sex workers’ experiences of accessing health care in four African countries. Culture, Health and Sexuality. 2013;15(4):450–65. doi:10.1080/13691058.2012.763187.

Shepp V. Seeking support under the state: Aex worker’s experiences navigating gender-based violence services: ProQuest Information & Learning; 2023.

Sherman SG, Footer K, Illangasekare S, Clark E, Pearson E, Decker MR. “What makes you think you have special privileges because you are a police officer?” A qualitative exploration of police’s role in the risk environment of female sex workers. AIDS Care - Psychological and Socio-Medical Aspects of AIDS/HIV. 2015;27(4):473–80. doi:10.1080/09540121.2014.970504.

Siegel K, Cabán M, Brown-Bradley CJ, Schrimshaw EW. Experiences of interpersonal violence among a diverse sample of male sex workers. Culture, Health and Sexuality 2023a. doi:10.1080/13691058.2023.2231049.

Siegel K, Cabán M, Brown-Bradley CJ, Schrimshaw EW. Male Sex Workers’ Strategies to Manage Client-Related Risks of Violence. J Interpers Violence. 2023b;38(19-20):10814–38.

Simmons B, Syvertsen JL. Learning from women who trade sex in Kenya about the antiblackness of Global Health. Social Science and Medicine 2022. doi:10.1016/j.socscimed.2022.115246.

Smaniotto Gehlen RG, Da Costa MC, Arboit J, Da Silva EB. Instances of vulnerability to violence experienced by female sex workers: A case study. Ciencia y Enfermeria 2018. doi:10.4067/s0717-95532018000100208.

Spyrelis A, Ibisomi L. “It is just a lot to deal with”: A qualitative study exploring the sexual and reproductive health needs of a sample of female sex workers in six locations in Southern Africa. African Journal of Reproductive Health. 2022;26(5):72–80. doi:10.29063/ajrh2022/v26i5.8.

Tocci B. The Mental Health Management of Individuals in Sex Work. Milwaukee, Wisconsin, USA: Marquette University; 2024.

Twizelimana D, Muula AS. Hiv and aids risk perception among sex workers in semi-urban blantyre, Malawi. Tanzania Journal of Health Research 2015. doi:10.4314/thrb.v17i3.5.

Wanjiru R, Nyariki E, Babu H, Lwingi I, Liku J, Jama Z, et al. Beaten but not down! Exploring resilience among female sex workers (FSWs) in Nairobi, Kenya. BMC Public Health 2022. doi:10.1186/s12889-022-13387-3.

Yaakobovitch T, Bensimon M, Idisis Y. A qualitative analysis of male actors in amateur pornography: motivations, implications and challenges. Curr Psychol 2024. doi:10.1007/s12144-024-06329-2.

Zarhin D, Fox N. ‘Whore stigma’ as a transformative experience: altered cognitive expectations among Jewish-Israeli street-based sex workers. Culture, Health and Sexuality. 2017;19(10):1078–91. doi:10.1080/13691058.2017.1292367.
