# Supplementary material for: Systematic review of sexual violence against sex workers: implications for mental and sexual health
Source: BMC Public Health. 2026 Jun 30;26:2126. doi: 10.1186/s12889-026-28204-4 (PMC13360242; doi:10.1186/s12889-026-28204-4)
Supplement: Supplementary file 5 — Additional file 5. Sexual violence prevalences by WHO region. [file 12889_2026_28204_MOESM5_ESM.docx]

**Systematic review of sexual violence against sex workers: Implications for mental and sexual health**

**Additional file 5**

Marie Püffel1, İsmail Orbay2*, Ira Salo3*, Henriette Berg1*, Lea Hasanagic1*, Elisa Ruiz Burga4, Thérèse Bernier5, Nina Heinrichs1

1Bielefeld University | Department of Psychology | Bielefeld | Germany

2Protestant University of Applied Sciences Berlin | Department of Social Work | Berlin | Germany

3University of Turku | Faculty of Law | Turku | Finland

4University College London | Institute of Global Health | London | United Kingdom

5George Brown Polytechnic | Faculty of Applied Science, Construction and Engineering Technology | Toronto | Canada

* Authors had same amount of contribution to paper

**Table A Lifetime, past year and past six months prevalence of sexual violence only divided by WHO assigned region**

| Region | | Context | Lifetime | | Past year | | Past 6 months | |
| --- | --- | --- | --- | --- | --- | --- | --- | --- |
|  | |  | Prevalence  (95% CI), % | *n*/*k* (*I^2^*) | Prevalence  (95% CI), % | *n*/*k* (*I^2^*) | Prevalence  (95% CI), % | *n*/*k* (*I^2^*) |
| Low- and middle-income countries and areas in: | |  |  |  |  |  |  |  |
|  | African Region | Overall | 23.3 [17.3 - 30.5] | 74/34 (97.3 %) | 19.4 [14.7 - 25.2] | 33/20 (98.6 %) | 20.6 [13.2 - 30.6] | 23/14 (98.6 %) |
|  |  | Workplace | 20.3 [9.7 - 37.6] | 13/8 (96.9 %) | 27.2 [4.1 - 76.4] | 3/3 (97.9 %) | 20.2 [6.3 - 48.8] | 7/5 (98 %) |
|  |  | Partner | 18.0 [7.6 - 36.9] | 8/8 (96.1 %) | 21.5 [7.6 - 47.6] | 7/5 (94.1 %) | 29.7 [9.5 - 63.1] | 3/3 (95.9 %) |
|  |  | Police | 8.7 [1.6 - 36.0] | 7/7 (94.7 %) | 6.0 [0.8 - 33.9] | 3/3 (98.4 %) | 3.6 [3.1 - 4.2] | - |
|  |  | Other | 13.3 [4.0 - 35.8] | 11/7 (96 %) | 30.3 [1.5 - 92.7] | 3/3 (98.2 %) | - | - |
|  | Region of the Americas | Overall | 32.6 [23.6 - 43.1] | 18/15 (96 %) | - | - | 16.0 [9.4 - 25.9] | 9/6 (93 %) |
|  |  | Workplace | 15.9 [0.1 - 98.0] | 2/2 (93 %) | - | - | 9.5 [2.8 - 28.1] | 4/3 (93.6 %) |
|  |  | Partner | 17.3 [12.2 - 22.4] | - | - | - | - | - |
|  |  | Police | - | - | - | - | 24.0 [0.1 - 98.7] | 2/1 (94.9 %) |
|  |  | Other | - | - | - | - | 25.3 [20.7 - 29.9] | - |
|  | Eastern Mediterranean Region | Overall | 33.6 [11.4 - 66.4] | 6/3 (94.6 %) | 16.8 [16.0 - 17.7] | 2/1 (-24295.4 %) | 24.7 [6.0 - 62.8] | 3/2 (97.1 %) |
|  |  | Workplace | 4.2 [1.8 - 6.6] | - | 16.9 [14.9 - 19.0] | - | - | - |
|  |  | Partner | 30.4 [24.9 - 36.0] | - | - | - | - | - |
|  |  | Other | 37.3 [31.4 - 43.1] | - | - | - | - | - |
|  | European Region | Overall | 33.6 [0.0 - 100.0] | 2/2 (99.4 %) | - | - | 22.8 [13.3 - 36.2] | 5/2 (70.8 %) |
|  |  | Workplace | - | - | - | - | 21.5 [17.2 - 25.7] | - |
|  |  | Partner | - | - | - | - | 22.1 [8.5 - 46.2] | 3/2 (81.9 %) |
|  |  | Police | 6.4 [4.4 - 8.5] | - | - | - | - | - |
|  |  | Other | - | - | - | - | 27.0 [22.6 - 31.4] | - |
|  | South-East Asia Region | Overall | 32.6 [15.1 - 56.8] | 10/6 (95.3 %) | 7.6 [2.2 - 23.0] | 12/6 (95 %) | 5.9 [4.0 - 8.7] | 5/2 (52.6 %) |
|  |  | Workplace | 22.3 [0.1 - 99.1] | 2/2 (17.4 %) | 1.3 [0.0 - 100.0] | 2/1 (92.7 %) | 8.7 [6.5 - 10.9] | - |
|  |  | Partner | - | - | 14.7 [12.6 - 16.8] | - | 6.1 [2.2 - 16.0] | 2/1 (-) |
|  |  | Police | - | - | 0.9 [0.4 - 1.5] | - | - | - |
|  |  | Other | - | - | 0.7 [0.0 - 100.0] | 2/1 (82.9 %) | - | - |
|  | Western Pacific Region | Overall | 14.8 [7.6 - 26.8] | 14/8 (94.8 %) | 27.6 [0.0 - 100.0] | 2/1 (95.9 %) | 12.6 [1.1 - 64.2] | 5/3 (89.5 %) |
|  |  | Workplace | 18.7 [1.2 - 81.4] | 4/3 (97.7 %) | 42.6 [37.1 - 48.1] | - | 16.2 [0.5 - 88.2] | 3/3 (91.5 %) |
|  |  | Partner | 18.7 [3.7 - 58.2] | 2/2 (-) | 16.1 [12.0 - 20.2] | - | 4.8 [0.0 - 100.0] | 2/2 (88.2 %) |
|  |  | Other | 4.7 [0.5 - 30.2] | 3/2 (57.1 %) | - | - | - | - |
| High-income countries and areas | | Overall | 31.5 [23.8 - 40.3] | 20/11 (87.7 %) | 20.3 [6.3 - 49.2] | 10/5 (97.9 %) | 7.9 [5.7 - 10.7] | 26/10 (88.9 %) |
|  |  | Workplace | 28.3 [12.6 - 51.9] | 5/3 (87.5 %) | 35.5 [2.5 - 92.3] | 2/1 (74.7 %) | 9.3 [4.6 - 17.8] | 9/6 (88.6 %) |
|  |  | Partner | 29.2 [1.1 - 93.9] | 2/2 (59.7 %) | 25.9 [0.0 - 100.0] | 2/2 (95.5 %) | 9.1 [3.6 - 21.4] | 6/5 (80.8 %) |
|  |  | Police | 28.2 [23.2 - 33.2] | - | 6.8 [5.5 - 8.1] | - | 4.8 [4.0 - 5.8] | 6/4 (-) |
|  |  | Other | 47.1 [39.3 - 54.9] | - | - | - | 2.4 [1.4 - 3.4] | - |
| Unspecified region / area ^a^ | | Overall | 10.0 [0.4 - 73.9] | 3/1 (90.1 %) | 13.0 [9.2 - 16.8] | - | - | - |
|  |  | Police | 10.0 [0.4 - 73.9] | 3/1 (90.1 %) | 13.0 [9.2 - 16.8] | - | - | - |

Note. CI = Confidence Interval, n = number of effect sizes, k = number of studies, I^2^ = Heterogeneity, I^2^ = “-“ : too little studies to calculate I^2^

^a^: Due to multiple countries across regions

Sexual violence was either measured with multiple questions, was not clearly defined or defined as completed forced sexual intercourse.
