# Supplementary material for: Systematic review of sexual violence against sex workers: implications for mental and sexual health
Source: BMC Public Health. 2026 Jun 30;26:2126. doi: 10.1186/s12889-026-28204-4 (PMC13360242; doi:10.1186/s12889-026-28204-4)
Supplement: Supplementary file 6 — Additional file 6. Prevalences of health-related symptoms. [file 12889_2026_28204_MOESM6_ESM.docx]

**Systematic review of sexual violence against sex workers: Implications for mental and sexual health**

**Additional file 6**

Marie Püffel1, İsmail Orbay2*, Ira Salo3*, Henriette Berg1*, Lea Hasanagic1*, Elisa Ruiz Burga4, Thérèse Bernier5, Nina Heinrichs1

1Bielefeld University | Department of Psychology | Bielefeld | Germany

2Protestant University of Applied Sciences Berlin | Department of Social Work | Berlin | Germany

3University of Turku | Faculty of Law | Turku | Finland

4University College London | Institute of Global Health | London | United Kingdom

5George Brown Polytechnic | Faculty of Applied Science, Construction and Engineering Technology | Toronto | Canada

* Authors had same amount of contribution to paper

**Table A Prevalence of mental and sexual health symptoms**

| Outcome | Prevalence  (95% CI), % | *n/k* (I^2^) |
| --- | --- | --- |
| **Mental health conditions** |  |  |
| Depressive symptoms | 39.6 [29.6 - 49.7] | 21/14 (99.7 %) |
| Post-traumatic stress symptoms | 39.9 [21.9 - 57.9] | 6/4 (99.7 %) |
| Suicidality-related symptoms | 30.4 [12.3 - 48.6] | 7/6 (98.9 %) |
| Other-related mental health symptoms | 53.3 [-137.8 - 244.4] | 2/2 (98.2 %) |
| **Alcohol and other drug use** |  |  |
| Alcohol use | 40.9 [30.7 - 51.1] | 22/19 (99.8 %) |
| Drug use | 31.6 [22.4 - 40.9] | 30/27 (100 %) |
| **STI/HIV infection** |  |  |
| HIV | 21.5 [15.6 - 27.5] | 35/29 (99.6 %) |
| Other STI-related symptoms | 23.8 [17.0 - 30.5] | 18/16 (98.5 %) |
| **Reproductive health related conditions** |  |  |
| Abortion | 33.9 [17.2 - 50.7] | 6/6 (99.2 %) |
| (Unwanted or unplanned) Pregnancy | 42.7 [-11.6 - 96.9] | 3/3 (99.1 %) |

Note. CI = Confidence interval, n = number of effect sizes, k = number of studies, I^2^ = Heterogeneity, STI = sexual transmitted infection, HIV = Human Immunodeficiency Virus

Timeframe varying, often within last weeks
